# Genetic Identification of Lamprey Genera and Anadromous Ecotypes in Watersheds of the Northeastern Pacific Ocean

**DOI:** 10.1111/eva.70108

**Published:** 2025-05-10

**Authors:** G. S. Silver, R. T. Lampman, N. Percival, N. Timoshevskaya, J. J. Smith, K. T. Bentley, J. Wade, S. R. Narum, J. E. Hess

**Affiliations:** ^1^ Columbia River Inter‐Tribal Fish Commission Portland Oregon USA; ^2^ Yakama Nation Fisheries Resource Management Program Pacific Lamprey Project Toppenish Washington USA; ^3^ Nisga'a Fisheries and Wildlife, Nisg̱a'a Lisims Government Gitlax̱t'aamiks British Columbia Canada; ^4^ Department of Biology University of Kentucky Lexington Kentucky USA; ^5^ Washington Department of Fish & Wildlife Ridgefield Washington USA; ^6^ Fundy Aqua Services Inc. Nanoose Bay British Columbia Canada; ^7^ Columbia River Inter‐Tribal Fish Commission Hagerman Idaho USA

**Keywords:** conservation genetics, ecological genetics, genomics/proteomics, life history evolution, speciation

## Abstract

Nonparasitic, nonmigratory Western Brook Lamprey (WBL; *Lampetra ayresii* ), and parasitic, anadromous Western River Lamprey (WRL; *L. ayresii* ) are sympatric lampreys that likely represent different life history variations of a single species. Novel genetic tools are critical for differentiating WBL and WRL, whose larvae preclude morphological identification (ID) and will enable comprehensive assessment of imperiled native lampreys of the Northeastern Pacific (including WBL, WRL, and Pacific Lamprey, *Entosphenus tridentatus* ). We developed 47 candidate single nucleotide polymorphism (SNP) markers using whole genome resequencing of WBL (*N* = 24) and WRL (*N* = 15) from Ksi Ts'oohl Ts'ap Creek (Nass River, British Columbia, Canada) which are likely ecotypes distinguished by few divergent SNPs across multiple chromosomes. We used five novel candidate SNPs to perform genetic ID of WBL and WRL ecotypes in collections of mixed native lampreys from lower Columbia River tributaries (*N* = 1474), Ksi Ts'oohl Ts'ap Creek (*N* = 352), and ocean phase WRL from the Georgia Basin (Salish Sea, British Columbia, Canada; *N* = 91). Two previously published SNPs were used to ID genera, *Entosphenus* versus *Lampetra*. Morphological ID utilized photographs collected from a subset of genotyped lampreys, and high concordance was demonstrated between ID methods for genera (99%) and *Lampetra* ecotypes (> 98%). We characterized spatial and temporal composition of lamprey genera and ecotypes surveyed across NE Pacific tributaries under the expectation these compositions would be similar across nearby sites and across years at the same site. Proportions of lamprey genera were highly variable within regions and across years; however, *Lampetra* ecotypic proportions were spatially and temporally stable. WRL were rare in lower Columbia tributaries (~1% average rate among *Lampetra*) and common further north (> 40% of *Lampetra*). Genetic ID methods are powerful monitoring tools that create the novel ability to ascertain genera and ecotypes regardless of life stage, while increasing the efficiency of surveys by eliminating time‐intensive morphological data collection.

## 1 Introduction

Lamprey populations are in decline worldwide, and conservation concerns for lamprey species have increased in recent years. In Western North America, Pacific Lamprey ( *Entosphenus tridentatus* ) has experienced declines in abundance and constrictions in range over recent decades (Renaud 1997; Luzier et al. 2011). The conservation status of other native lampreys in NE Pacific tributaries, specifically Western Brook Lamprey (WBL, *Lampetra ayresii* ; formerly *Lampetra richardsoni* , Carim et al. 2023) and Western River Lamprey (WRL, *L. ayresii*, Carim et al. 2023), is unclear and hindered by difficulty differentiating the two taxa genetically and morphologically. In fact, new evidence suggests that these nominal species should be considered alternate life‐history types or ecotypes rather than separate species due to their polyphyletic relationships (Docker 2009; Carim et al. 2023, 2024). Uncertainty in species boundaries and inadequate genetic and morphological identification (ID) methods complicate assessments of status and trends in abundance and distribution of these three native lampreys and may hinder conservation and management efforts in tributaries across their ranges. Thus, novel methods of identification of these native lampreys are needed.

Existing morphological and genetic ID methods for distinguishing among the three co‐occurring focal native lampreys in the NE Pacific have limitations in their application that must be overcome if they are to be used for effective management of lampreys. Challenges with morphological ID are largely due to difficulty differentiating among lampreys at early life stages (i.e., prior to metamorphosis). Anadromous Pacific Lamprey and resident WBL are sympatric in many of the rivers and streams where they persist in Western North America (Scott and Crossman 1973; Potter et al. 2015; Clemens and Wade 2023), while the range of the anadromous WRL is less understood due to limited observational data (USFWS data clearing house, https://gis‐fws.opendata.arcgis.com/). The larval form of these taxa is generally the most frequently observed life stage in freshwater systems where they may persist for as many as 11 years (Snake River Pacific Lamprey, Hess et al. 2022) but more commonly 4–6 years (Dawson et al. 2015) before metamorphosing to eyed migratory juveniles (Pacific Lamprey and WRL) or short‐lived resident adults (WBL). Morphological ID of larvae to the genus level (i.e., *Entosphenus* [Pacific Lamprey] vs. *Lampetra* [WBL and WRL]) can be made using nuanced pigmentation patterns in the caudal region (Richards et al. 1982; Goodman et al. 2009; Lampman 2018). However, accurate and efficient larval ID requires training and repetition, which is seldom provided to fisheries field staff whose primary objectives are monitoring salmonids. Two additional factors further complicate larval lamprey morphological ID: (1) genus ID of small (i.e., young) larvae (less than approximately 50 mm in total length) cannot be reliably made morphologically (Goodman et al. 2009; Docker et al. 2016) as differences in caudal pigmentation patterns have not yet developed, and (2) within the genus *Lampetra*, larvae of WBL and WRL are morphologically identical and cannot be differentiated (Goodman et al. 2009). Finally, although morphological differentiation of metamorphosed juvenile Pacific Lamprey and juvenile/adult WRL can be reliably done using dentition patterns, morphological similarities and overlap in body size may present difficulty to untrained staff who do not regularly encounter these taxa.

Second, current genetic ID methods are inadequate for separating all three focal lampreys. Genetic ID is available for genus determination (*Entosphenus* vs. *Lampetra*) using either nuclear (single nucleotide polymorphisms [SNPs], Hess et al. 2015; microsatellites, Docker et al. 2016) or mitochondrial DNA markers (Goodman et al. 2009; Boguski et al. 2012). However, the nominal taxa, WRL and WBL, are polyphyletic based on mitochondrial data within the genus *Lampetra* (Auringer et al. 2023; Carim et al. 2023, 2024), and therefore, WRL cannot be uniquely mitochondrially barcoded. Further, previous attempts to develop > 200 diagnostic SNPs on nuclear DNA have also been unsuccessful at distinguishing WRL from WBL (Hess et al. 2015). Combined evidence from the lack of reciprocal monophyly of WRL and WBL based on mitochondrial DNA (Carim et al. 2023) and the fact that diagnostic markers have not been found to date despite the hundreds of loci surveyed in genetic studies (e.g., Hess et al. 2015) supports the hypothesis that WRL and WBL are life history variants rather than reproductively isolated lineages (Carim et al. 2024). Similar pairs of these life history variants (anadromous/parasitic vs. resident/nonparasitic; “paired species”) have been described in lampreys from other parts of the world (Docker and Potter 2019). A greatly increased number of genetic markers would have to be surveyed (e.g., millions of SNPs via whole genome resequencing data) to overcome the challenge for developing a genetic ID for WRL and WBL, if we assume they are life history variants within one species. A high‐resolution genomic survey would make it possible to find relatively small genomic regions that are directly involved or tightly linked with genes that regulate the different life histories exhibited by WRL and WBL; life history variants distinguished by genes are referred to as ecotypes (reviewed by Clemens and Schreck 2021). In fact, this type of survey was recently used to successfully develop a genetic ID for a lamprey paired species in Europe, the European Brook Lamprey ( *Lampetra planeri* ) and European River Lamprey ( *L. fluviatilis* , Souissi et al. 2022). The candidate SNPs developed by Souissi et al. (2022) were used to reliably differentiate *L. planeri* from *L. fluviatilis* ecotypes at all life history stages. Despite sharing the genus name *Lampetra*, the taxonomic status of this genus has not been consistently defined and varies depending on whether based strictly on mtDNA (new genus for Western North American *Lampetra* may be needed based on how diverged its monophyletic branch is from the branch represented by European *Lampetra*, Carim et al. 2024) or nuclear DNA (first demonstration that Western North American “Pacific” *Lampetra* is sister to European “Atlantic” *Lampetra*; Hughes et al. 2025). The fact that Western North American “Pacific” *Lampetra* occupies a monophyletic group is consistent across these studies, however, this group has either diverged for a relatively short period of time and is part of a single Holarctic lineage of *Lampetra* (Hughes et al. 2025) or diverged from the European *Lampetra* lineage over a long period of time that justifies its own genus (newly proposed genus *Occidentis*, Carim et al. 2024). Importantly for this study, to date, no such SNP markers have been developed that can distinguish the paired species, WBL and WRL, in Western North American *Lampetra*.

We aimed to develop novel SNPs for genetic ID of WBL and WRL ecotypes using a study site in which these ecotypes were sympatric. In a sympatric river system, a genomic survey to screen for candidate SNPs is less likely to be confounded by population structure that can arise from physical barriers to gene flow (Souissi et al. 2022). Thus, genetic differences exhibited between WBL and WRL would be more likely associated with their life history rather than geographical isolation. A suitable Western North American *Lampetra* study population was located in Ksi Ts'oohl Ts'ap Creek (Nass River, British Columbia, Canada), where WBL and WRL were described as recently derived (Beamish et al. 2016). We used a set of juvenile/adult WBL (*N* = 24) and WRL (*N* = 15) specimens from Ksi Ts'oohl Ts'ap Creek that were morphologically identified to each group with high confidence to develop candidate SNPs. We performed whole genome resequencing (WGS) to survey these specimens to identify highly divergent SNPs and developed a novel candidate SNP assay as a genetic ID for these two ecotypes.

We implemented a collaborative lamprey survey effort using the following two methods for identifying lampreys incidentally collected by field crews during salmonid focused monitoring in the NE Pacific: morphological ID using photographs of lampreys and genetic ID made from fin tissue samples. Specifically, our primary objectives were to: (1) develop and validate a novel multi‐SNP marker to differentiate *Lampetra* ecotypes, WBL and WRL, using Ksi Ts'oohl Ts'ap Creek specimen and WGS data; (2) test concordance of morphological and genetic IDs of lamprey genera and *Lampetra* ecotypes (includes both the Ksi Ts'oohl Ts'ap Creek specimen and additional post‐metamorphic samples across a broader geographic range); (3) test for spatial and temporal correlations of genetic‐ID‐based composition of lamprey genera and *Lampetra* ecotypes (utilized the entire dataset of genotypes including larvae without morphological ID); and (4) test for evidence of population structure between *Lampetra* ecotypes using the WGS data. These objectives were guided by the following expectations: (i) SNPs found to differentiate WBL and WRL ecotypes would appear concentrated in narrow genomic regions, (ii) concordance between morphological and genetic IDs would be high if the genetic IDs were accurate, (iii) WRL would have a more restricted northern distribution compared to Pacific Lamprey, which are relatively ubiquitous in NE Pacific tributaries (US Fish and Wildlife Service (USFWS) 2018), (iv) anadromous WRL would occur closer to the ocean (i.e., coastal distribution) relative to non‐anadromous WBL (i.e., interior distribution), and (v) there would be no evidence of population structure between *Lampetra* ecotypes if they were randomly mating life history variants within a single population.

## 2 Methods

### 2.1 Sample Collection

**FIGURE 1:**
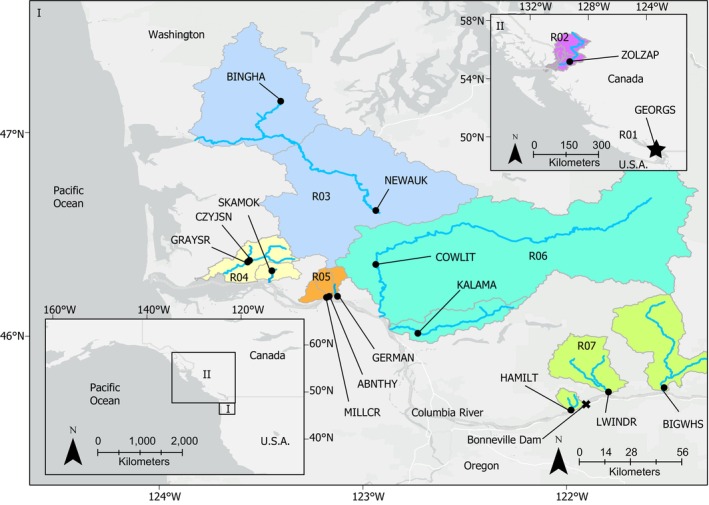
Map of collections within Washington, United States (panel I) and B.C., Canada (panel II) and large scale (bottom inset) showing both panels I and II relative to each other. Collections are grouped into regions 1–7 (R01–R07) as indicated by colored subbasins. There are 15 unique collection sites labeled by site abbreviations (Table 1).

Sample collections were comprised of the following overlapping subsets of samples (a–e) and used in various combinations to address our objectives: (a) morphologically‐confirmed ocean‐phase WRL collected at sea by Fisheries and Oceans Canada between 2013 and 2019 from “site 1” in the Georgia Basin (Salish Sea, British Columbia, Canada; *N* = 91), which served as a positive control collection of the anadromous ecotype WRL for testing purposes (Table 1, Figure 1), (b) WBL and WRL voucher specimen (*N* = 39) that were a portion of the confident morphologically identified voucher specimen from Ksi Ts'oohl Ts'ap Creek (Nass River Northern B.C., Canada) that could be used for candidate SNP discovery, (c) broadly distributed specimen with both morphological photo documentation and genetic data for testing genus ID concordance (*N* = 1170), (d) broadly distributed specimen with both morphological photo documentation and genetic data for testing *Lampetra* ecotype ID concordance (*N* = 514), and (e) genotyped individuals lacking morphological ID (*N* = 747) that were combined with all other genotyped individuals for characterizing the spatial distributions of genera and ecotypes across the entire geographic area of our study (Table 1).

**TABLE 1:** Genetic species identification across collections of lampreys.

Region no.	Site	Collection	Dataset	Region	Site	Year	Sample numbers						Proportions				
							*Entosphenus*	*Lampetra*				Total	Genus		*Lampetra*		
								UNK	WBL	Int	WRL		E	L	WBL	Int	WRL
01	01	1	(91a, 91c, 90d)	Georgia Basin	GEORGS	2013–2019	0	1	0	0	90	91	0.0%	100.0%	0.0%	0.0%	100.0%
02	02	2	(39b, 248c, 154d, 5e)	Northern B.C.	ZOLZAP	2019	86	10	79	11	67	253	34.0%	66.0%	50.3%	7.0%	42.7%
02	02	3	(99e)	Northern B.C.	ZOLZAP	2020	43		19	5	32	99	43.4%	56.6%	33.9%	8.9%	57.1%
03	03	4	(11e)	WA coast	BINGHA	2019	1	1	9			11	9.1%	90.9%	100.0%	0.0%	0.0%
03	04	5	(231e)	WA coast	NEWAUK	2019	214		16		1	231	92.6%	7.4%	94.1%	0.0%	5.9%
04	05	6	(121c, 53d, 12e)	Estuarine LC	GRAYSR	2019	77		56			133	57.9%	42.1%	100.0%	0.0%	0.0%
04	05	7	(98c, 29d, 34e)	Estuarine LC	GRAYSR	2020	93		39			132	70.5%	29.5%	100.0%	0.0%	0.0%
04	06	8	(90c, 46d, 19e)	Estuarine LC	CZYJSN	2019	51		58			109	46.8%	53.2%	100.0%	0.0%	0.0%
04	06	9	(25c, 16d, 1e)	Estuarine LC	CZYJSN	2020	9		17			26	34.6%	65.4%	100.0%	0.0%	0.0%
04	07	10	(9c, 4d, 4e)	Estuarine LC	SKAMOK	2019	5		8			13	38.5%	61.5%	100.0%	0.0%	0.0%
05	08	11	(48c, 28d)	Mid LC	MILLCR	2019	20		27	1		48	41.7%	58.3%	96.4%	3.6%	0.0%
05	08	12	(3c, 1d, 82e)	Mid LC	MILLCR	2020	13	1	71			85	15.3%	84.7%	100.0%	0.0%	0.0%
05	09	13	(124c, 41d, 2e)	Mid LC	ABNTHY	2019	84		42			126	66.7%	33.3%	100.0%	0.0%	0.0%
05	09	14	(1c, 65e)	Mid LC	ABNTHY	2020	15	1	45	4	1	66	22.7%	77.3%	90.0%	8.0%	2.0%
05	10	15	(81c, 19d)	Mid LC	GERMAN	2019	62		19			81	76.5%	23.5%	100.0%	0.0%	0.0%
05	10	16	(6c, 95e)	Mid LC	GERMAN	2020	44		56	1		101	43.6%	56.4%	98.2%	1.8%	0.0%
06	11	17	(13c)	Upper LC	KALAMA	2019	13					13	100.0%	0.0%	—	—	—
06	12	18	(129c, 6d, 2e)	Upper LC	COWLIT	2019	125		5		1	131	95.4%	4.6%	83.3%	0.0%	16.7%
06	12	19	(50c, 1d, 18e)	Upper LC	COWLIT	2020	61		7			68	89.7%	10.3%	100.0%	0.0%	0.0%
07	13	20	(12c, 6d, 3e)	Mid C	HAMILT	2019	9		6			15	60.0%	40.0%	100.0%	0.0%	0.0%
07	14	21	(21e)	Mid C	LWINDR	2019	12		9			21	57.1%	42.9%	100.0%	0.0%	0.0%
07	15	22	(17e)	Mid C	BIGWHS	2017	1		16			17	5.9%	94.1%	100.0%	0.0%	0.0%
07	15	23	(22e)	Mid C	BIGWHS	2018		1	21			22	0.0%	100.0%	100.0%	0.0%	0.0%
07	15	24	(21c, 20d, 4e)	Mid C	BIGWHS	2019	1		24			25	4.0%	96.0%	100.0%	0.0%	0.0%
Total			(91a, 39b, 1170c, 514d, 747e)				1039	15	649	22	192	1917					

*Note:* “Dataset” indicates the number of individuals in each of the following overlapping subsamples (a–e) used to address multiple objectives: (a) WRL positive control, (b) voucher specimen from Ksi Ts'oohl Ts'ap Creek used for candidate SNP discovery, (c) Specimen with both morphological photo documentation and genetic data for testing genus ID concordance, (d) Specimen with both morphological photo documentation and genetic data for testing *Lampetra* ecotype ID concordance, and (e) individuals with only genetic data. A genetic ID 5‐SNP assay was used to identify *Lampetra* ecotypes: WBL, Intermediate (Int), or WRL. Unknown “UNK” were individuals that could be genetically classified into *Lampetra* genus but not further into ecotypes due to missing genotypic data.

The grand total of unique individuals (*N* = 1917, Table 1) was constructed into 24 collections that represented 15 unique geographical sites and multiple collection years (Table 1). Most of the 15 sites were comprised of unknown proportions of one or more lamprey taxon (“mixed”) and included consecutive years 2019 and 2020. Site 2 was from Ksi Ts'oohl Ts'ap Creek where the Nisga'a Nation Fisheries and Wildlife Department operates a weir for juvenile salmonid monitoring. Additionally, we collected the rest of the dataset (*N* = 1474) as mixed lampreys from 13 tributaries (sites 3–15) of the lower Columbia River (ranging from Grays River near the Columbia River estuary upstream to the White Salmon River in the Columbia Gorge) and SW Washington coast (Chehalis River) primarily in 2019 and 2020. Washington Department of Fish and Wildlife (WDFW) and the U.S. Geological Survey (USGS) operate rotary screw traps on these tributaries to monitor juvenile salmonid outmigration from approximately March through June.

For mixed lamprey trap collections, field crews enumerated all captured lamprey; a sub‐portion had life stage and total length recorded and a tissue sample collected for subsequent genetic species ID. From a subset of tissue‐sampled individuals, photos were taken of key morphological features including whole body, dentition, and tail pigmentation. Photos were used for subsequent ID based on morphological characteristics (Goodman et al. 2009; Lampman 2018). Subsampling of lampreys (tissue samples and photo documentation) occurred as time and resources allowed at each screw trap site. In general, subsampling occurred on a representative portion of the lamprey catch each day and continued throughout the trap operation period.

### 2.2 Morphological ID of Three Focal Lampreys

Morphological IDs of larvae, juveniles, and adults were subsequently made by examining photographic documentation of specimens collected by screw trap operators. The morphological IDs were independently conducted by two lamprey biologists (R. Lampman and G. Silver) each with more than 10 years of experience in lamprey ID. Their decision process when evaluating morphological features to make a conclusive ID was captured in the form of a dichotomous key (Figure S1). Larvae were identified to genus (*Entosphenus* [Pacific Lamprey] vs. *Lampetra* [WBL or WRL, which are morphologically identical]) according to caudal pigmentation patterns (Goodman et al. 2009; Lampman 2018). *Entosphenus* has a light band with an arrow or spade shape that borders the caudal ridge compared to an absence of this band on *Lampetra*; smaller specimens (generally < 100 mm) of *Lampetra* may have a light band, but the arrow/spade shape (i.e., widening of the light band) is absent (Figure S1). Morphological ID was attempted only on larvae greater than about 60 mm total length because those caudal pigmentation patterns are generally not distinguishable on smaller larvae (< 50 mm). Juvenile and adult lamprey were identified to taxon (Pacific Lamprey, WBL, or WRL) according to caudal pigmentation, dentition, and body size (Figure S1). Dentition patterns show three supraoral cusps on *Entosphenus* and two on *Lampetra*. Dentition is further used to distinguish between WBL and WRL because the latter tends to have sharper teeth as adults (and juvenile). Body sizes of adults overlap to some extent but are generally discernible, with the largest sizes being Pacific Lamprey (330–840 mm), followed by WRL (200–330 mm), and then WBL (100–200 mm).

### 2.3 DNA Analysis

Tissue samples were snipped from dorsal or caudal fins by field crews, then preserved and stored dry on Whatman paper or in coin envelopes with paper inserts. Samples were later shipped to the CRITFC Hagerman Genetics Lab (Hagerman, ID) where they were processed and genotyped. DNA was extracted using the same methods described by Hess et al. (2013) before applying protocols for genotyping‐in‐thousands by sequencing (GT‐seq) custom amplicon methods (Campbell et al. 2015) on an Illumina sequencer. The existing primers (SNP GT‐seq assays: LampSD_478 and LampSD_700; Hess et al. 2015) to determine genus (*Entosphenus* vs. *Lampetra*) were published by Hess et al. (2020) and are available in the Dryad repository (https://doi.org/10.5061/dryad.hx3ffbgc2).

### 2.4 Genetic ID of Lamprey Genera

We determined genus (*Entosphenus* vs. *Lampetra*) using two redundant SNPs (LampSD_478 and LampSD_700) by using a conditional formula that classified genotypes as *Lampetra* if either LampSD_478 was “AA”, or LampSD_700 was “TT”; *Entosphenus* was classified if LampSD_478 was “GG”, or LampSD_700 was “GG”; and “Hybrid” was classified if either LampSD_478 was heterozygous “GA” or LampSD_700 was heterozygous “GT”. Hybrid genotypes have not been observed to date for these diagnostic loci, but it could occur under the conditions described previously (Arakawa et al. 2021).

### 2.5 A Novel SNP Marker for Genetic ID of *Lampetra* Ecotypes: WBL and WRL

We used a reference whole genome assembly of a WBL specimen from the Western North American genus *Lampetra* (GenBank Accession Number: JARYGF000000000, JASBGX000000000) to identify genomic positions of SNPs that were differentiated between the two morphotypes, WBL and WRL (sample b, *N* = 39). The whole genome sequencing methods are detailed in our Supporting Information. We also conducted low coverage WGS (individually barcoded Pool‐seq; Horn et al. 2020) of WBL and WRL voucher specimens (sample b; WBL *N* = 24, WRL *N* = 15; Table 2, Figure S2) to identify SNPs of high *F*_ST_ (i.e., *F*_ST_ values > 99.5% across markers) by conducting pairwise comparisons using PPanalyze (module in POOLPARTY bioinformatic pipeline, Micheletti and Narum 2018; Supporting Information). To reduce noise and more easily resolve divergent regions, a sliding window *F*_ST_ summary was generated using windows of 10 kb, a step size of 5 kb, and minimum coverage of four individuals per site; the Manhattan plot was created with the PPmanhat module in POOLPARTY using the first 100 largest scaffolds that potentially correspond to 100 pairs of chromosomes (lampreys range from 78 to 178 chromosomes, Caputo et al. 2011).

**TABLE 2:** Voucher details and field, morphological, and genetic ID of *Lampetra* ecotypes.

ID	Life stage	Length (mm)	Sex	Dichotomous key	MorphID	5‐SNP assay		Morph. vs. Gen. ID
						%WRL	Ecotype	Concordance
LayZolzap19Adult‐S0001	Adult	175	—	1‐YES‐2A‐NO‐3‐NO‐4B‐NO‐5‐NO	WBL	25.0%	WBL	Match
LayZolzap19Adult‐S0010	Adult	155	—	1‐YES‐2A‐NO‐3‐NO‐4B‐NO‐5‐?	WBL	20.0%	WBL	Match
LayZolzap19Adult‐S0011	Adult	150	F	1‐YES‐2A‐NO‐3‐NO‐4B‐NO‐5‐NO	WBL	10.0%	WBL	Match
LayZolzap19Adult‐S0012	Adult	157	M	1‐YES‐2A‐NO‐3‐NO‐4B‐NO‐5‐NO	WBL	33.3%	WBL	Match
LayZolzap19Adult‐S0014	Adult	151	M	1‐YES‐2A‐NO‐3‐NO‐4B‐NO‐5‐NO	WBL	12.5%	WBL	Match
LayZolzap19Adult‐S0015	Adult	154	M	1‐YES‐2A‐NO‐3‐NO‐4B‐NO‐5‐NO	WBL	12.5%	WBL	Match
LayZolzap19Adult‐S0016	Adult	160	—	1‐YES‐2A‐NO‐3‐NO‐4B‐NO‐5‐?	WBL	50.0%	Intermediate	—
LayZolzap19Adult‐S0017	Adult	150	M	1‐YES‐2A‐NO‐3‐NO‐4B‐NO‐5‐NO	WBL	30.0%	WBL	Match
LayZolzap19Adult‐S0018	Adult	152	M	1‐YES‐2A‐NO‐3‐NO‐4B‐NO‐5‐NO	WBL	30.0%	WBL	Match
LayZolzap19Adult‐S0021	Adult	150	—	1‐YES‐2A‐NO‐3‐NO‐4B‐NO‐5‐?	WBL	70.0%	WRL	FALSE
LriZolzap19Adult‐B0001	Adult	136	F	1‐YES‐2A‐NO‐3‐NO‐4B‐NO‐5‐NO	WBL	20.0%	WBL	Match
LriZolzap19Adult‐B0002	Adult	145	—	1‐YES‐2A‐NO‐3‐NO‐4B‐NO‐5‐NO	WBL	12.5%	WBL	Match
LriZolzap19Adult‐B0003	Adult	166	F	1‐YES‐2A‐NO‐3‐NO‐4B‐NO‐5‐NO	WBL	10.0%	WBL	Match
LriZolzap19Adult‐B0005	Adult	177	M	1‐YES‐2A‐NO‐3‐NO‐4B‐NO‐5‐NO	WBL	20.0%	WBL	Match
LriZolzap19Adult‐B0006	Adult	134	F	1‐YES‐2A‐NO‐3‐NO‐4B‐NO‐5‐NO	WBL	0.0%	WBL	Match
LriZolzap19Adult‐B0010	Adult	167	F	1‐YES‐2A‐NO‐3‐NO‐4B‐NO‐5‐NO	WBL	20.0%	WBL	Match
LriZolzap19Adult‐B0011	Adult	183	F	1‐YES‐2A‐NO‐3‐NO‐4B‐NO‐5‐NO	WBL	—	—	—
LriZolzap19Adult‐B0014	Adult	151	F	1‐YES‐2A‐NO‐3‐NO‐4B‐NO‐5‐NO	WBL	—	—	—
LriZolzap19Adult‐B0016	Adult	144	M	1‐YES‐2A‐NO‐3‐NO‐4B‐NO‐5‐NO	WBL	20.0%	WBL	Match
LriZolzap19Adult‐B0017	Adult	152	F	1‐YES‐2A‐NO‐3‐NO‐4B‐NO‐5‐NO	WBL	—	—	—
LriZolzap19Adult‐B0020	Adult	175	M	1‐YES‐2A‐NO‐3‐NO‐4B‐NO‐5‐NO	WBL	33.3%	WBL	Match
LriZolzap19Adult‐B0024	Adult	153	M	1‐YES‐2A‐NO‐3‐NO‐4B‐NO‐5‐NO	WBL	10.0%	WBL	Match
LriZolzap19Adult‐B0027	Adult	122	F	1‐YES‐2A‐NO‐3‐NO‐4B‐NO‐5‐NO	WBL	0.0%	WBL	Match
LriZolzap19Adult‐B0029	Adult	149	M	1‐YES‐2A‐NO‐3‐NO‐4B‐NO‐5‐NO	WBL	37.5%	WBL	Match
subtotal WBL Match					24			19
subtotal WBL Mismatch					—			1
subtotal WBL Intermediate								1
subtotal WBL Unknown					—			4
WBL ID concordance					19 match/(19 match + 1 mismatch + 1 intermediate) = 90.5%			
LayZolzap19Adult‐S0002	Juvenile	156	—	1‐YES‐2A‐NO‐3‐NO‐4B‐NO‐5‐YES	WRL	100.0%	WRL	Match
LayZolzap19Adult‐S0003	Juvenile	183	—	1‐YES‐2A‐NO‐3‐NO‐4B‐NO‐5‐YES	WRL	80.0%	WRL	Match
LayZolzap19Adult‐S0004	Juvenile	161	—	1‐YES‐2A‐NO‐3‐NO‐4B‐NO‐5‐YES	WRL	100.0%	WRL	Match
LayZolzap19Adult‐S0005	Juvenile	149	—	1‐YES‐2A‐NO‐3‐NO‐4B‐NO‐5‐YES	WRL	50.0%	Intermediate	—
LayZolzap19Adult‐S0006	Juvenile	147	—	1‐YES‐2A‐NO‐3‐NO‐4B‐NO‐5‐YES	WRL	100.0%	WRL	Match
LayZolzap19Adult‐S0009	Juvenile	157	—	1‐YES‐2A‐NO‐3‐NO‐4B‐NO‐5‐YES	WRL	80.0%	WRL	Match
LayZolzap19Adult‐S0013	Juvenile	158	—	1‐YES‐2A‐NO‐3‐NO‐4B‐NO‐5‐YES	WRL	100.0%	WRL	Match
LayZolzap19Adult‐S0019	Juvenile	166	—	1‐YES‐2A‐NO‐3‐NO‐4B‐NO‐5‐YES	WRL	30.0%	WBL	FALSE
LayZolzap19Adult‐S0020	Juvenile	171	—	1‐YES‐2A‐NO‐3‐NO‐4B‐NO‐5‐YES	WRL	—	—	—
LriZolzap19Adult‐B0012	Adult	196	—	1‐YES‐2A‐NO‐3‐YES‐4A‐NO	WRL	50.0%	Intermediate	—
LriZolzap19Adult‐B0018	Adult	220	M	1‐YES‐2A‐NO‐3‐YES‐4A‐NO	WRL	—	—	—
LriZolzap19Adult‐B0021	Adult	217	F	1‐YES‐2A‐NO‐3‐YES‐4A‐NO	WRL	—	—	—
LriZolzap19Adult‐B0022	Adult	192	F	1‐YES‐2A‐NO‐3‐YES‐4A‐NO	WRL	—	—	—
LriZolzap19Adult‐B0025	Adult	205	M	1‐YES‐2A‐NO‐3‐YES‐4A‐NO	WRL	100.0%	WRL	Match
LriZolzap19Adult‐B0028	Adult	200	—	1‐YES‐2A‐NO‐3‐YES‐4A‐NO	WRL	100.0%	WRL	Match
Subtotal WRL Match					15			8
Subtotal WRL Mismatch					—			1
Subtotal WRL intermediate								2
Subtotal WRL Unknown					—			6
WRL ID concordance					8 match/(8 match + 1 mismatch + 2 intermediate) = 72.7%			
Total ecotype ID concordance					27 match/(27 match + 2 mismatch + 3 intermediate) = 84.4%			

*Note:* Sex was determined visually from photos. Morphological ID placed fish into WBL and WRL categories based on experienced training to recognize morphological distinctions of these taxa, which followed the stated path through the dichotomous key (Figure S1). Morphological ID groups were used to conduct genome‐wide association study to identify candidate SNPs. The 5‐SNP candidate assay was developed from the GWAS and used to classify them into ecotypes based on the percentage of WRL alleles: WBL (< 50% WRL alleles), Intermediate (50% WRL alleles), and WRL (> 50% WRL alleles); and missing genotypes (—). Concordance is the percent of matching IDs out of all specimens with matching (“match”), mismatching (“false”), or “Intermediate” genotypes when comparing morphological and genetic IDs for ecotype. Three morphological IDs were placed into question following reinspection while comparing genetic and morphological IDs because the specimens appeared to have both features, which led to an ambiguous answer in step 5 of the dichotomous key.

By comparing morphotypes WBL and WRL in “sample b”, we targeted chromosomes containing intervals of high *F*_ST_ to develop SNP assays using primer design for GT‐seq applications following previous methods (e.g., Hess et al. 2015); specifically, we targeted approximately 50 SNPs to have the highest *F*_ST_ (> 0.5) and proportional representation within the high *F*_ST_ intervals on chromosomes without regard for a particular spacing (SNPs were on average790 kbp apart, range 106 bp–5 Mbp). Primers of 20–25 bp length were designed around amplicons of a maximum of 150 bp length (with the internal SNP within the first 75 bp) to provide optimal quality in reads for sequencing by genotyping in thousands on an Illumina using the Python primer design pipeline (https://github.com/StevenMicheletti).

The same 39 specimens (sample b) used for WGS were re‐examined using genotypes from the GT‐seq assays, and the top five SNP assays were chosen to screen all study collections of *Lampetra* based on the highest *F*_ST_ (i.e., comparisons of WBL and WRL). Finally, we tested the 5‐SNP candidate assay using ocean‐phase WRL from the Georgia Basin (sample a; site 1) as a positive control to quantify the association of alleles at the loci with the ecotypes.

Given the Western North American *Lampetra* ecotypes represent suites of life history traits that are similar to those traits distinguishing European *Lampetra* ecotypes ( *L. planeri* and *L. fluviatilis* , Souissi et al. 2022), we expected that similar genomic regions may be associated with both pairs of ecotypes despite the phylogenetic separation between Western North American *Lampetra* and European *Lampetra*. To test this hypothesis, we aligned candidate genomic regions that we observed with high *F*_ST_ in *Lampetra* ecotypes WBL and WRL to the improved and annotated sea lamprey genome assembly (Timoshevskaya et al. 2023). These methods were repeated using the published candidate regions from European *Lampetra* ecotypes (Souissi et al. 2022). We performed whole genome alignments using minimap2 (v2.17) using alignment parameters tuned for moderately divergent genomes (‐cx asm20). Alignments were filtered to retain those with mapping quality 50 to reduce signal from abundant repetitive elements.

### 2.6 Concordance of Morphological and Genetic IDs of Lamprey Genera and *Lampetra* Ecotypes

Individuals that had both a morphological ID and a genetic ID were compared for matching ID at the genus level (*Entosphenus* and *Lampetra*) and within the genus *Lampetra* at the ecotypic level (WBL and WRL). For genus comparisons, we calculated concordance as the percentage of all fish with a genetic ID of *Entosphenus*, *Lampetra*, or total (*Entosphenus* + *Lampetra*) that had a matching morphological ID out of the total number of fish that had a definitive genetic and morphological ID. We ignored any specimen that did not have a definitive genus ID for both genetic and morphological ID methods. For specimens that were identified as *Lampetra*, concordance was calculated as the percent of all fish that were genetically identified as WBL, WRL, or total (WBL + WRL) that had matching ID using morphology for each of the unique collection sites. For the purposes of calculating concordance rates of *Lampetra* ecotypes, we first ignored “nondefinitive” individuals that were either genetically determined to be “intermediate” (i.e., heterozygous genotypes) or individuals (mostly larval) that were morphologically determined as “unknown” ecotype. We then showed how concordance rate is affected when each intermediate genetic ID of *Lampetra* ecotypes was tallied as part of the total nonmatching IDs for all cases where it had a corresponding morphological ID with a definitive ecotype. Finally, we showed what proportion of nondefinitive morphological IDs had a corresponding definitive genetic ID for ecotypes.

### 2.7 Correlation of Genetic Species Composition With Geography and Time (Collection Years) and Population Structure

#### 2.7.1 Composition of Lamprey Genera: *Lampetra* vs. *Entosphenus*

Using only successfully genotyped specimens (samples c and e), we characterized the relative proportion of the two genera *Lampetra* and *Entosphenus* across streams and years and generated 99% confidence intervals via 10,000 bootstraps. Bootstraps were estimated with the R function fishComptools (https://github.com/delomast/fishCompTools). The 15 unique collection sites (24 total collections including temporal replicates, Table 1) were grouped into the following seven different regions: (1) Georgia basin (site 01), (2) Northern B.C. (site 02), (3) WA coast (sites 03 and 04), (4) estuarine lower Columbia (sites 05, 06, and 07), (5) mid lower Columbia (sites 08, 09, and 10), (6) upper lower Columbia (sites 11 and 12), and (7) mid‐Columbia (sites 13, 14 and 15). Among the 14 unique sites (excluding the Georgia Basin reference collection), there were 8 sites with temporal replication (i.e., sampled in more than 1 year, Table 1). We tested the following two null hypotheses: (i) composition of lamprey genera was spatially stable (i.e., overlapping confidence intervals for proportions of *Entosphenus* within each region) and (ii) temporally stable (i.e., overlapping confidence intervals for proportions of *Entosphenus* across collection years at each site).

#### 2.7.2 Composition of *Lampetra* Ecotypes: WBL vs. WRL

For all specimens that were genetically assigned to the genus *Lampetra* (samples c and the *Lampetra* portion of e), we employed the novel 5‐SNP candidate genetic assay to identify the relative proportions of ecotypes WBL and WRL. For this assay, each allele for each of the five bi‐allelic candidate SNPs was classified as either WRL or WBL based on which ecotype had higher relative frequencies among the voucher specimen (sample b). The percent WRL was calculated as the total number of WRL alleles that were found across these five SNPs in an individual's genotype divided by the total number of alleles that successfully genotyped (10 alleles when all five SNPs successfully genotyped). A threshold of ≥ 3 successfully genotyped SNPs was set before the calculation of “percent WRL” was performed with this genetic ID at this 5‐SNP assay and at least 1 SNP of the 2 SNPs on chromosome 2 was required to successfully genotype for every individual (this ensured the most diagnostic chromosome was represented for every genetic ID calculation). Genetic ID of WRL and WBL ecotypes was classified as having greater than or less than 50% WRL alleles, respectively. Individuals with 50% WRL alleles were classified as “intermediate” genetic IDs. Despite using this basic classification of WRL and WBL ecotypes for most of the genetic ID analyses, we also explored nuance in the potential for admixture between ecotypes by plotting individual‐level composition of WRL alleles across all collections. We used known ocean‐phase collections of WRL in the Strait of Georgia as a positive control for this diagnostic marker. We tested the same two null hypotheses tested for the compositions of lamprey genera: i.e., are compositions of *Lampetra* ecotypes spatially and temporally stable? In addition, we examined whether distance (river kilometers, rkm) from the mouth of the Columbia River was correlated with frequencies of the WRL alleles. Our expectation was that WRL alleles would be observed in higher frequencies closer to the ocean given that this ecotype is anadromous and lacking the ability to overcome passage barriers as easily as Pacific Lamprey and other anadromous fishes in the Columbia River. Correlations were evaluated with a Mantel test using the ade4 package in R, and *p*‐values were generated with 9999 permutations.

#### 2.7.3 Population Genetic Structure Between *Lampetra* Ecotypes

We used the low‐per‐individual‐coverage WGS data from the voucher specimen (sample b) to estimate how many populations were represented in Ksi Ts'oohl Ts'ap Creek. We generated individual genotypes for a small portion of SNPs that had sufficient individual read depth (≥ 5 reads per individual) and high minor allele frequency across ecotypes (MAF > 0.1). The 835 individual genotypes of SNPs that were successfully estimated for > 70% of all individuals for the 39 voucher specimens (see methods in Supporting Information) were combined with the 47 GT‐seq SNP genotypes and then analyzed with Discriminant Analysis of Principal Components (DAPC) to estimate the number of *k* clusters these individuals represented using the adegenet package in R (Jombart et al. 2010). The DAPC was conducted by running from 1 to 6 clusters while retaining 300 principal components of the genetic data (882 total SNP genotypes). The percent variation explained in the data was plotted against the number of principal components to determine the number of PCs that could explain > 90% of the variance in the data. The number of clusters (populations) represented in our dataset was estimated using Bayesian Information Criterion (BIC) to select the number of *k* clusters with the lowest BIC. We repeated the DAPC methods on a subset of the 835 low coverage WGS genotypes that utilized only the loci that were spaced > 1000 bp apart on each scaffold; this trimming step (*N* = 303 loci) helped ensure that our interpretations of results avoided bias from over‐representation of any duplicated regions of the genome.

Finally, we used a method to identify *F*_ST_ outliers to confirm whether the 47 GT‐seq SNPs had extreme *F*_ST_ values relative to other random SNPs from the genome (835 individual genotypes estimated from the WGS data above). We tested this dataset for *F*_ST_ outliers using the program BayeScan v2.1 (Foll and Gaggiotti 2008) with default settings of a burn in of 50,000 and 100,000 total iterations. Extreme *F*_ST_ values were determined using posterior probabilities of 0.7 and 0.9 to determine SNPs that had substantial and strong evidence of being outliers (Foll and Gaggiotti 2008).

## 3 Results

### 3.1 Morphological ID

Of the total of 1917 mixed lampreys in our dataset that were successfully genotyped (minimally genera ID markers could be scored), 1079 lampreys were collected with morphological IDs that were accompanied by photographic documentation by field crews (sample c, Table 1), and there were 91 fish used as a positive control sample with confirmed morphological IDs of genus *Lampetra* and ecotype WRL (sample a, Table 1). In total, there were 1170 samples that had both morphological and genetic ID that could be used to test concordance for genera Pacific Lamprey (*Entosphenus*) and *Lampetra*. Within these samples, there were 514 fish (sample d, Table 1) that were both morphologically and genetically confirmed as *Lampetra*; the morphological IDs of these fish were 106 WRL, 175 WBL, and 233 unknown ecotypes (larvae). Ninety of the 106 morphologically identified WRL were the positive control from the Georgia Basin, and of the remaining WRL morphological IDs, there were 15 from Northern B.C. (ZOLZAP, Figure 1), and a single WRL adult was found in the Lower Columbia River (site 12, region 06; Figure S4).

### 3.2 Genetic ID of Lamprey Genera

Of the total 1917 mixed lampreys that were successfully genotyped, 1039 were *Entosphenus* and 878 were *Lampetra* across all 15 collection sites. There were no hybrid genotypes detected for either diagnostic marker. Most of these 1917 (99.6%) were genetically identified based on successful genotypes at both redundant genera ID markers (LampSD_478 and LampSD_700); however, two of the *Entosphenus* specimens and five of the *Lampetra* specimens had only the LampSD_478 genotype, and one of the *Lampetra* specimens had only the LampSD_700 genotype.

### 3.3 Multi‐SNP Marker to Differentiate *Lampetra* Ecotypes: WBL and WRL

**FIGURE 2:**
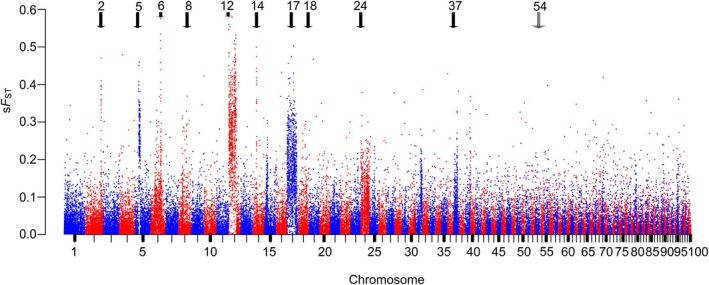
Sliding window *F*_ST_ Manhattan plot for the first 100 scaffolds (chromosomes) of the *Lampetra richardsoni* genome assembly comparing putative WRL with WBL specimen from Ksi Ts'oohl Ts'ap Creek. High *F*_ST_ markers on scaffolds 2, 5, 6, 8, 12, 14, 17, 18, 24, 37 were developed into GT‐seq assays to test (black arrows). Every 5th interval chromosome is labeled and colored by odd (blue) and even (red) chromosome numbers. For reference, the chromosome 54 with homology to the candidate locus identified by Souissi et al. (2022) to distinguish European *Lampetra* ecotypes is indicated (gray arrow) but was not indicated as a candidate chromosome for Western North American *Lampetra* ecotypes.

The analysis of WGS data identified 3,151,893 SNPs on the top 100 scaffolds (referred here as chromosomes) that passed quality filters and 9529 SNPs that we considered as “high” *F*_ST_ (*F*_ST_ > 0.500) because they had higher values than 99.7% of all the SNPs identified. Eight of the 100 chromosomes (chromosomes 2, 5, 6, 12, 14, 17, 24, and 37) each contained more than 1% of all high *F*_ST_ SNPs, and together those eight chromosomes contained 81% of all high *F*_ST_ SNPs across the 100 scaffolds (Table S1). We developed 43 SNP assays on all eight of these high‐*F*_ST_ chromosomes and an additional two assays for high *F*_ST_ SNPs on each of chromosomes 8 and 18 for a total of 47 candidate SNP assays. The 10 chromosomes are indicated on the sliding window *F*_ST_ Manhattan plot, which shows that each of these chromosomes contains relatively large regions of high *F*_ST_ (Figure 2, Table S2). The 47 candidate SNPs were estimated from the genome resequencing data to have an average *F*_ST_ of 0.691 (range 0.506–0.933, Table S3).

The 47 candidate SNP assays were genotyped using GT‐seq on the same 39 specimens for increased accuracy of genotype frequencies and estimates of *F*_ST_. These new genotypes resulted in lower average *F*_ST_ across all loci (average *F*_ST_ of 0.249, range 0.012–0.583; Table S3). Based on the GT‐seq genotypes of the 47 candidate SNPs, there were only two SNPs with *F*_ST_ > 0.500, and both were on chromosome 2 (Lri2P16333005 and Lri2P16367064) and located approximately 34 kbp apart. The next three highest *F*_ST_ values were SNPs on chromosome 5 (Lri5P4640927: *F*_ST_ = 0.464), 12 (Lri12P6060911: *F*_ST_ = 0.480), and 14 (Lri14P4079387: *F*_ST_ = 0.454). These five candidate SNPs were chosen to genotype using GT‐seq assays all specimens in the dataset. We first validated the ability of these SNPs to ID the ecotypes of the 39 specimens used in the WGS dataset by classifying them into ecotypes based on the percent of WRL alleles at this 5‐SNP candidate assay and comparing to their individual morphological ID (below).

For the 39 specimens used for WGS analysis, the 5‐SNP GT‐seq assay successfully genotyped 11 of the 15 individuals that had WRL morphological ID, and of those 11 genotyped, eight specimens were genetically classified as WRL (73%, Table 2). For the 24 WBL morphological ID specimens, there were 21 that successfully genotyped, and 19 of those were genetically classified as WBL (90%, Table 2). For the five different SNPs, the A, G, A, T, and A alleles were determined to be the “WRL” allele, and the T, A, T, C, and C alleles were determined to be the “WBL” allele for Lri2P16333005, Lri2P16367064, Lri5P4640927, Lri12P6060911, and Lri14P4079387 SNPs, respectively. There was some portion of both the WRL (2, 18%) and WBL morphological ID specimens (1, 5%) that were heterozygous across all five SNPs and were classified as “intermediate” for this genetic ID. There was a single individual in each of the morphological IDs of WBL (5%) and WRL (9%) that was misclassified according to the genetic ID. Finally, the “positive control” collection of ocean‐phase WRL specimens was genetically ID'd as 100% WRL (Table 1). These 5 SNPs together provided greater total assignment accuracy (84%; 27 matching IDs out of 32 successful genotypes) than any of the candidate SNPs alone (range of total assignment accuracies 54%–78% across separately evaluated candidate SNPs, Table S6). Further, the 5‐SNP assay resulted in 100% definitive ID of WRL in the positive control group, whereas any single candidate SNP used separately resulted in definitive WRL IDs between 81% and 100% due to some of the candidate SNPs having heterozygotes.

We found that the 10 genomic regions (i.e., chromosomes 2, 5, 6, 8, 12, 14, 17, 18, 24, and 37) identified in this study are orthologous to sea lamprey chromosomes (1, 6, 10, 2, 34, 31, 25, 2, 8, and 15, respectively, Table S4). One of the five candidate SNPs that we chose is located at position 16,367,064 on *Lampetra richardsoni* chromosome 2 and 16,531,928 on sea lamprey chromosome 1 within a few kilobases downstream of a gene known as synaptic vesicle glycoprotein 2C, SV2C (Figure S3). The original version of the *Lampetra richardsoni* genome assembly was found to have an incomplete assembly of chromosome 2. The SV2C gene was distributed across LPT_scaf_2 (original chromosome 2) and multiple shorter scaffolds (LPT_scaf_8586, LPT_scaf_493, and LPT_scaf_5926). Reassembly of the *Lampetra richardsoni* chromosome 2 and re‐alignment of the Pool‐seq data to this reassembled chromosome 2 reference allowed a more accurate estimation of the region of divergence centered around the annotated gene SV2C and relative to the locations of two candidate SNPs that were developed (SNPs Lri2P16333005 and Lri2P16367064; Figure S3; Supporting Information: Methods). The regions of divergence localized on *Lampetra* chromosomes 5, 12, and 14 were broader (range 2.1 kbp–7.7 Mbp) compared to the region on chromosome 2 (1.5 kbp) and annotations from the sea lamprey genome showed those regions contained multiple genes. The nearest genes to the SNPs that were developed for the 5‐SNP assay were NIN, ACVR2, and ESR1 for *Lampetra* chromosomes 5, 12, and 14, respectively (Table S4).

The Souissi et al. (2022) SNPs were localized to regions that aligned only to chromosome 54 in *Lampetra richardsoni* and sea lamprey chromosome 64 in the range of 3,171,553–5,262,594 bp (Table S5). This genomic region was not remarkable in terms of presence of high *F*_ST_ SNPs for the *Lampetra* ecotypes in our study (Figure 2).

### 3.4 Concordance of Morphological and Genetic ID of Lamprey Genera and *Lampetra* Ecotypes

A total of 1917 lamprey tissue samples were successfully genotyped to determine genus ID (*Entosphenus* vs. *Lampetra*; Table 1). This genotyped group included sample “c” which was used for concordance tests between morphological and genetic ID: 1170 lampreys photographed for morphological ID from 11 collection sites in Western and Southwestern Washington and Northern B.C. and the positive control *Lampetra* from Georgia Basin (Tables 1 and 3). Concordance between morphological and genetic ID methods was high; 634 of the 645 total identified *Entosphenus* matched their genetic and morphological IDs (98.3% concordance, Table 3). Eleven specimens had genetic and morphological ID disagreements, all of which were larvae that were morphologically identified as *Lampetra* yet genotyped as *Entosphenus*; poor image quality (e.g., darkness, blurriness, incomplete tail imaging) significantly hampered the morphological ID process in almost all these cases (10 of 11 instances). There were 525 of 536 of the total identified *Lampetra* that matched genetic and morphological IDs (97.9% concordance, Table 3). In total, 1159 of all the genera IDs matched between genetic and morphological IDs (99.1% concordance).

**TABLE 3:** Concordance between morphological and genetic ID methods for lamprey genera.

Region no.	Region	Site	Genetic ID	Morphological ID		Total	Concordance
				*Entosphenus*	*Lampetra*		
01	Georgia Basin	01	*Entosphenus*				—
			*Lampetra*		91	91	100.0%
		01 Subtotal			91	91	100.0%
02	Northern B.C.	02	*Entosphenus*	84		84	100.0%
			*Lampetra*		164	164	100.0%
		02 Subtotal		84	164	248	100.0%
04	Estuarine LC	05	*Entosphenus*	135	2	137	98.5%
			*Lampetra*		82	82	97.6%
		05 Subtotal		135	84	219	99.1%
		06	*Entosphenus*	51	2	53	96.2%
			*Lampetra*		62	62	96.9%
		06 Subtotal		51	64	115	98.3%
		07	*Entosphenus*	5		5	100.0%
			*Lampetra*		4	4	100.0%
		07 Subtotal		5	4	9	100.0%
05	Mid LC	08	*Entosphenus*	21	1	22	95.5%
			*Lampetra*		29	29	96.7%
		08 Subtotal		21	30	51	98.0%
		09	*Entosphenus*	84		84	100.0%
			*Lampetra*		41	41	100.0%
		09 Subtotal		84	41	125	100.0%
		10	*Entosphenus*	68		68	100.0%
			*Lampetra*		19	19	100.0%
		10 Subtotal		68	19	87	100.0%
06	Upper LC	11	*Entosphenus*	13		13	100.0%
		11 Subtotal		13		13	100.0%
		12	*Entosphenus*	167	5	172	97.1%
			*Lampetra*		7	7	58.3%
		12 Subtotal		167	12	179	97.2%
07	Mid C	13	*Entosphenus*	6		6	100.0%
			*Lampetra*		6	6	100.0%
		13 Subtotal		6	6	12	100.0%
		15	*Entosphenus*		1	1	0.0%
			*Lampetra*		20	20	95.2%
		15 Subtotal			21	21	95.2%
		Total Site	*Entosphenus*	634	11	645	98.3%
			*Lampetra*	0	525	525	97.9%
		Grand Total		634	536	1170	99.1%

Among the 525 individuals that were confirmed genetically and morphologically as the genus *Lampetra*, we created a subset (sample “d”, *N* = 514, Table 1) to test the concordance of morphological and genetic ID of *Lampetra* ecotypes. This sample “d” included 514 individuals that were successfully genotyped using the candidate 5‐SNP assay (497 individuals successfully genotyped for all 5 SNPs and among remaining 17 individuals no more than 2 of the 5 SNPs failed to genotype) and had morphological ID (Tables 1 and 4). The sample “d” *Lampetra* was collected across 11 collection sites. There were 101 of 105 total identified definitive WRL that matched genetic and morphological definitive IDs (96.2% concordance, Table 4), whereas 171 of 175 identified definitive WBL matched genetic and morphological definitive IDs (97.7%). In total, 272 of all definitive 276 *Lampetra* ecotype IDs (ignoring intermediate genotypes) matched genetic and morphological IDs (98.6%). This concordance rate is only slightly lower (96.8%) if it is calculated as the percent of matching genetic IDs (272 matched IDs) out of the total number of definitive morphological IDs (281, Table 4), which considers intermediate genotypes as nonmatching. There was a total of 233 specimens that could not be compared because the morphology had an unknown ID (i.e., larvae). There were an additional 12 specimens that were intermediate genotypes and so could not be compared for concordance. In total, the unclassified (i.e., nondefinitive) category of specimens (*N* = 245) comprised 2/3 of the *Lampetra* dataset that had both photo and genetic information; however, using genetic information alone would resolve 92% of these into definitive (*N* = 226, 54 WRL and 172 WBL), whereas the morphological ID would only resolve 2% of these into definitive ecotypic ID (*N* = 5, 2 WRL, 3 WBL; Table 4).

**TABLE 4:** Concordance between morphological and genetic ID methods for *Lampetra* ecotypes.

Region#	Region	Site	Genetic ID	Morphological ID			Total	Concordance
				WRL	Unknown	WBL		
01	Georgia Basin	01	WRL	90			90	100.0%
			Intermediate					
			WBL					—
		01 Total		90			90	100.0%
02	Northern B.C.	02	WRL	10	54	1	65	71.4%
			Intermediate	2	7	2	11	—
			WBL	3	55	20	78	83.3%
		02 Total		15	116	23	154	88.2%
04	Estuarine LC	05	WRL	—	—	—	—	—
			Intermediate	—				—
			WBL	—	44	38	82	100.0%
		05 Total		—	44	38	82	100.0%
		06	WRL	—	—	—	—	—
			Intermediate	—	1		1	—
			WBL	—	41	20	61	100.0%
		06 Total		—	42	20	62	100.0%
		07	WRL	—	—	—	—	—
			Intermediate	—	—	—	—	—
			WBL	—	2	2	4	100.0%
		07 Total		—	2	2	4	100.0%
05	Mid LC	08	WRL	—	—	—	—	—
			Intermediate	—	1	4	5	—
			WBL	—	5	19	24	100.0%
		08 Total		—	6	23	29	100.0%
		09	WRL	—	—	—	—	—
			Intermediate	—	—	1	1	—
			WBL	—	2	38	40	100.0%
		09 Total		—	2	39	41	100.0%
		10	WRL	—	—	—	—	—
			Intermediate	—	—	1	1	—
			WBL	—	3	15	18	100.0%
		10 Total		—	3	16	19	100.0%
06	Upper LC	12	WRL	1	—	—	1	100.0%
			Intermediate	—	—	—	—	—
			WBL	—	3	3	6	100.0%
		12 Total		1	3	3	7	100.0%
07	Mid C	13	WRL	—	—	—	—	—
			Intermediate	—	—	—	—	—
			WBL	—	—	6	6	100.0%
		13 Total		—	—	6	6	100.0%
		15	WRL	—	—	—	—	—
			Intermediate	—	—	—	—	—
			WBL	—	15	5	20	100.0%
		15 Total		—	15	5	20	100.0%
		Total site	WRL	101	54	1	156	96.2%
			Intermediate	2	7	3	12	—
			WBL	3	172	171	346	97.7%
		Grand total		106	233	175	514	98.6%

### 3.5 Spatial and Temporal Correlation of Composition of Genetic IDs

#### 3.5.1 Composition of Lamprey Genera: *Lampetra* vs. *Entosphenus*

**FIGURE 3:**
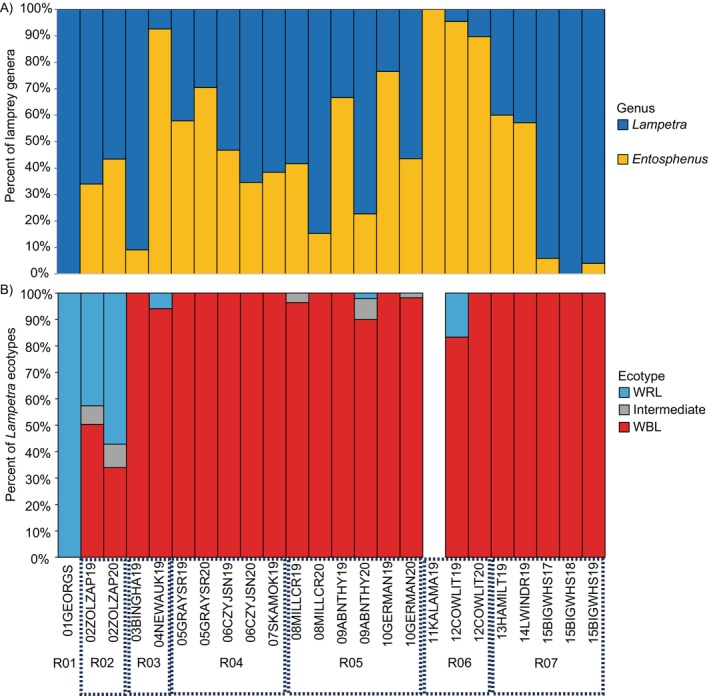
Proportions of (a) lamprey genera (*Entosphenus*, orange; *Lampetra*, blue) and (b) the ecotypes of *Lampetra* based on the five candidate alleles across collections. Dashed boxes indicate geographic grouping of collections into regions. Western River Lamprey “WRL” and Western Brook Lamprey “WBL” are genotypes with > 50% and < 50% of WRL alleles across the five candidate SNPs, respectively; “Intermediate” are genotypes with 50% of WRL alleles across the five candidate SNPs.

*Entosphenus* and *Lampetra* genera were both present in 13 of the 15 collection sites; the Kalama River (*N* = 13, site 11, Table 1) was a tributary site with just one genus, *Entosphenus* (Figure 3a); the Georgia Basin (site 1, Figure 3a) was also represented by a single genus, *Lampetra*, but this was intentional as a WRL positive control. All regional groupings of tributary sites contained both genera in at least one site within the region (Figure 3a). The average composition of *Entosphenus* across all 23 tributary collection sites was 48.1% (range 0.0%–100.0%, Table 1).

The composition of lamprey genera was unstable within geographic regions: four of the six tributary regions (R03 WA coast, R04 estuarine Lower Columbia [LC], R05 mid LC, and R07 mid C) contained pairs of collections within the same region from the same collection year that had significantly different proportions of lamprey genera (i.e., nonoverlapping 99% CI, Figure S5). The largest differences between sites within the same region were observed on the R03 “WA coast” (> 80% proportional difference between Bingham and Newaukum, sites 3 and 4, respectively, Figure S5) and within the R07 “mid C” (> 50% proportional difference between Hamilton and Big White Salmon, sites 13 and 15, respectively, Figure S5).

There was also evidence of temporal instability in the composition of lamprey genera, which was indicated by two pairs of temporal samples (two unique sites 9 and 10) sampled in two different years (2019 and 2020) within the R05 “mid LC” geographic region that had significantly different proportions of *Entosphenus* (Figure S5). For all three collection sites in the R05 “mid LC” region (sites 8, 9, and 10) there were consistently lower proportions of *Entosphenus* in 2020 as compared to 2019, and the proportions across sites showed a consistent trend from low to high with increasing Rkm distance each year (Figure S5).

#### 3.5.2 Composition of *Lampetra* Ecotypes: WBL vs. WRL

Among the 13 tributary sites that contained the *Lampetra* genus, based on the candidate 5‐SNP ID, there were only four sites (sites 2, 4, 9, 12) in which both ecotype IDs were present, and the other nine sites (sites 3, 5, 6, 7, 8, 10, 13, 14, and 15) contained only the WBL ecotype (Table 1, Figure 3b). All regional groupings of tributary sites observed both candidate ecotype IDs in at least one site within each region, except for the most downstream region (R04 “estuarine LC”, Figure 3b) and the most upstream region (R07 “mid C”, Figure 3b). The average composition of WBL across all 23 tributary collections was 93.0% (range 33.9%–100.0%), and WRL was 5.6% (range 0.0%–57.1%) and intermediate was 1.3% (range 0.0%–8.9%). Potential for introgression between ecotypes was observed by plotting individual‐level composition of WRL alleles across all collections (Figure S6). Most of the individual fish (> 90%) occupied the extreme ends of the proportional composition of WRL alleles at the five candidate SNPs (e.g., > 70% or < 30% WRL alleles for WRL and WBL classifications, respectively, Figure S6); the remainder of individuals (< 10% of *Lampetra* specimen) with introgressed composition of WRL alleles (i.e., between 30% and 70%) were mostly found within Ksi Ts'oohl Ts'ap Creek (R02, 02ZOLZAP; Figure S6).

The composition of *Lampetra* ecotypes was extremely stable within geographic regions: none of the comparisons between collections from the same collection year had significantly different proportions of ecotypes within the six tributary regions (WBL proportions, Figure S7; WRL proportions, Figure S8). Further, there was minimal evidence of temporal instability in the compositions of *Lampetra* ecotypes, i.e., all temporal samples from each site had overlapping 99% CI except for site 09 Abernathy between collection year 2019 (WBL 100.0%) and 2020 (WBL 90.0%; CI:78.0%–98.0%; Figures S7 and S8).

Tests for correlation of the distance from the mouth of the Columbia River with frequencies of *Lampetra* candidate ecotype alleles showed a slight negative linear relationship of upstream distance with the WRL allele frequencies at three of the five candidate SNPs (SNPs on chromosomes 2 and 14; Figure S9) and showed positive linear relationships of upstream distance with the WRL allele frequencies at two of the five candidate SNPs (SNPs on chromosomes 5 and 12; Figure S9); The Mantel *r* values were significant for the two SNPs with positive linear relationships (Lri5P4640927 *r* = 0.893, *p* < 0.001; Lri12P6060911 *r* = 0.456, *p* < 0.01), but the Mantel *r* values were not significant for the SNPs with negative linear relationships.

Outside of the Georgia Basin and Ksi Ts'oohl Ts'ap Creek, only Newaukum (04NEWAUK19), Abernathy (09ABNTHY20), and Cowlitz (12COWLIT19) contained any WRL candidate genetic IDs and in each of these sites there was only a single individual WRL in the collections. In the case of the Cowlitz, there was one adult WRL that was collected in 2019, which was also identified morphologically from photos (Figure S4). In the case of Newaukum (R03 “WA coast” region) and Abernathy (R05 “mid LC” region), one 87 mm larval WRL and one 153 mm juvenile WRL were confirmed by genotypes in those respective sites but had no photo documentation. In the case of Newaukum, given its early life stage it would not have been possible to use morphology to determine WBL versus WRL.

#### 3.5.3 Population Genetic Structure Between *Lampetra* Ecotypes

There were a total of 47 GT seq genotypes and 835 WGS estimated individual genotypes that passed 70% thresholds of missing data and produced a combined SNP dataset of 882 loci. The DAPC showed support for a single cluster (*k* = 1) based on the Bayesian Information Criterion (BIC, Figure S10). Even when using only the trimmed dataset of 303 WGS estimated individual genotypes, the results were the same (i.e., BIC support for *k* = 1, Figure S10).

The BayeScan analysis showed that the highest probability scores (> 0.70) for potential *F*_ST_ outliers were only among a subset (*N* = 10) of the 47 GT seq candidate SNPs, and this subset contained all five of the SNPs we had chosen to develop into a 5‐SNP genetic ID (Figure S11). Further, the only SNPs that had a probability above 90% were SNPs (Lri2P16367064 and Lri14P4079387). The top outlier SNPs (Probability > 0.7) had an *F*_ST_ average of 0.07 (range 0.05–0.09); outside of the top outlier SNPs (Probability < 0.7), the remaining 872 SNPs were extremely low *F*_ST_ with an average of 0.01 (range 0.01–0.05).

## 4 Discussion

Evidence from our study corroborates the recent hypothesis by Carim et al. (2023) that the paired species, WBL and WRL described within the genus *Lampetra*, are ecotypes within the same species as opposed to distinct genetic lineages. Whole genome resequencing (WGS) in our study demonstrated that morphologically defined specimens from a single tributary, Ksi Ts'oohl Ts'ap Creek in Northern B.C. Canada, were distinguished by narrow regions of genomic divergence (i.e., “genomic islands of divergence”) like those associated with maturation and body size ecotypes of Pacific Lamprey (Hess et al. 2020). This pattern is in contrast with the genomic pattern expected if the ecotypes had represented distinct genetic lineages or separate species. Distinct genetic lineages with minimal gene flow exchange between them would lead to high genomic divergence across all chromosomes and not just within narrow regions on a subset of chromosomes (Yeaman and Whitlock 2011). Our tests using individual genotypes from the WGS dataset also supported that WBL and WRL ecotypes in the Ksi Ts'oohl Ts'ap represent a single population and only the small subset of candidate SNPs appear to be highly diverged relative to other SNPs randomly distributed across the genome (882 SNPs that could be genotyped with this low coverage dataset). The polyphyletic lineages of WBL and WRL would not pass definitions of species based on a purely phylogenetic species concept (e.g., Cracraft 1983). As such, Carim et al. (2023) proposed to synonymize WRL and WBL as alternate life histories of *L. ayresii* , the first of the two species which was described in 1870 (Vladykov and Follet 1958). Our results lend further support to this taxonomic revision.

One prominent genomic island of divergence was located on chromosome 2 of the *Lampetra richardsoni* reference genome, and within that region, we developed two of the candidate SNPs that comprised our presumptive genetic ID for WBL and WRL (SNP, Lri2P16367064 and Lri2P16333005). We combined these two SNPs on chromosome 2 with three others on chromosomes 5, 12, and 14 to develop a 5‐SNP candidate assay that we showed to have high potential for accurate ID of the WBL and WRL ecotypes at all life stages, as evidenced by high concordance between morphological and genetic ID of these ecotypes (> 98%) in collections of mixed native lampreys. In addition, we found this 5‐SNP candidate assay identified 100% of the WRL ecotypes correctly for the “positive control” ocean‐phase WRL collection from the Georgia Basin. It is important to note that although the concordance of genetic and morphological IDs was less than 100% and the genetic ID leads to a small (< 3% of all genotyped specimen) number of ambiguous (heterozygous genotypes or “intermediate”) classifications, it can determine > 97% of all genotyped specimens into definitive categories with high accuracy. This is a substantial step forward beyond reliance on morphological ID, which resolves 0% of *Lampetra* larvae specimens, the most readily observed and collected life stage. Therefore, genetic IDs developed here will significantly enhance our understanding of the distribution of *Lampetra* ecotypes across the species range.

Tributaries in which the WBL and WRL ecotypes were confirmed to be sympatric based on morphological ID, i.e., Ksi Ts'oohl Ts'ap Creek (British Columbia, Canada) and a single individual from the Cowlitz River (Southwestern Washington, U.S.), have also been confirmed as having a “WRL” genetic ID according to our candidate 5‐SNP assay. In the case of Ksi Ts'oohl Ts'ap Creek, the degree of sympatry represents the most direct evidence to date that these ecotypes can co‐exist in the same stream and possibly even originate from the same population. However, more work will have to be conducted to explore alternative hypotheses to ecotypes such as incomplete lineage sorting or incipient species. Evaluation of alternative hypotheses would require testing for the potential existence (if any) and characteristics of isolating mechanisms that may operate between ecotypes. Ksi Ts'oohl Ts'ap Creek may be a suitable study site for this kind of future work because it exhibited the closest balance in frequencies of the WBL and WRL ecotypes (i.e., having near equal frequencies of WRL and WBL) out of all collection sites surveyed in this study.

The 5‐SNP candidate assay developed in this study offers an opportunity for holistic examination of the ecotypes and their biology and distribution, and our initial results have led to additional questions that need to be resolved, including: (1) how universal is the 5‐SNP candidate assay for genetic ID of anadromous ecotypes of lampreys within Western North American *Lampetra* and more distantly related lampreys?, (2) where do WRL that are found in the Lower Columbia originate?, and (3) what factors influence the compositions of lamprey genera and ecotypes?

### 4.1 How Universal Is the Candidate SNP for Genetic ID of Anadromous Ecotypes of Lampreys?

There are a number of requisite traits for parasitic/anadromous ecotypes that would be unnecessary for a nonparasitic/resident life, including traits related to saltwater tolerance (i.e., marine osmoregulatory ability, Ferreira‐Martins et al. 2016) and parasitism (i.e., sharp teeth and gut development; Docker et al. 2012). These requisite traits would likely be similar across paired lampreys, even those occupying different phylogenetic branches (Brownstein and Near 2023). We anticipated that genomic regions which distinguish the Western North American *Lampetra* ecotypes in our study might be shared with the European *Lampetra* paired species (Souissi et al. 2022). However, the four loci identified by Souissi et al. (2022) were localized to *Lampetra richardsoni* genome scaffold 54 (synteny with sea lamprey chromosome 64), which was not differentiated between WBL and WRL ecotypes in our study. This observation helps link all four of the independent Souissi et al. (2022) markers to a single chromosome and provides a defined genomic region to investigate for the ecotypes of European *Lampetra*. In contrast, we found at least 10 different chromosomal regions that differed between WBL and WRL ecotypes. This suggests that the underlying genetic basis for paired species, specifically anadromous/parasitic ecotypes, may not be conserved across lampreys. Instead, there are likely different mechanisms that can lead to the evolution of lamprey ecotypes in different lineages but can also involve genes of large effect. The fact that there are several extended regions of high *F*_ST_ (genomic islands) distributed across separate chromosomes also suggests that a single marker representing just one genomic island might not be sufficient to capture the full variation that is required to identify WRL across this taxon's geographic range.

The candidate marker (Lri2P16367064) from the prominent genomic island on chromosome 2 that we assayed across collections was in an interval that is 2479 bp downstream of a synaptic vesicle glycoprotein 2C gene (SV2C; XM_032969849.1) and was part of a region of high *F*_ST_ that encompassed the entire gene. These vesicles store neurotransmitters whose release is regulated by voltage‐dependent calcium channels (Rossi et al. 2022). The genetic association of anadromy with variants near the SV2C gene and calcium channels may be functionally related to the expression of *Lampetra* ecotypes. Anadromous WRL, like other anadromous fishes including salmonids (Jamieson et al. 2021), must maintain osmotic homeostasis in both fresh water and saltwater environments, where drastically different calcium and sodium ion concentrations occur. Recent studies have underscored the importance of voltage‐dependent calcium channels in the catadromous marbled eel, where expression of genes coding for calcium channels was upregulated in high calcium treatments ( *Anguilla marmorata* , Cao et al. 2020). The genetic locus we identified to distinguish between WBL and WRL warrants further investigation to elucidate the functional differences these gene variants play in the expression of the *Lampetra* ecotypes.

Because two candidate SNPs in North American *Lampetra* ecotypes appear to be localized nearby a key gene related to osmotic homeostasis in varying calcium environments, the chances would be high that this candidate genetic ID is useful across the range of these ecotypes as long as the phenotypic change is largely driven by parallel genetic changes. The NE Pacific range of WRL extends from Alaska to California (Carim et al. 2023). It will be important to determine the utility of the candidate marker across this entire range, or if it is only suited for the northern portion where our study occurred. In fact, Souissi et al. (2022) found that the candidate SNP they identified to distinguish ecotypes in European river and brook lampreys was not reliable from samples from the Rhone drainage and Loch Lomond. There can also be issues with using small sample sizes like we used in our study because it can lead to inaccurate allele frequency estimates (e.g., Lou et al. 2021); in our case, validation of candidate markers showed marker selection was successful, nonetheless. However, future studies should strive for high sample sizes to have the best chances for success. We also note that there could be ascertainment bias from developing candidate SNPs from specimens in a single, northern population. Our results provide reasons for optimism regarding utility outside of the northern range given that genetic and morphological ID concordance was found over a wide geographic range encompassing Northern B.C., the Georgia Basin, Western Washington, and lower Columbia tributaries. Nonetheless, future sampling from localities in the southern end of the species range will be crucial for verifying the extent of utility for the candidate genetic ID.

There are likely multiple genes involved in this complex life history, and by examining the regions of three other chromosomes (i.e., *Lampetra richardsoni* chromosomes 5, 12, and 14) in which the other candidate genetic ID SNPs localized, we may be able to gain additional insights into relevant functions involved in the trait beyond osmoregulatory ability. The candidate SNPs from these three chromosomes were nearby genes NIN (ninein, chromosome 5), ACVR2B (Activin receptor type‐2B; chromosome 12), and ESR1 (Estrogen Receptor; chromosome 14). Previously described functions of these genes do not all have obvious relevance to the ecotype life histories or any functions elucidated for fishes in general. The details that are available suggest these genes may be involved with transcriptional regulation and functioning in centrosomes in humans (NIN, Stillwell et al. 2004), cell surface receptor that regulates muscle growth and other processes in fishes (ACVR2B, Phelps et al. 2013), and estrogen receptors which have been found to function in both males and females and play roles in biological processes including migration and reproduction in anadromous fishes (ESR1, reviewed in Nikoleris and Hansson 2015). Additional work is needed to elucidate the relevance of these genomic regions with *Lampetra* life history.

### 4.2 Where Do WRL That Are Found in the Lower Columbia Originate?

Previous work has demonstrated the presence of WRL in the Columbia River estuary (Weitkamp et al. 2015). However, it is unclear where potential spawning sources of WRL might be located in the Columbia River basin because WRL have not been detected at screw traps in the past (either due to their rarity or lack of training of field crews or both). The only site in the Lower Columbia in our study that confirmed a WRL adult (Cowlitz River) was part of a region that had low relative abundance of *Lampetra* compared to *Entosphenus*. In general, WRL were found in only two other instances among collections from the WA coast and Lower Columbia combined. This low incidence of genetic IDs of WRL and low frequencies of WRL alleles suggest that the WRL captured in the Cowlitz River may have been a stray or migrant from a different population of *Lampetra* in which WRL alleles were higher in frequency. Moderately higher frequencies of WRL alleles occur in tributaries further downstream in the Columbia River nearer to the Pacific Ocean and may be one potential source of WRL genotypes (albeit at low rates). Another possible source would be from long distance migration from a northern population (i.e., Nass River, northern British Columbia or tributaries of the Salish sea) where we observed much higher frequencies of WRL genes and WRL have been reported to be more abundant (Hayes et al. 2013). It will be important to resolve this mystery in the future using more genetic markers to better understand where WRL originate, whether there are important sources for spawning WRL in the Lower Columbia, and whether the Columbia River estuary serves mainly as a feeding ground or population sink for WRL strays from other locations.

This question requires some understanding of the relationship between ecotypes within tributaries where they are both abundant. Evidence from the WGS data from the population of our study with a near perfect balance in frequency of the two ecotypes (Ksi Ts'oohl Ts'ap Creek in Northern B.C) suggests that these ecotypes are sympatric and act as a single panmictic population capable of producing both ecotypes (WBL and WRL). However, there is evidence from other studies that there may be pre‐mating reproductive isolating mechanisms, especially size assortative mating, at work to drive the separation of these ecotypes over time (Beamish and Neville 1992; Docker 2009). Future work should examine the population in Ksi Ts'oohl Ts'ap to understand whether there may be pre‐ or post‐mating isolating mechanisms that may be too weak for detection in this study but could be detected with greater numbers of markers and more voucher specimens.

### 4.3 What Factors Influence the Compositions of Lamprey Genera and Ecotypes?

We had two guiding expectations regarding compositions of lamprey genera and ecotypes. The first was that among the anadromous lampreys, the Pacific Lamprey distribution is the most ubiquitous and WRL's abundance is more northerly distributed (USFWS 2018); both of these described distributions validated similar distribution patterns observed from our results. The second expectation was that the comparison of distributions of the candidate alleles of *Lampetra* ecotypes may reveal a more coastal abundance of the alleles associated with anadromous WRL relative to the resident WBL. This expectation was not supported by our results because there were inconsistent patterns across the five candidate SNPs. Upstream distance did appear to show a negative linear relationship with the WRL allele frequencies for three of the candidate SNPs (chromosomes 2 and 14), but the patterns were not significant correlations. However, we found the opposite trend and significant positive linear relationships with two candidate SNPs (chromosomes 5 and 12). The inconsistent patterns may be partly due to the diversity in functions represented by these genes (described above) because they may not all be directly related to anadromy. It may also be helpful to expand the geographic scope with more data from collections obtained further upstream to test for a more robust pattern as we expand further into the distributions of the ecotypes. WBL are relatively abundant upstream of Bonneville Dam (Spice et al. 2019); WRL have also been observed in areas further upstream of the Columbia River, albeit more rarely (John Day Dam at Rkm 348, Jolley et al. 2016).

Proportions of *Lampetra* ecotypes were more likely to be similar when comparing across years from the same site (i.e., temporal stability), or across sites from the same region (i.e., spatial stability); this was a contrast to the relative instability of proportions of lamprey genera (i.e., *Entosphenus* vs. *Lampetra*) both temporally and spatially. This observation may be simply because the abundances of *Entosphenus* and *Lampetra* are independent of each other; however, the abundances of the ecotypes within *Lampetra* act dependently since they are segments within one population. This does underscore the importance of having multiple spatial and temporal samples to establish reliable presence/absence data for lamprey genera across neighboring tributaries and streams within geographic regions.

It is also interesting to note that there are streams in which anadromous lampreys (Pacific Lamprey and WRL) are present but absent in neighboring streams that are dominated by the resident WBL. For example, the Chehalis River basin in Region 03 on “WA coast” had one stream with Pacific Lamprey and WRL and another that is dominated by WBL. Another example is Region 06, which had high proportions of anadromous taxa, and neighboring regions immediately upstream and downstream (Regions 05 and 07) had higher portions of resident WBL. This study identified tributaries that could be surveyed more intensively to understand what stream characteristics may promote anadromy versus residence or possibly to identify reaches where passage barriers may be present that prevent anadromous taxa from occupying them.

### 4.4 Management Implications

“Messy” species boundaries are not unique to lampreys, and some precedent exists for how to navigate their conservation management. There are examples from salmonids where segments of populations thought of as ecotypes rather than separate lineages or species have been granted resources dedicated to understanding relative abundance and distribution across their range (e.g., early and late migration ecotypes in Chinook Salmon from the Central Valley, CA; Baerwald et al. 2023). Decisions on whether to split a particular species or taxa into smaller subcomponents (i.e., conservation units) require an understanding of the role each potential unit may play in the ecosystem; the ecosystem role of each conservation unit and its habitat requirements should be unique (i.e., difference in niche). In the case of lampreys, specifically WBL and WRL, they represent a major contrast in their role and requirements in habitat: WBL is a freshwater resident and nonparasitic, and WRL is anadromous and parasitic. Docker and Hume (2019) discussed the value of conserving lamprey diversity below the species level, and Carim et al. (2023) suggested maintaining the common names of WRL and WBL (despite them being a single Linnaean species) to recognize the ecotypic diversity. In discussing the conservation implications of ecotypic diversity in silver and northern brook lampreys, Docker et al. (2012) raised valuable questions to consider, including whether one ecotype can be rehabilitated from the other and whether the migratory ecotype is needed to mediate gene flow between isolated brook lamprey populations. These questions are applicable for *Lampetra* ecotypes as well. This recognition of ecotypic diversity in Western North American *Lampetra* makes it critical to have methods for distinguishing ecotypes when conducting surveys. In doing so, this would comprehensively account for diversity in lampreys important for all the roles the three focal taxa play within the ecosystem. However, regardless of their identification, lampreys globally are not doing well, in large part due to deleterious changes in habitat. Even if we cannot identify them correctly all the time, knowledge of the breadth of habitat types and qualities associated with all three of these focal lampreys will help guide general ways to protect habitat to foster maximum diversity.

## 5 Conclusions

We have new ability to equip field crews with a genetic ID and allow greater breadth and accuracy of information from three focal lampreys that can be applied efficiently in tributaries of the NE Pacific. This ability will help to comprehensively characterize the diversity of lampreys across the NE Pacific by accurate identification at the genus level as well as at an intraspecific level to survey ecotypic diversity. Genetic ID of anadromous ecotypes of lampreys is a novel approach that may yield insight into the niches that these ecotypes inhabit as well as the passage and habitat quality factors that may limit their distributions.

## Ethics Statement

All lampreys were handled to ensure humane treatment that minimized discomfort, stress, and pain by following the guidelines covered by the research permits held by Nisga'a Fisheries and Wildlife, US Geological Survey, and Washington Department of Fish and Wildlife as well as the protocols specific to lampreys distributed by Yakama Nation under their Bonneville Power Administration Project (Project No. 2008‐470‐00).

## Conflicts of Interest

The authors declare no conflicts of interest.

## Supporting information


Appendix S1

## Data Availability

*Lampetra richardsoni* whole genome sequence: GenBank accession number: JARYGF000000000, JASBGX000000000. WBL and WRL whole genome resequencing data: GenBank Accession Number PRJNA953978. Sampling locations, SNP genotypes, and SNP primer sequences: Dryad DOI https://doi.org/10.5061/dryad.b2rbnzss7.

## References

[eva70108-bib-0045] Arakawa, H. , R. T. Lampman , and J. E. Hess . 2021. “Whose Kids Did You Eat? Genetic Identification of Species and Parents of Larval Lampreys in Fish Predator Guts.” Transactions of the American Fisheries Society 150, no. 5: 551–559.

[eva70108-bib-0041] Auringer, G. , M. A. Campbell , P. A. Goertler , and A. J. Finger . 2023. “Lampreys in California (Lampetra spp. and Entosphenus spp.): Mitochondrial Phylogenetic Analysis Reveals Previously Unrecognized Lamprey Diversity.” North American Journal of Fisheries Management 43, no. 6: 1511–1530.

[eva70108-bib-0001] Baerwald, M. R. , E. C. Funk , A. M. Goodbla , et al. 2023. “Rapid CRISPR‐Cas13a Genetic Identification Enables New Opportunities for Listed Chinook Salmon Management.” Molecular Ecology Resources. 10.1111/1755-0998.13777. PMC12142720 36847138

[eva70108-bib-0055] Beamish, R. J. , and C. E. M. Neville . 1992. “The Importance of Size as an Isolating Mechanism in Lampreys.” Copeia: 191–196.

[eva70108-bib-0002] Beamish, R. , R. Withler , J. Wade , and T. Beacham . 2016. “Chapter Eight: A Nonparasitic Lamprey Produces a Parasitic Life History Type: The Morrison Creek Lamprey Enigma.” In Jawless Fishes of the World: Volume 1, 191. Cambridge Scholars Publishing.

[eva70108-bib-0003] Boguski, D. A. , S. B. Reid , D. H. Goodman , and M. F. Docker . 2012. “Genetic Diversity, Endemism and Phylogeny of Lampreys Within the Genus Lampetra Sensu Stricto (Petromyzontiformes: Petromyzontidae) in Western North America.” Journal of Fish Biology 81: 1891–1914. 23130690 10.1111/j.1095-8649.2012.03417.x

[eva70108-bib-0004] Brownstein, C. D. , and T. J. Near . 2023. “Phylogenetics and the Cenozoic Radiation of Lampreys.” Current Biology 33, no. 2: 397–404. 36586410 10.1016/j.cub.2022.12.018

[eva70108-bib-0043] Campbell, N. R. , S. A. Harmon , and S. R. Narum . 2015. “Genotyping‐in‐Thousands by Sequencing (GT‐Seq): A Cost Effective SNP Genotyping Method Based on Custom Amplicon Sequencing.” Molecular Ecology Resources 15, no. 4: 855–867. 25476721 10.1111/1755-0998.12357

[eva70108-bib-0005] Cao, Q. , P. Chu , J. Gu , et al. 2020. “The Influence of Ca^2+^ Concentration on Voltage‐Dependent L‐Type Calcium Channels' Expression in the Marbled Eel (*Anguilla marmorata*).” Gene 722: 144101. 10.1016/j.gene.2019.144101. 31479714

[eva70108-bib-0006] Caputo, V. , M. Giovannotti , P. N. Cerioni , A. Splendiani , J. Tagliavini , and E. Olmo . 2011. “Chromosomal Study of a Lamprey ( *Lampetra zanandreai* Vladykov, 1955) (Petromyzonida: Petromyzontiformes): Conventional and FISH Analysis.” Chromosome Research 19: 481–491. 21437736 10.1007/s10577-011-9197-4

[eva70108-bib-0007] Carim, K. J. , G. Auringer , M. F. Docker , et al. 2024. “Species Diversity in the New Lamprey Genus *Occidentis*, Formerly Classified as Western North American ‘Lampetra’.” PLoS One 19, no. 12: e0313911. 39700072 10.1371/journal.pone.0313911 PMC11658800

[eva70108-bib-0008] Carim, K. J. , D. C. Larson , J. M. Helstab , M. K. Young , and M. F. Docker . 2023. “A Revised Taxonomy and Estimate of Species Diversity for Western North American *Lampetra* .” Environmental Biology of Fishes 106: 817–836.

[eva70108-bib-0009] Clemens, B. J. , and C. B. Schreck . 2021. “An Assessment of Terminology for Intraspecific Diversity in Fishes, With a Focus on ‘Ecotypes’ and ‘Life Histories’.” Ecology and Evolution 11, no. 16: 10772–10793. 34429881 10.1002/ece3.7884 PMC8366897

[eva70108-bib-0035] Clemens, B. J. , and J. Wade . 2023. “Conservation Biology of the Lampetra Species Complex of Western North America, with a Focus on Western Brook Lamprey (*L. richardsoni*).” Canadian Manuscript Report of Fisheries and Aquatic Sciences 3258: vi + : 26.

[eva70108-bib-0051] Cracraft, J. 1983. “Species Concepts and Speciation Analysis.” In Current Ornithology, edited by R. Johnston , 159–187. Plenum Press.

[eva70108-bib-0037] Dawson, H. A. , B. R. Quintella , P. R. Almeida , A. J. Treble , and J. C. Jolley . 2015. “The Ecology of Larval and Metamorphosing Lampreys.” Lampreys: Biology, Conservation and Control 1: 75–137.

[eva70108-bib-0011] Docker, M. F. 2009. “A Review of the Evolution of Nonparasitism in Lampreys and an Update of the Paired Species Concept.” In Biology, Management, and Conservation of Lampreys in North America, edited by L. R. Brown , S. D. Chase , P. B. Moyle , R. J. Beamish , and M. G. Mesa , 71–114. American Fisheries Society Symposium 72.

[eva70108-bib-0056] Docker, M. F. , and J. B. Hume . 2019. “There and Back Again: Lampreys in the 21st Century and Beyond.” Lampreys: Biology, Conservation and Control 2: 527–570.

[eva70108-bib-0012] Docker, M. F. , N. E. Mandrak , and D. D. Heath . 2012. “Contemporary Gene Flow Between ‘Paired’ Silver ( *Ichthyomyzon unicuspis* ) and Northern Brook ( *I. fossor* ) Lampreys: Implications for Conservation.” Conservation Genetics 13: 823–835.

[eva70108-bib-0013] Docker, M. F. , and I. C. Potter . 2019. “Life History Evolution in Lampreys: Alternative Migratory and Feeding Types.” In Lampreys: Biology, Conservation and Control: Volume 2, edited by M. F. Docker , I. C. Potter , M. F. Docker , and I. C. Potter , 287–409. Springer Netherlands.

[eva70108-bib-0014] Docker, M. F. , G. S. Silver , J. C. Jolley , and E. K. Spice . 2016. “Simple Genetic Assay Distinguishes Lamprey Genera *Entosphenus* and *Lampetra*: Comparison With Existing Genetic and Morphological Identification Methods.” North American Journal of Fisheries Management 36, no. 4: 780–787.

[eva70108-bib-0015] Ferreira‐Martins, D. , J. Coimbra , C. Antunes , and J. M. Wilson . 2016. “Effects of Salinity on Upstream‐Migrating, Spawning Sea Lamprey, *Petromyzon marinus* .” Conservation Physiology 4, no. 1: cov064. 27293744 10.1093/conphys/cov064 PMC4765514

[eva70108-bib-0050] Foll, M. , and O. Gaggiotti . 2008. “A Genome‐Scan Method to Identify Selected Loci Appropriate for Both Dominant and Codominant Markers: A Bayesian Perspective.” Genetics 180, no. 2: 977–993. 10.1534/genetics.108.092221. 18780740 PMC2567396

[eva70108-bib-0039] Goodman, D. H. , A. P. Kinziger , S. B. Reid , and M. F. Docker . 2009. “Morphological Diagnosis of *Entosphenus* and *Lampetra* Ammocoetes (Petromyzontidae) in Washington, Oregon, and California.” In Biology, Management, and Conservation of Lampreys in North America, edited by L. R. Brown , S. D. Chase , M. G. Mesa , R. J. Beamish , and P. B. Moyle , 223–232. American Fisheries Society, Symposium 72.

[eva70108-bib-0054] Hayes, M. C. , R. Hays , S. P. Rubin , et al. 2013. “Distribution of Pacific Lamprey *Entosphenus tridentatus* in Watersheds of Puget Sound Based on Smolt Monitoring Data.” Northwest Science 87, no. 2: 95–105.

[eva70108-bib-0042] Hess, J. E. , N. R. Campbell , D. A. Close , M. F. Docker , and S. R. Narum . 2013. “Population Genomics of Pacific Lamprey: Adaptive Variation in a Highly Dispersive Species.” Molecular Ecology 22, no. 11: 2898–2916. 23205767 10.1111/mec.12150

[eva70108-bib-0040] Hess, J. E. , N. R. Campbell , M. F. Docker , et al. 2015. “Use of Genotyping by Sequencing Data to Develop a High‐Throughput and Multifunctional SNP Panel for Conservation Applications in Pacific Lamprey.” Molecular Ecology Resources 15, no. 1: 187–202. 24842551 10.1111/1755-0998.12283

[eva70108-bib-0036] Hess, J. E. , T. A. Delomas , A. D. Jackson , et al. 2022. “Pacific Lamprey Translocations to the Snake River Boost Abundance of All Life Stages.” Transactions of the American Fisheries Society 151, no. 3: 263–296.

[eva70108-bib-0044] Hess, J. E. , J. J. Smith , N. Timoshevskaya , et al. 2020. “Genomic Islands of Divergence Infer a Phenotypic Landscape in Pacific Lamprey.” Molecular Ecology 29, no. 20: 3841–3856. 32814354 10.1111/mec.15605

[eva70108-bib-0046] Horn, R. L. , C. Kamphaus , K. Murdoch , and S. R. Narum . 2020. “Detecting Genomic Variation Underlying Phenotypic Characteristics of Reintroduced Coho Salmon (*Oncorhynchus kisutch*).” Conservation Genetics 21, no. 6: 1011–1021.

[eva70108-bib-0017] Hughes, L. C. , D. D. Bloom , K. R. Piller , N. Lang , and R. L. Mayden . 2025. “Phylogenomic Resolution of Lampreys Reveals the Recent Evolution of an Ancient Vertebrate Lineage.” Proceedings of the Royal Society B: Biological Sciences 292, no. 2038: 20242101. 10.1098/rspb.2024.2101 PMC11706654 39772957

[eva70108-bib-0018] Jamieson, L. , A. Waters , K. E. Ho , et al. 2021. “Short‐Term Homeostatic Regulation of Blood/Interstitial Fluid Ca^2+^ Concentration by the Scales of Anadromous Sea Trout *Salmo trutta L*. During Smoltification and Migration.” Journal of Fish Biology 98, no. 1: 17–32. 32964432 10.1111/jfb.14553

[eva70108-bib-0019] Jolley, J. C. , G. Kovalchuk , and M. F. Docker . 2016. “River Lamprey (*Lampetra ayresii*) Outmigrant Upstream of the John Day Dam in the Mid Columbia River.” Northwest Science 97: 48–52.

[eva70108-bib-0049] Jombart, T. , S. Devillard , and F. Balloux . 2010. “Discriminant Analysis of Principal Components: A New Method for the Analysis of Genetically Structured Populations.” BMC Genetics 11: 1–15. 20950446 10.1186/1471-2156-11-94 PMC2973851

[eva70108-bib-0020] Lampman, R. 2018. “Columbia Basin Lamprey Identification Guide.” Appendix 4.3/L1 in Yakama Nation Pacific Lamprey Project 2017 Annual Progress Report (Cooperative Agreement No. R15AC00044/Project No. 2008‐470‐00), 3 pp. Prepared for the U.S. Dept. of Interior, Bureau of Reclamation, Boise, ID, and U.S. Dept. of Energy, Bonneville Power Administration, Portland, OR.

[eva70108-bib-0021] Lou, R. N. , A. Jacobs , A. P. Wilder , and N. O. Therkildsen . 2021. “A Beginner's Guide to Low‐Coverage Whole Genome Sequencing for Population Genomics.” Molecular Ecology 30, no. 23: 5966–5993. 34250668 10.1111/mec.16077

[eva70108-bib-0031] Luzier, C. W. , H. A. Schaller , J. K. Brostrom , et al. 2011. Pacific Lamprey Assessment and Template for Conservation Measures. U.S. Fish and Wildlife Service. www.fws.gov/columbiariver/publications.html.

[eva70108-bib-0047] Micheletti, S. J. , and S. R. Narum . 2018. “Utility of Pooled Sequencing for Association Mapping in Nonmodel Organisms.” Molecular Ecology Resources 18, no. 4: 825–837. 29633534 10.1111/1755-0998.12784

[eva70108-bib-0022] Nikoleris, L. , and M. C. Hansson . 2015. “Unraveling the Estrogen Receptor (Er) Genes in Atlantic Salmon ( *Salmo salar* ) Reveals Expression Differences Between the Two Adult Life Stages but Little Impact From Polychlorinated Biphenyl (PCB) Load.” Molecular and Cellular Endocrinology 400: 10–20. 25451980 10.1016/j.mce.2014.11.009

[eva70108-bib-0053] Phelps, M. P. , I. M. Jaffe , and T. M. Bradley . 2013. “Muscle Growth in Teleost Fish Is Regulated by Factors Utilizing the Activin II B Receptor.” Journal of Experimental Biology 216, no. 19: 3742–3750. 23788712 10.1242/jeb.086660

[eva70108-bib-0034] Potter, I. C. , H. S. Gill , C. B. Renaud , and D. Haoucher . 2015. “The Taxonomy, Phylogeny, and Distribution of Lampreys.” Lampreys: Biology, Conservation and Control 1: 35–73.

[eva70108-bib-0030] Renaud, C. B. 1997. “Conservation Status of Northern Hemisphere Lampreys (Petromyzontidae).” Journal of Applied Ichthyology 13, no. 3: 143–148.

[eva70108-bib-0038] Richards, J. E. , R. J. Beamish , and F. W. H. Beamish . 1982. “Descriptions and Keys for Ammocoetes of Lampreys from British Columbia, Canada.” Canadian Journal of Fisheries and Aquatic Sciences 39, no. 11: 1484–1495.

[eva70108-bib-0023] Rossi, R. , S. Arjmand , S. L. Bærentzen , A. Gjedde , and A. M. Landau . 2022. “Synaptic Vesicle Glycoprotein 2A: Features and Functions.” Frontiers in Neuroscience 16: 864514. 35573314 10.3389/fnins.2022.864514 PMC9096842

[eva70108-bib-0033] Scott, W. B. , and E. J. Crossman . 1973. Freshwater Fishes of Canada. Bulletin. Vol. 184, 966. Fisheries Research Board of Canada. (also 1979 reprint).

[eva70108-bib-0024] Souissi, A. , A. L. Besnard , and G. Evanno . 2022. “A SNP Marker to Discriminate the European Brook Lamprey ( *Lampetra planeri* ), River Lamprey ( *L. fluviatilis* ) and Their Hybrids.” Molecular Biology Reports 49: 10115–10119. 10.1007/s11033-022-07800-8. 36057877

[eva70108-bib-0025] Spice, E. K. , T. A. Whitesel , G. S. Silver , and M. F. Docker . 2019. “Contemporary and Historical River Connectivity Influence Population Structure in Western Brook Lamprey in the Columbia River Basin.” Conservation Genetics 20: 299–314.

[eva70108-bib-0026] Stillwell, E. E. , J. Zhou , and H. C. Joshi . 2004. “Human Ninein Is a Centrosomal Autoantigen Recognized by CREST Patient Sera and Plays a Regulatory Role in Microtubule Nucleation.” Cell Cycle 3, no. 7: 923–930. 15190203

[eva70108-bib-0048] Timoshevskaya, N. , K. İ. Eşkut , V. A. Timoshevskiy , et al. 2023. “An Improved Germline Genome Assembly for the Sea Lamprey *Petromyzon marinus* Illuminates the Evolution of Germline‐Specific Chromosomes.” Cell Reports 42, no. 3. 10.1016/j.celrep.2023.112263 PMC10166183 36930644

[eva70108-bib-0027] US Fish and Wildlife Service (USFWS) . 2018. “Pacific Lamprey *Entosphenus tridentatus* Assessment.” February 28, 2019 Final Draft. https://www.sciencebase.gov.

[eva70108-bib-0052] Vladykov, V. D. , and W. I. Follett . 1958. “Redescription of *Lampetra ayresii* (Günther) of Western North America, a Species of Lamprey (Petromyzontidae) Distinct from *Lampetra fluviatilis* (Linnaeus) of Europe.” Journal of the Fisheries Board of Canada 15, no. 1: 47–77.

[eva70108-bib-0028] Weitkamp, L. A. , S. A. Hinton , and P. J. Bentley . 2015. “Seasonal Abundance, Size, and Host Selection of Western River ( *Lampetra ayresii* ) and Pacific ( *Entosphenus tridentatus* ) Lampreys in the Columbia River Estuary.” Fishery Bulletin 113, no. 2: 213–226.

[eva70108-bib-0029] Yeaman, S. , and M. C. Whitlock . 2011. “The Genetic Architecture of Adaptation Under Migration–Selection Balance.” Evolution 65, no. 7: 1897–1911. 21729046 10.1111/j.1558-5646.2011.01269.x

