# Supplementary material for: Genetic Identification of Lamprey Genera and Anadromous Ecotypes in Watersheds of the Northeastern Pacific Ocean
Source: Evol Appl. 2025 May 10;18(5):e70108. doi: 10.1111/eva.70108 (PMC12064929; doi:10.1111/eva.70108)
Supplement: Supplementary file 1 — Appendix S1 [file EVA-18-e70108-s001.pdf]

Supplemental Material

Assembly method

A new *Lampetra richardsoni* genome assembly was constructed using whole genome sequencing data from a female sample collected at Wapato Diversion, Yakima River (SAMN34128690). High molecular weight DNA was extracted from the blood by Amplicon Express (Pullman, WA, USA), and 10X sequencing was performed on an Illumina Nova-seq instrument (at University of Illinois Urbana-Champaign) yielding 737.64 million linked reads that provided 45.87X effective read coverage and an estimated mean molecule size of 136 Kb. The Supernova assembler v2.1 (Weisenfeld at al.2017) was employed with the option --maxreads='all'. The initial assembly output, generated with supernova mkoutput --style=pseudohap, was refined by removing identical contigs using the Perl program "fasta_uniqueseq.pl" (downloaded in 2019 and referred in the source code to www.ncbi.nlm.nih.gov/CBBresearch/Spouge/html.ncbi/fasta/list.html). These steps resulted in the assembly of approximately 1075 Megabases of sequence in 212792 scaffolds of at least 100 bases long with a scaffold's N50 3.8Mb and the longest scaffold reaching over 20Mb. This set of scaffolds was used for downstream SNP analyses.

We also applied the purge haplotigs algorithm to remove alternative haplotigs (Roach at al. 2018). First, linked reads were realigned to the assembly with bwa mem -a (v.0.7.17) and paired reads and alignments were filtered with samtools view, flag -F2308 (v.1.8) (Li 2009a,b). Genome coverage was computed using bedtools v2.27.1 (Quinlan and Hall 2010), and then tools contigcov and purge from purge_haplotigs suite were applied with parameters -l 5 -m 35 -h 200 -j 101 to identify and purge from the assembly scaffolds that represented alternative haplotype.

Following NCBI submission verification process, scaffolds were masked for the remaining candidate adapter sequence, filtered to exclude scaffolds that were <200 bp from primary (n = 55010) and alternative (n = 27567) assemblies, and to remove 296 primary and 549 alternative scaffolds that were reverse complements identical to other contigs. Scaffolds from the primary assembly are available under NCBI genome accession number JARYGF000000000 and alternative halpotypes are available under NCBI genome accession number JASBGX000000000.

Analysis of pool-seq data

Genomic DNA was extracted with a Chelex extraction technique (Sweet et al. 1996). Low-coverage whole genome resequencing utilized DNA that was normalized across a pool of samples, called a library, in which each individual sample received a unique barcode. Sequencing was performed on an Illumina HiSeq 500 with PE 300 sequencing reagents. The POOLPARTY pipeline (Micheletti and Narum 2018) was used to align reads back to the primary *L. richardsoni* assembly. Alignment filters in the PPalign module of POOLPARTY were set to a base quality minimum of 20, mapping quality of 5, and minimum length to retain a read after quality trimming of 25 bp. The mapping quality of 5 was used as a default value provided in the example config file by pipeline developers (https://github.com/StevenMicheletti/poolparty/blob/master/example/pp_align.config). It is worth noting that bwa aligner employed by PPalign module assigns mapping quality 0 to the alignments of reads mapped with equal scores to several different locations of repetitive sequences and all such alignments were filtered out with the default threshold. The PPalign module then uses BCFTOOLS (Li 2011) to call SNPs with a threshold SNP quality set to 20 and a minimum depth of coverage of 10 across the library for SNP retention. The reads were split by

barcode for each individual, normalized for read depth, and pooled for collection-level analysis of allele frequencies. Filters included minimum and maximum sequence coverage of 15 and 500 per population (here, populations refer to WBL versus WRL collections). The genome was trimmed to the first 100 scaffolds which were renamed to chromosomes 1 – 100 for plotting. Pairwise F_{ST} values between populations were calculated using PP.analyze module. In particular chromosomes 02, 05, 06, 08, 12, 14, 17, 24, 31, and 37 had regions of high divergence and we targeted 200 SNPs within these regions which were successfully developed as 47 SNPs for a GT-seq panel of markers (Table S3, dryad repository for forward/reverse primer sequences).

Chromosome 2 reassembly

We utilized BLASTn (ncbi-blast-2.11.0, Camacho et al. 2009) to align exons of the sea lamprey SV2C gene (NCBI Reference Sequence: XM_032969764.1) to the *Lampetra richardsoni* assembly. The alignment results showed that exons 1, 3-9 mapped to LPT_scaf_493, exon 2 to LPT_scaf_5926, exon 10 to LPT_scaf_8586, and only exons 11-13 to LPT_scaf_2, where the candidate SNP Lri2P16367064 is located. To investigate why gene sequence was scattered across multiple scaffolds, we mapped paired-end reads from Pool-seq data to the reference assembly of *Lampetra richardsoni* genome using bwa mem. A thorough examination of assembly gaps, mapped read fragments, and links between paired reads revealed points of misassembly within LPT_scaf_2 and proofs of linkage between the scaffold LPT_scaf_2 and three other scaffolds, enabling us to reconstruct the SV2C region on chromosome 2, as depicted in Figure S3. The supplementary file (*.agp) provides a formal description of the changes made to generate a new reference (referred to as Lri_SV2C_ref) with scaffold LPT_scaf_2_SV2C built from four original scaffolds harboring the SV2C gene.

To characterize F_{ST} values along the reassembled chromosome 2, we mapped reads from each Pool-seq sample to the Lri_SV2C_ref reference using bwa mem. We then employed bcftools (v.1.13, Li 2011) with the *mpileup* and *call* commands to identify variants from the alignments with mapping quality (MQ) > 30. For F_{ST} calculations, we selected sites that have aligned reads

from at least four individuals, variant quality > 20, minor allele frequency > 0.05 and mean depth values < 100. We computed F_{ST} values for each variant site using vcftools (v1.16, Danecek et al. 2011) with the --weir-fst-pop option, considering two populations: 24 WBL samples and 15 WRL samples. Calculated F_{ST} values were added to the bigwig file (selecting the highest F_{ST} value for sites with multiple variants) and visualized using JBrowse2 (v. 2.10.0, Diesh et al. 2023).

Genotyping of voucher specimen

Reads sequenced from each of the 39 individuals (sample b) of the Pool-seq experiment were mapped to the reference using bwa mem and filtered to only retain alignments with mapping quality 30 or higher. First, variants were called using bcftools commands mpileup and call with multiallelic calling model (v.1.13, Li 2011). The resulting vcf file was filtered to preserve only biallelic SNP sites with minimal allele frequency 0.1, coverage depth from the interval [5, 30] and defined for at least 27 individuals (16,261 SNPs in total). To ensure the confidence of heterozygous calls, only variants supported by at least two reads from each allele were retained for further analyses, resulting in a set of 835 sites with an average of 16% missing genotypes per individual (ranging from 1.4% to 70%).

Supplemental References

Camacho, C., Coulouris, G., Avagyan, V. et al. BLAST+: architecture and applications. BMC Bioinformatics 10, 421 (2009). https://doi.org/10.1186/1471-2105-10-421.

Danecek, P., Auton, A., Abecasis, G., Albers, C. A., Banks, E., DePristo, M. A., ... & 1000 Genomes Project Analysis Group. (2011). The variant call format and VCFtools. Bioinformatics, 27(15), 2156-2158.

Diesh, C., Stevens, G.J., Xie, P. et al. JBrowse 2: a modular genome browser with views of synteny and structural variation. Genome Biol 24, 74 (2023). https://doi.org/10.1186/s13059-023-02914-z

Lampman, R. (2018). Columbia Basin lamprey identification guide. Appendix 4.3 / L1 in Yakama Nation Pacific Lamprey Project 2017 Annual Progress Report (Cooperative Agreement No. R15AC00044 / Project No. 2008-470-00). Prepared for the U.S. Dept. of Interior, Bureau of Reclamation, Boise, ID, and U.S. Dept. of Energy, Bonneville Power Administration, Portland, OR. 3 pp.

Li, H., Handsaker, B., Wysoker, A., Fennell, T., Ruan, J., Homer, N., ... & 1000 Genome Project Data Processing Subgroup. (2009a). The sequence alignment/map format and SAMtools. bioinformatics, 25(16), 2078-2079..

Li H, and Durbin R. (2009b). Fast and accurate short read alignment with Burrows-Wheeler transform. Bioinformatics 25, 1754–1760. 10.1093/bioinformatics/btp324.

Li H. A statistical framework for SNP calling, mutation discovery, association mapping and population genetical parameter estimation from sequencing data. Bioinformatics. 2011 Nov 1;27(21):2987-93. doi: 10.1093/bioinformatics/btr509.

Micheletti, S. J., & Narum, S. R. (2018). Utility of pooled sequencing for association mapping in nonmodel organisms. Molecular Ecology Resources, 18(4), 825-837.

Quinlan AR, Hall IM. BEDTools: a flexible suite of utilities for comparing genomic features. Bioinformatics. 2010 Mar 15;26(6):841-2. doi: 10.1093/bioinformatics/btq033. Epub 2010 Jan 28. PMID: 20110278; PMCID: PMC2832824.

Roach, M.J., Schmidt, S.A. & Borneman, A.R. Purge Haplotigs: allelic contig reassignment for third-gen diploid genome assemblies. BMC Bioinformatics 19, 460 (2018). https://doi.org/10.1186/s12859-018-2485-7

Sweet, D., Lorente, M., Valenzuela, A., Lorente, J., & Alvarez, J. C. (1996). Increasing DNA extraction yield from saliva stains with a modified Chelex method. Forensic science international, 83(3), 167-177.

Weisenfeld, N. I., Kumar, V., Shah, P., Church, D. M., & Jaffe, D. B. (2017). Direct determination of diploid genome sequences. Genome Research, 27(5), 757-767.

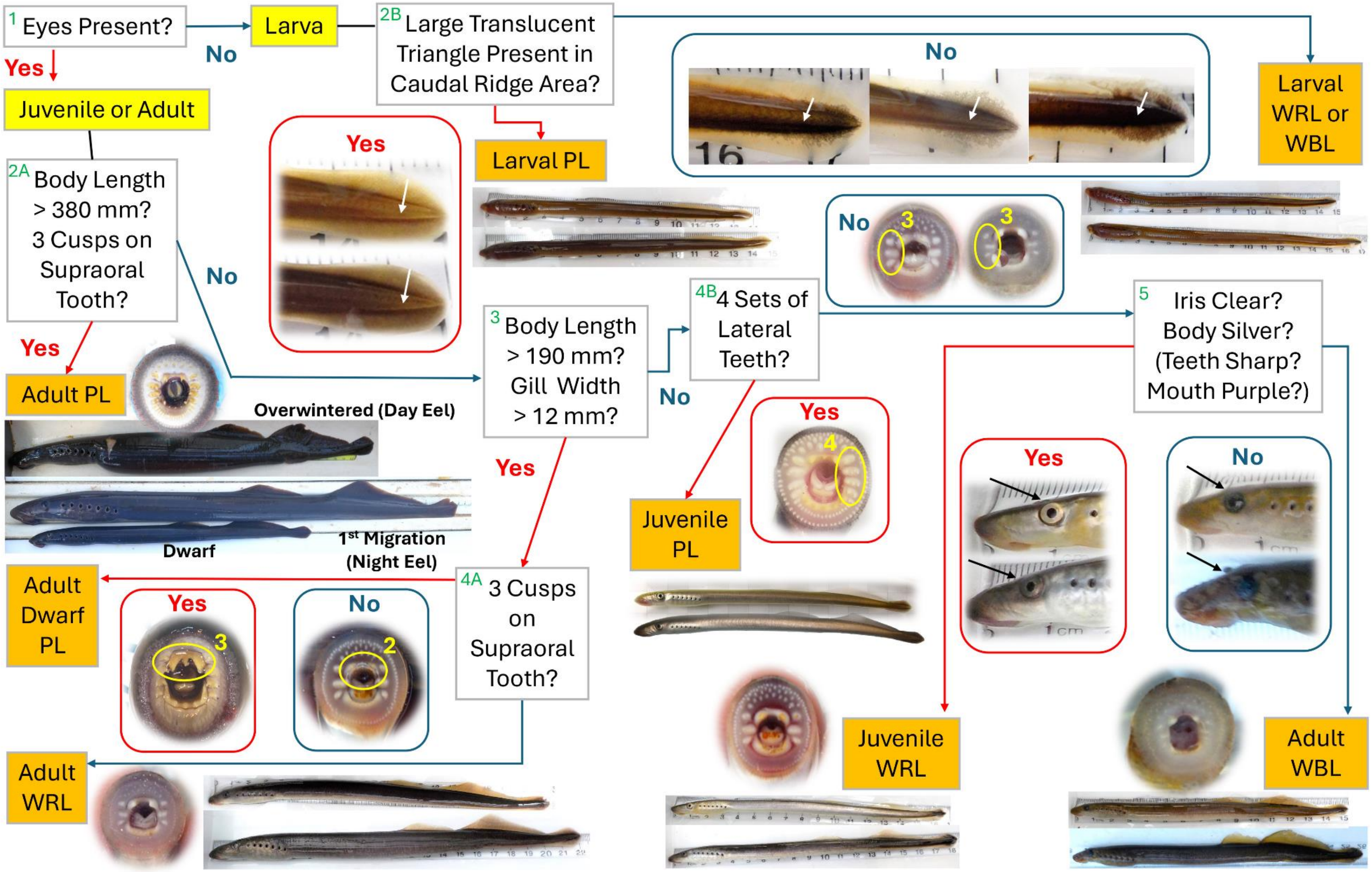
1 Eyes Present?
Yes
No
Larva
2B Large Translucent Triangle Present in Caudal Ridge Area?
Yes
No
Larval PL
Larval WRL or WBL
Juvenile or Adult
2A Body Length > 380 mm? 3 Cusps on Supraoral Tooth?
Yes
No
Adult PL
Overwintered (Day Eel)
Dwarf
1st Migration (Night Eel)
3 Body Length > 190 mm? Gill Width > 12 mm?
Yes
No
4A 3 Cusps on Supraoral Tooth?
Yes
No
3
2
Adult Dwarf PL
Adult WRL
4B 4 Sets of Lateral Teeth?
Yes
No
4
3
Juvenile PL
5 Iris Clear? Body Silver? (Teeth Sharp? Mouth Purple?)
Yes
No
Juvenile WRL
Adult WBL

Figure S1. Dichotomous key to guide morphological identification of the three focal lampreys (WBL, WRL, and Pacific Lamprey) in NE Pacific tributaries. The guide uses different traits including total body length, dentition and tail pigmentation patterns to ID the focal lamprey taxa and categorize by life stage (adult, juvenile, and larva). "Overwintered (Day Eel)", "1st migration (Night Eel)", and "Dwarf" are all terms that have been applied to describe a range of variation observed among Pacific Lamprey adults (Clemens et al. 2019).

Figure S2. Photo documentation of morphologically identified WBL and WRL that were used for whole genome resequencing dataset.

Western River Lamprey (N=7) - Adult Life Stage (Returning from the Sea)

1. 198 mm, immature adult, Class B2 tail, medium iris & dark body, slightly sharp teeth (ID=B12, 5/6/2019); Decision Tree Pathway: 1-YES-2A-NO-3-YES-4A-NO

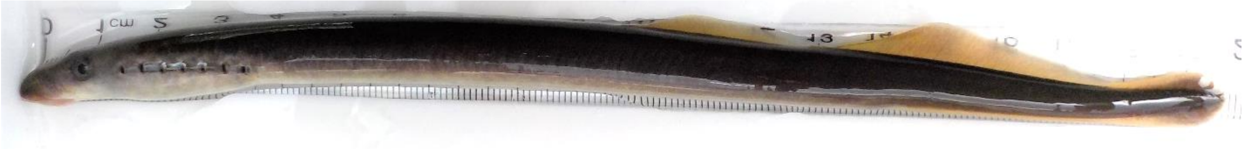

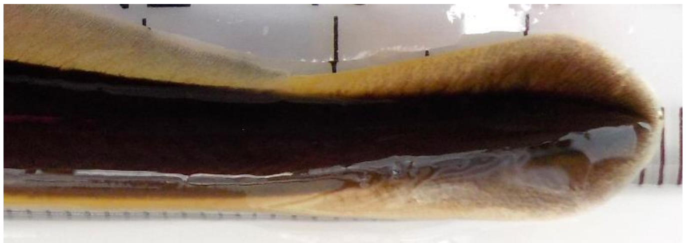

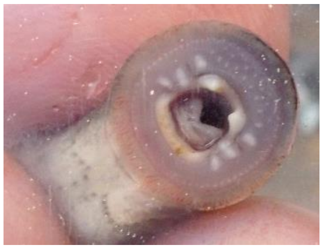

1. 220 mm, male adult, Class B2 tail, medium iris & dark body, slightly sharp teeth (ID=B18, 5/20/2019); Decision Tree Pathway: 1-YES-2A-NO-3-YES-4A-NO

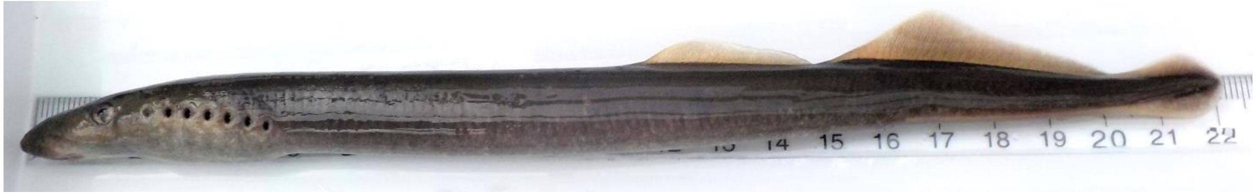

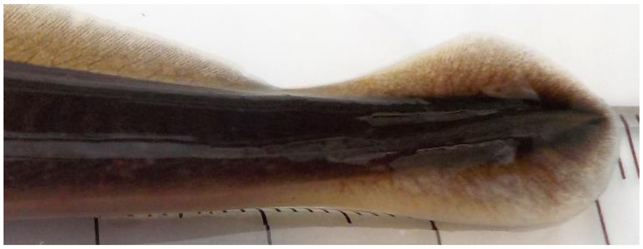

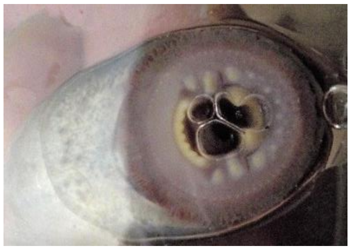

2. 217 mm, female adult, Class B1 tail, medium iris & dark body, dull teeth (ID=B21, 5/21/2019); Decision Tree Pathway: 1-YES-2A-NO-3-YES-4A-NO

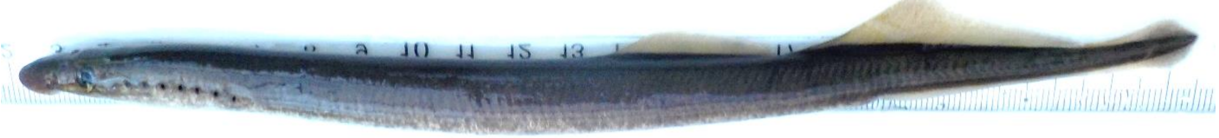

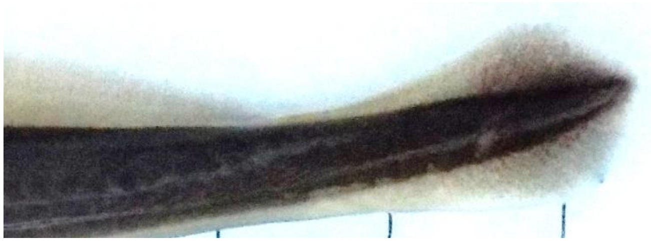
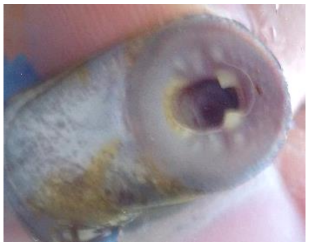

3. 200 mm, female adult (ripe with eggs), Class B1 tail, dark iris & dark body, dull teeth (ID=B22, 6/3/2019); Decision Tree Pathway: 1-YES-2A-NO-3-YES-4A-NO

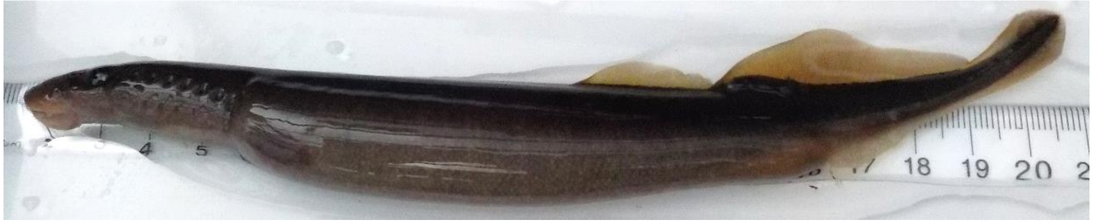

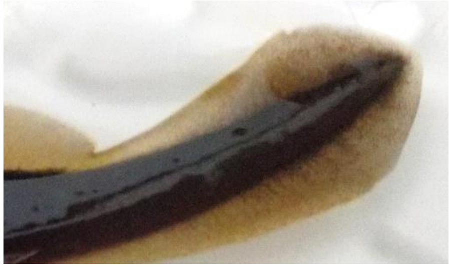
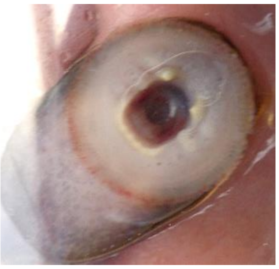
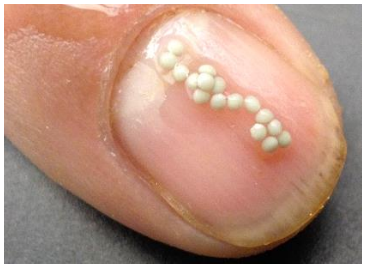

4. 205 mm, male adult, Class B2 tail, dark iris & dark body, dull teeth (ID=B25, 6/5/2019); Decision Tree Pathway: 1-YES-2A-NO-3-YES-4A-NO

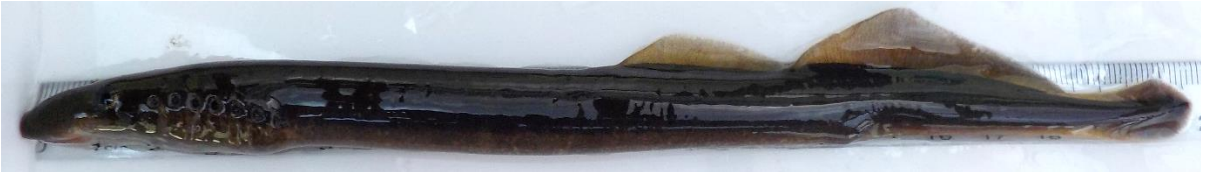

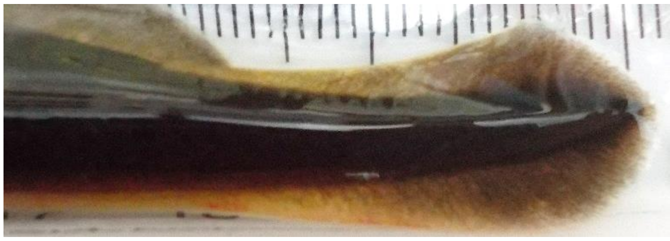
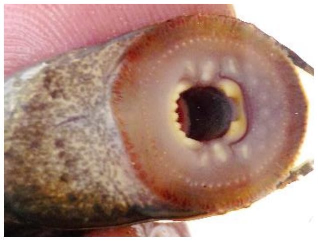

5. 200 mm, adult, tail photo missing, medium iris & dark body, dull teeth (ID=B28, 6/6/2019); Decision Tree Pathway: 1-YES-2A-NO-3-YES-4A-NO

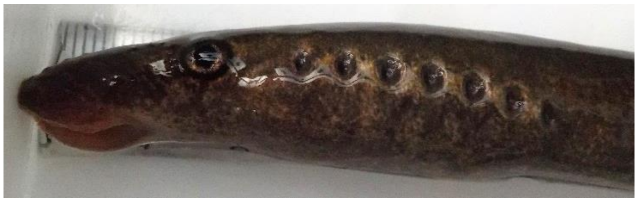

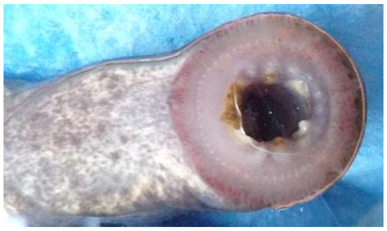

6. 202 mm, female adult, Class B1 tail, medium iris & dark body, dull teeth (ID=B23, 6/3/2019); Decision Tree Pathway: 1-YES-2A-NO-3-YES-4A-NO

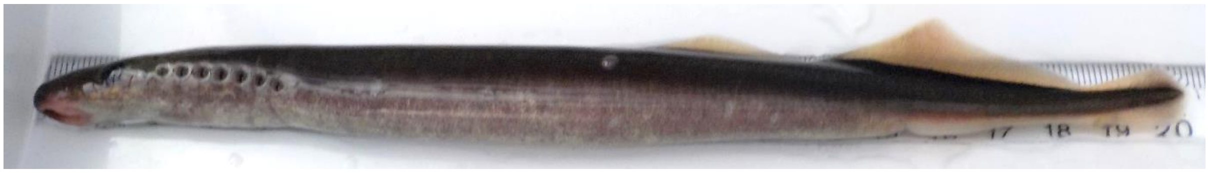

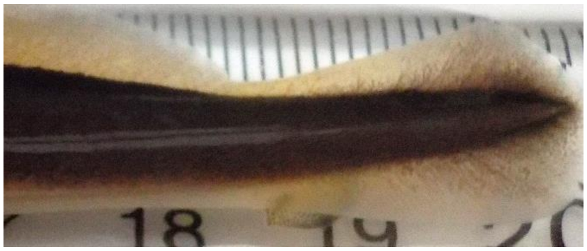 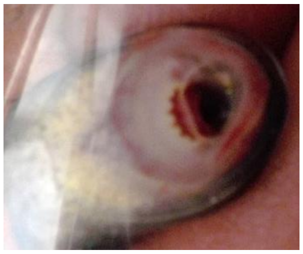

<u>Western River Lamprey (N=11) - Juvenile Life Stage (Migrating out to the Sea)</u>

1. 156 mm, juvenile, Class B1/2 tail, light iris, silver body, sharp teeth (ID=S2, 5/2/2019); Decision Tree Pathway: 1-YES-2A-NO-3-No-4B-No-5-YES

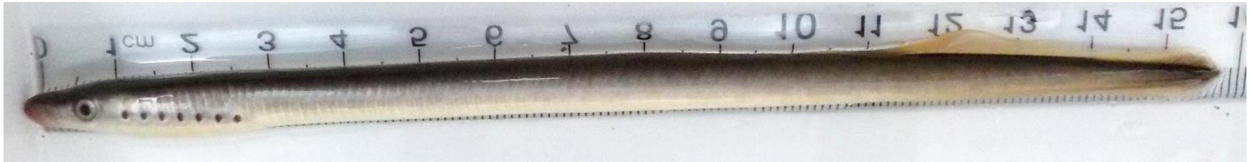

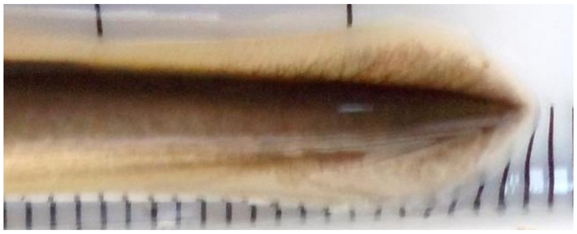
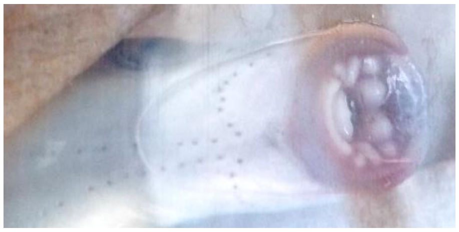

2. 183 mm, juvenile, Class B2 tail, light iris, silver body, slightly sharp teeth (ID=S3, 5/2/2019); Decision Tree Pathway: 1-YES-2A-NO-3-No-4B-No-5-YES

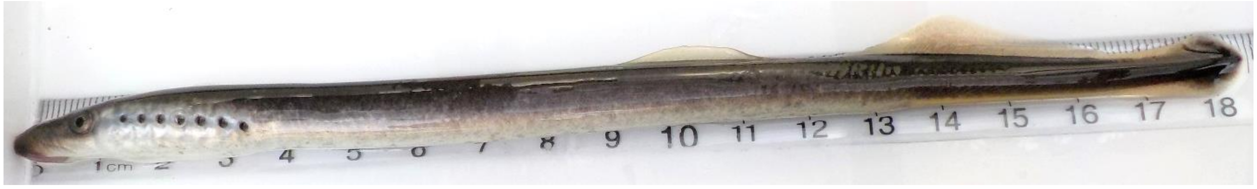

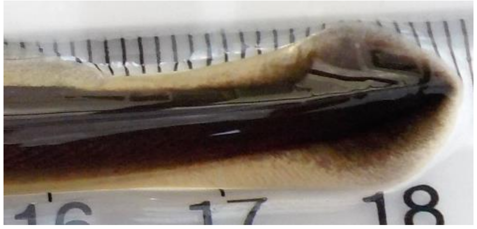
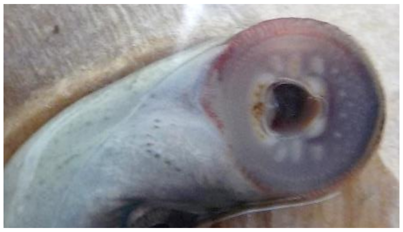

3. 161 mm, juvenile, Class B1 tail, light iris, silver body, sharp teeth (ID=S4, 5/6/2019); Decision Tree Pathway: 1-YES-2A-NO-3-No-4B-No-5-YES

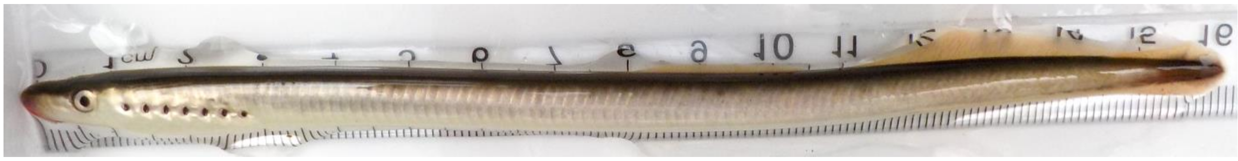

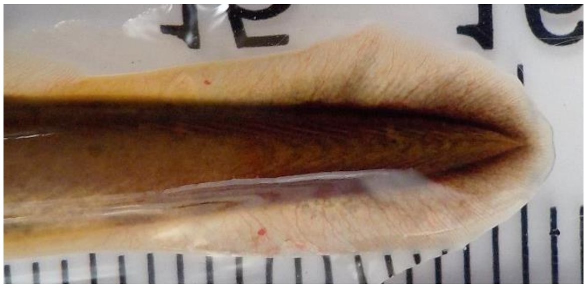
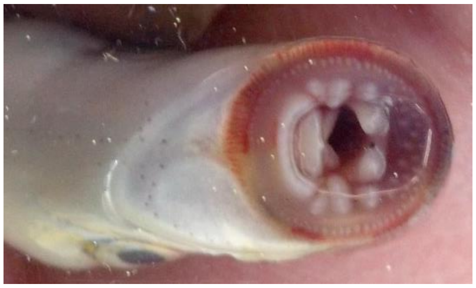

4. 149 mm, juvenile, Class B1 tail, light iris, silver body, sharp teeth (ID=S5, 5/6/2019); Decision Tree Pathway: 1-YES-2A-NO-3-No-4B-No-5-YES

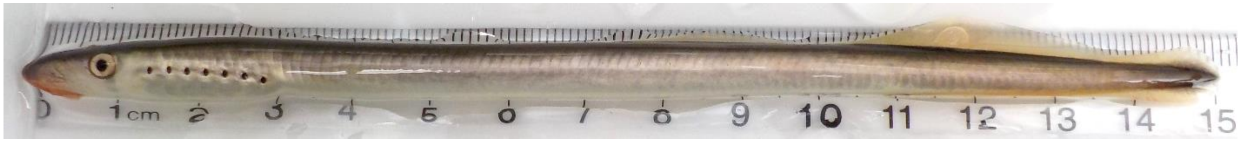

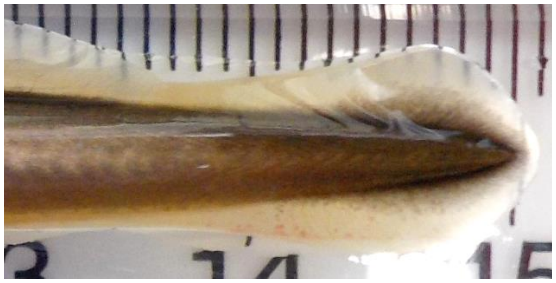

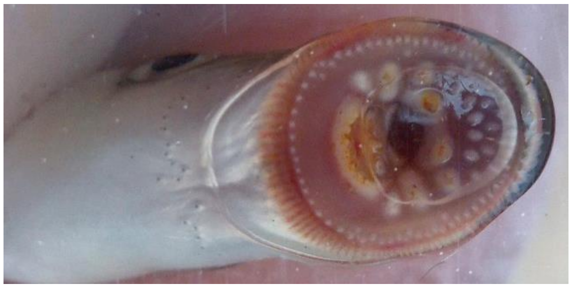

5. 147 mm, juvenile, Class B1/2 (intermediate) tail, light iris, silver body, sharp teeth (ID=S6, 5/6/2019); Decision Tree Pathway: 1-YES-2A-NO-3-No-4B-No-5-YES

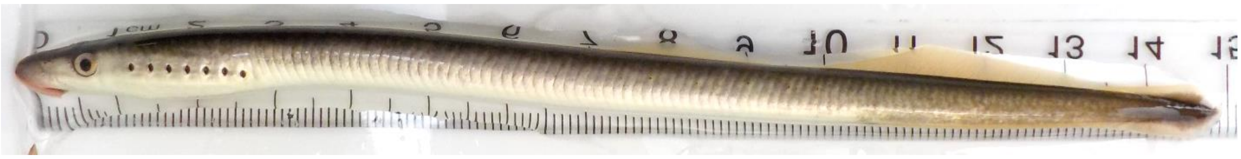

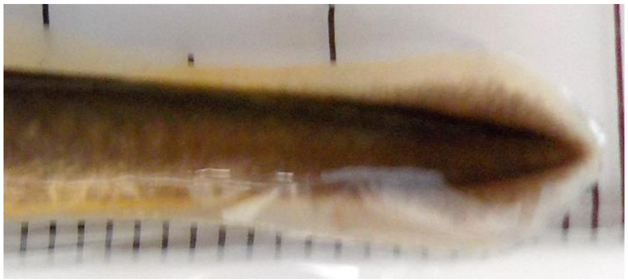

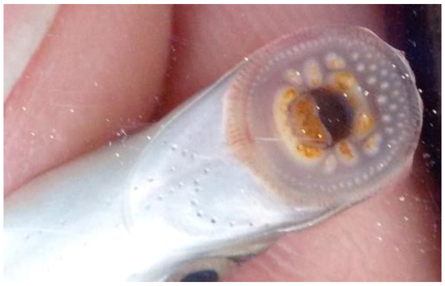

6. 157 mm, juvenile, Class B2 tail, light iris, silver body, sharp teeth (ID=S9, 5/7/2019); Decision Tree Pathway: 1-YES-2A-NO-3-No-4B-No-5-YES

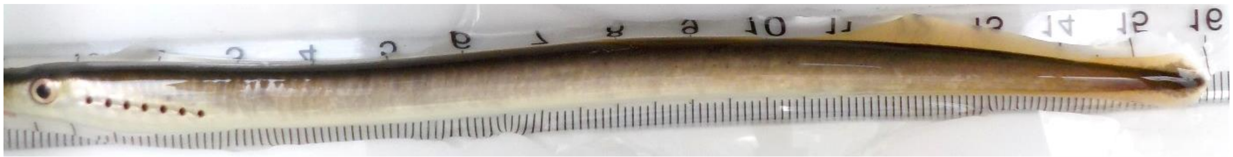

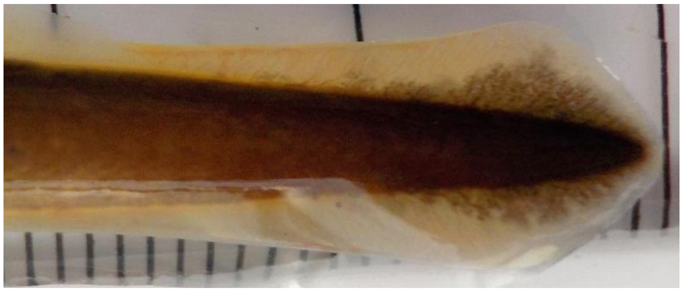

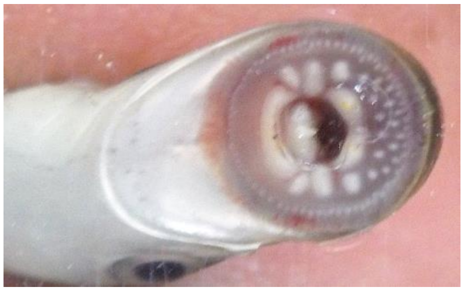

7. 158 mm, juvenile, Class B2 tail, light iris, silver body, sharp teeth (ID=S13, 5/7/2019); Decision Tree Pathway: 1-YES-2A-NO-3-No-4B-No-5-YES

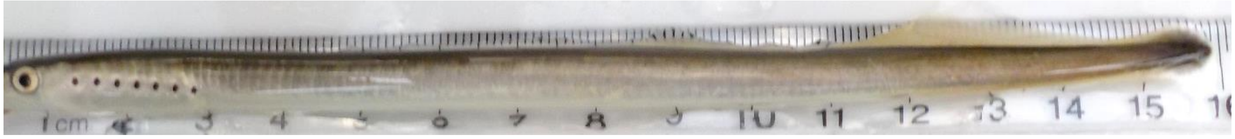

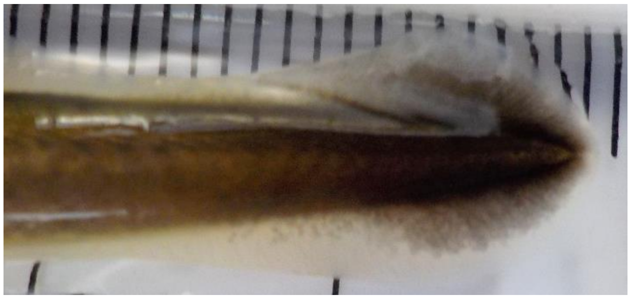
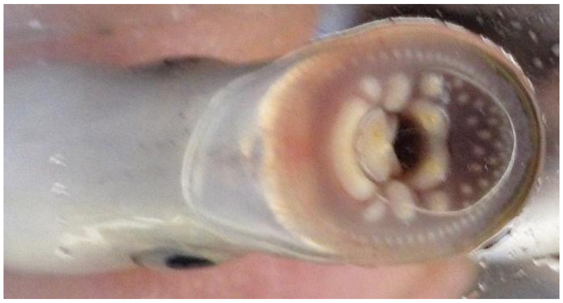

8. 171 mm, juvenile, Class B2 tail, light iris, silver body, sharp teeth (ID=S20, 5/12/2019); Decision Tree Pathway: 1-YES-2A-NO-3-No-4B-No-5-YES

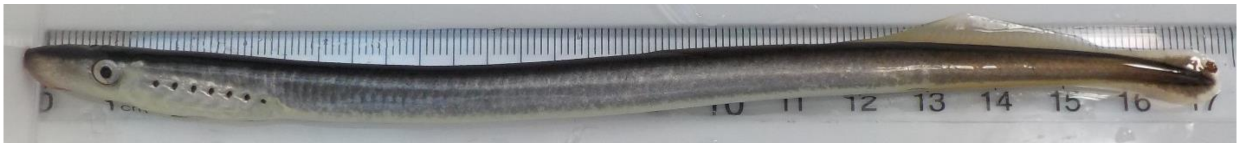

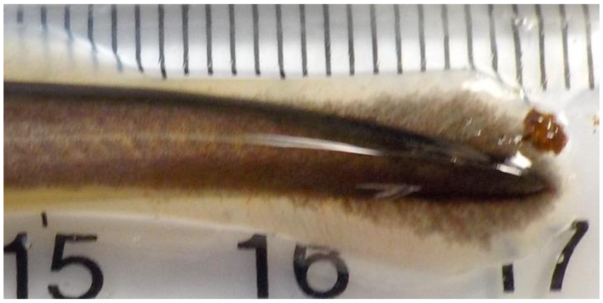
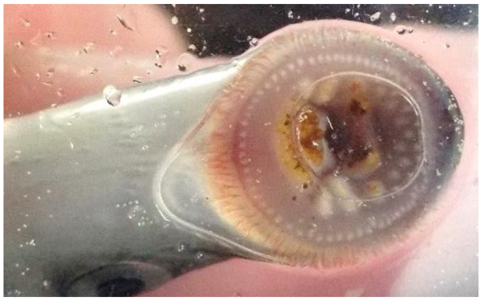

9. 166 mm, juvenile, Class C tail, light iris, silver body, sharp teeth (ID=S19, 5/8/2019,) Decision Tree Pathway: 1-YES-2A-NO-3-No-4B-No-5-YES

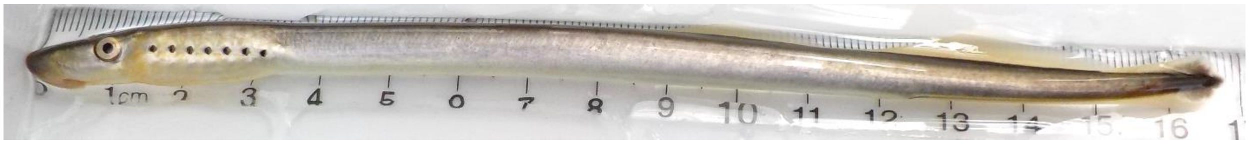

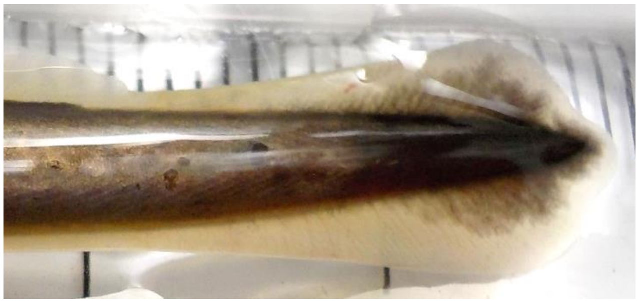
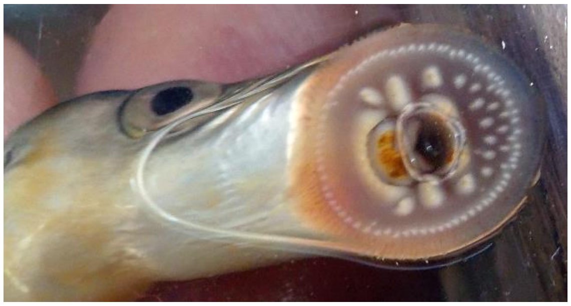

10. 157 mm, juvenile, Class B1 tail, light iris, silver body, sharp teeth (ID=S7, 5/6/2019); Decision Tree Pathway: 1-YES-2A-NO-3-No-4B-No-5-YES

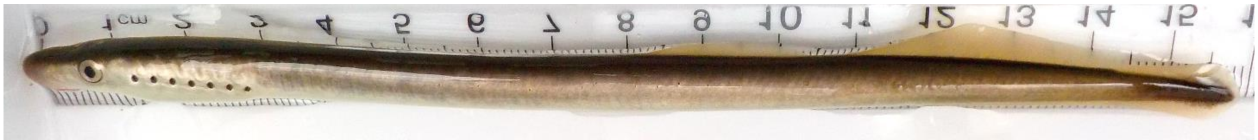

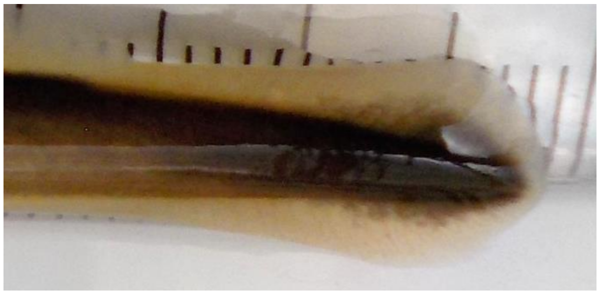

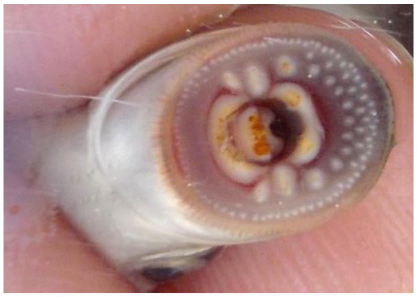

11. 157 mm, juvenile, Class B2 tail, light iris, silver body, sharp teeth (ID=S8, 5/6/2019); Decision Tree Pathway: 1-YES-2A-NO-3-No-4B-No-5-YES

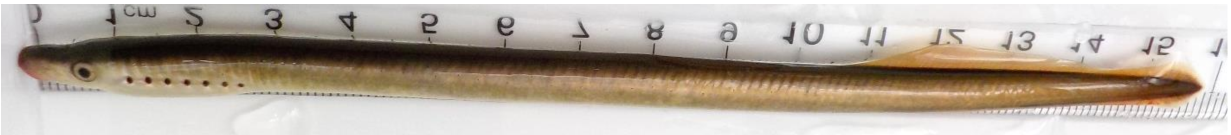

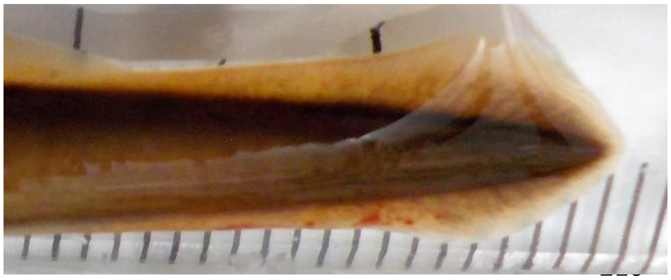

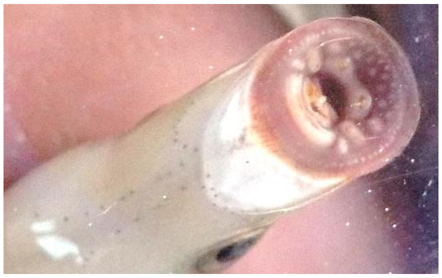

Western Brook Lamprey (N=29) - Adult Life Stage (Freshwater Resident)

1145 mm, immature adult, Class B1 tail, dark iris & body, dull teeth (ID=B2, 5/2/2019); Decision Tree Pathway: 1-YES-2A-NO-3-No-4B-No-5-NO

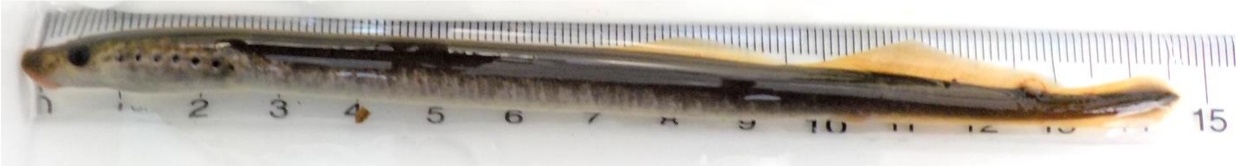

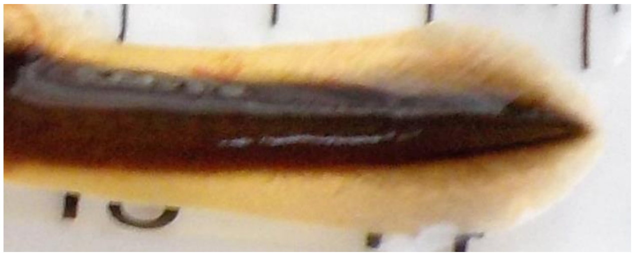 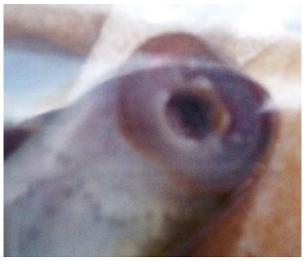

2. 166 mm, female adult, Class B2 tail, dark iris & body, dull teeth (ID=B3, 5/2/2019); Decision Tree Pathway: 1-YES-2A-NO-3-No-4B-No-5-NO

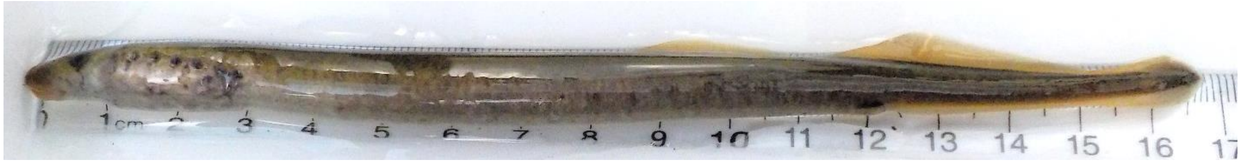

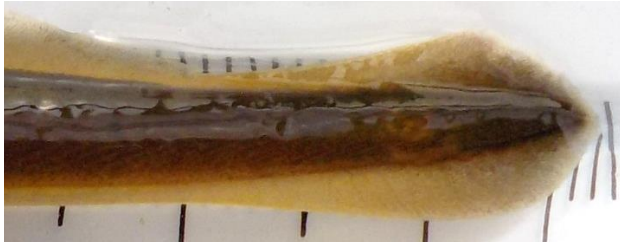 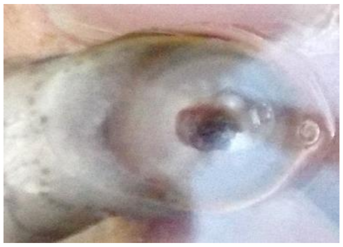

3. 177 mm, male adult, Class B2 tail, dark iris & body, dull teeth (ID=B5, 5/2/2019); Decision Tree Pathway: 1-YES-2A-NO-3-No-4B-No-5-NO

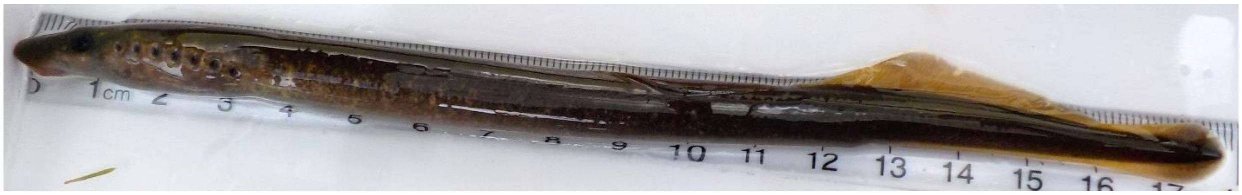

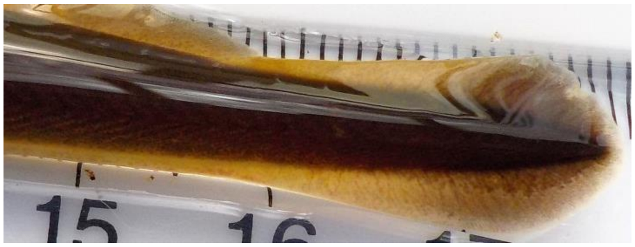 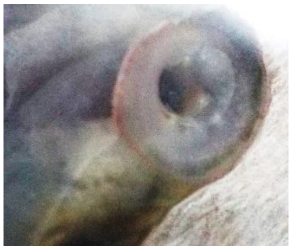

4. 137 mm, female adult, Class B1 tail, dark iris & body, dull teeth (ID=B6, 5/2/2019); Decision Tree Pathway: 1-YES-2A-NO-3-No-4B-No-5-NO

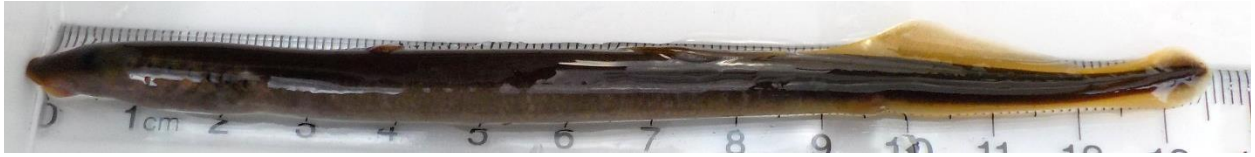

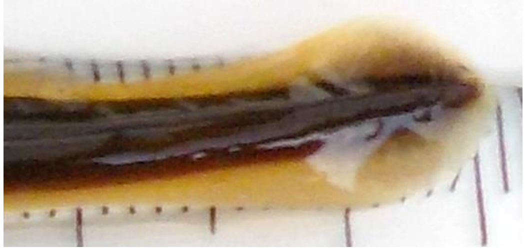

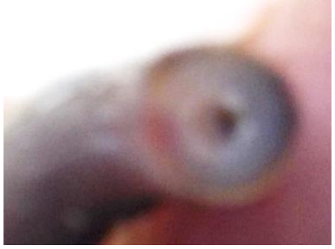

5. 167 mm, female adult, Class B2 tail, dark iris & body, dull teeth (ID=B10, 5/6/2019); Decision Tree Pathway: 1-YES-2A-NO-3-No-4B-No-5-NO

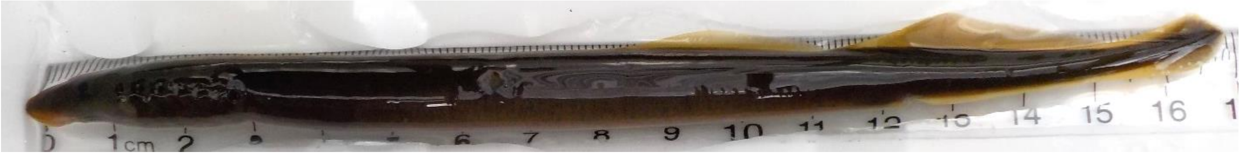

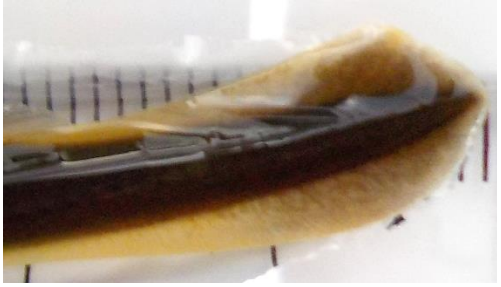

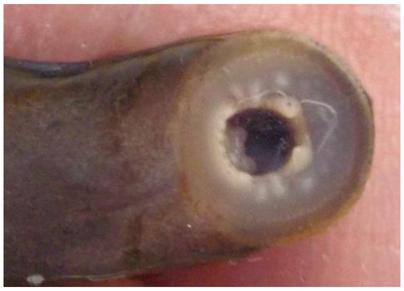

6. 144 mm, male adult, Class B1 tail, dark iris & body, dull teeth (ID=B16, 5/8/2019); Decision Tree Pathway: 1-YES-2A-NO-3-No-4B-No-5-NO

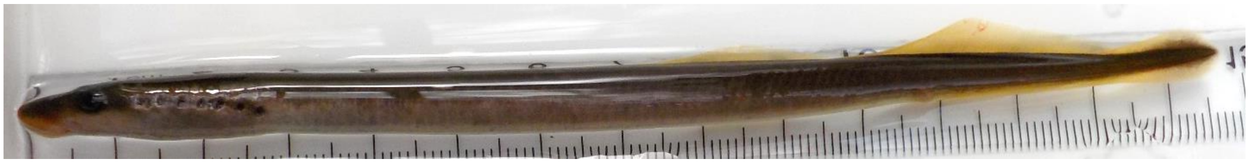

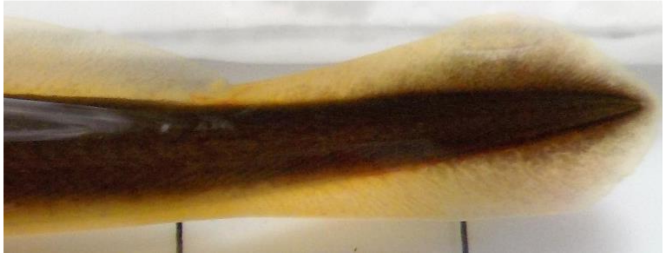

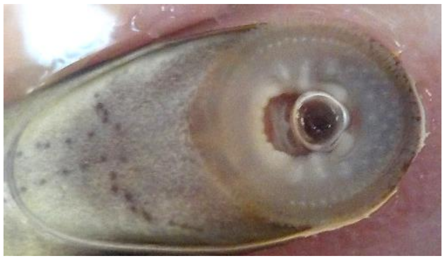

7. 180 mm, male adult, Class B2 tail, dark iris & body, dull teeth (ID=B20, 5/21/2019); Decision Tree Pathway: 1-YES-2A-NO-3-No-4B-No-5-NO

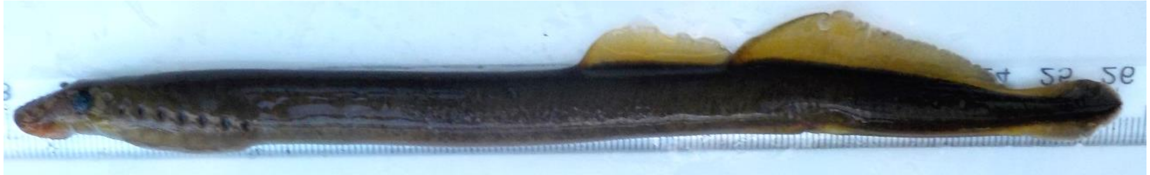

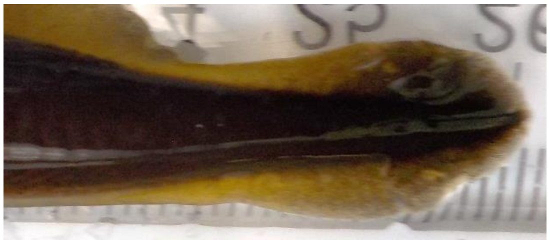 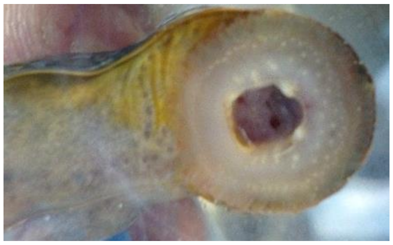

8. 158 mm, male adult, Class A tail, medium iris & gold body (unusual), dull teeth (ID=B24, 6/3/2019); Decision Tree Pathway: 1-YES-2A-NO-3-No-4B-No-5-NO

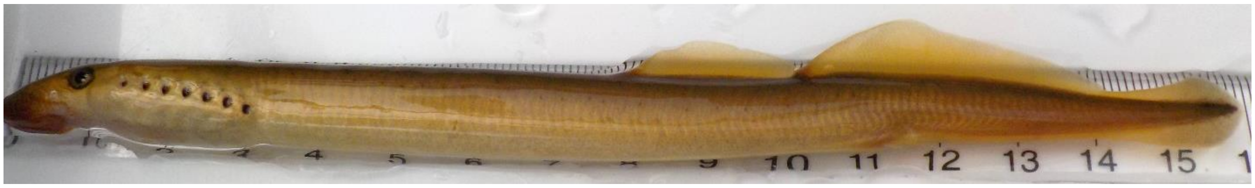

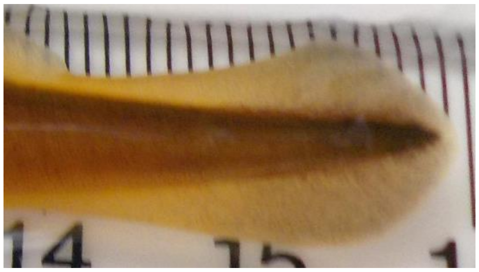 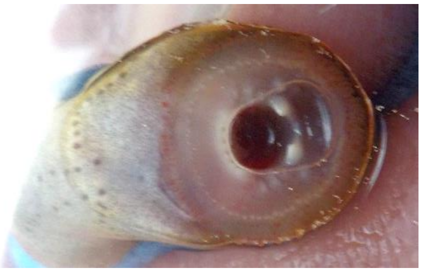

9. 127 mm, female adult, Class A tail, dark iris & body, teeth photo missing (ID=B27, 6/6/2019); Decision Tree Pathway: 1-YES-2A-NO-3-No-4B-No-5-NO

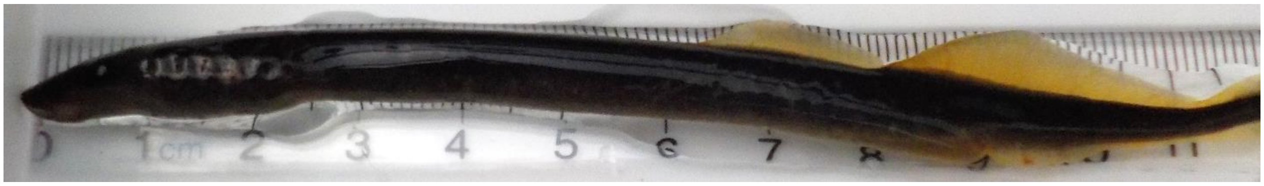

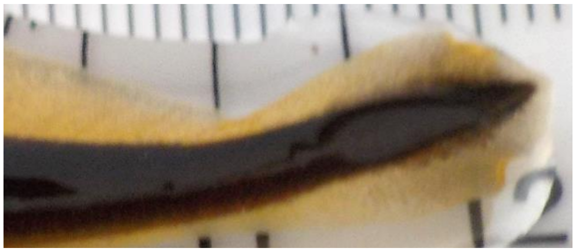

10. 175 mm, immature adult, Class B2 tail, dark iris & body, dull teeth (ID=S1, 5/2/2019); Decision Tree Pathway: 1-YES-2A-NO-3-No-4B-No-5-NO

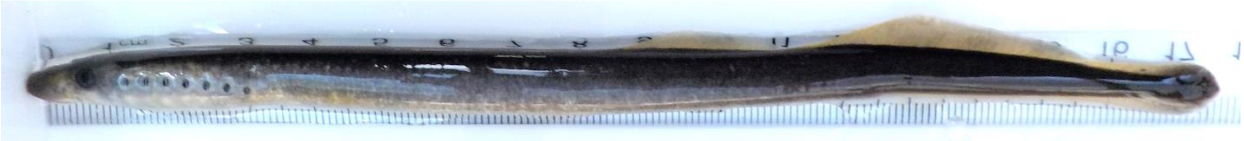

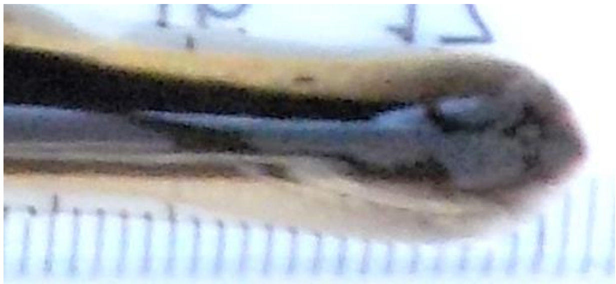

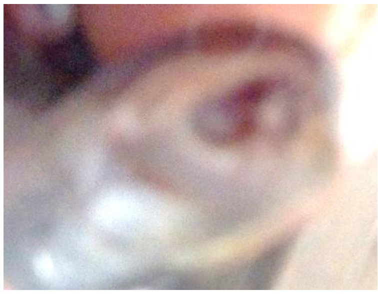

11. 150 mm, female, Class B2 tail, dark iris & body, dull teeth (ID=S11, 5/7/2019); Decision Tree Pathway: 1-YES-2A-NO-3-No-4B-No-5-NO

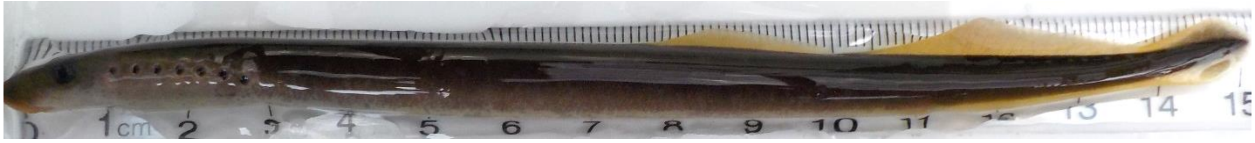

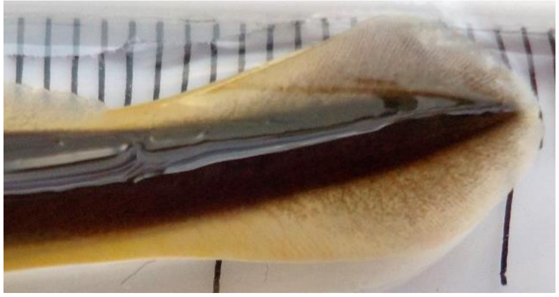

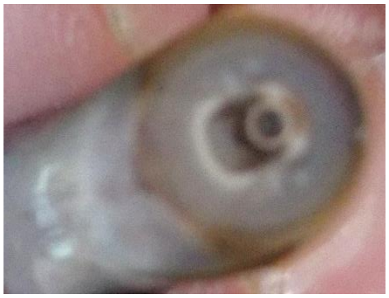

12. 157 mm, male adult, Class C tail, dark iris & body, dull teeth (ID=S12, 5/7/2019); Decision Tree Pathway: 1-YES-2A-NO-3-No-4B-No-5-NO

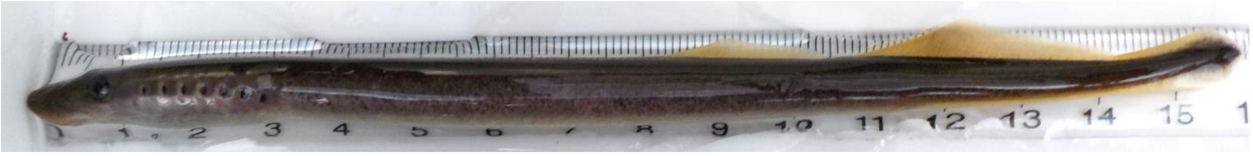

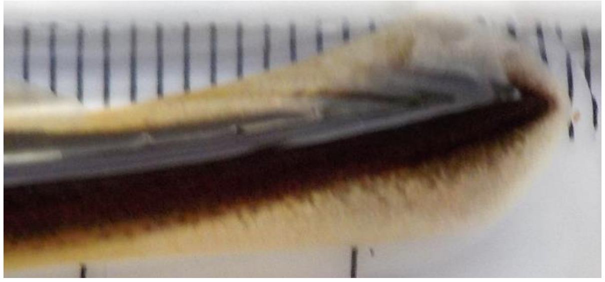

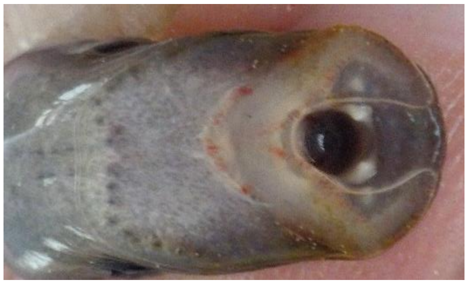

13. 151 mm, male adult, Class B1 tail, dark iris & body, dull teeth (ID=S14, 5/7/2019); Decision Tree Pathway: 1-YES-2A-NO-3-No-4B-No-5-NO

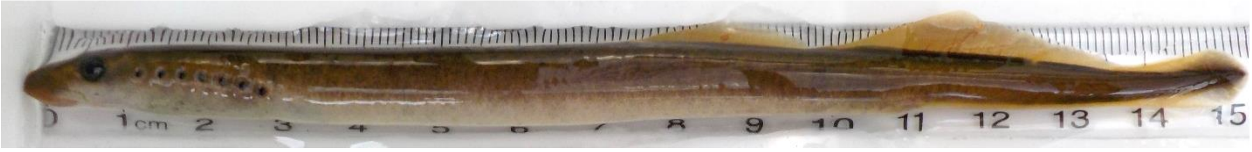

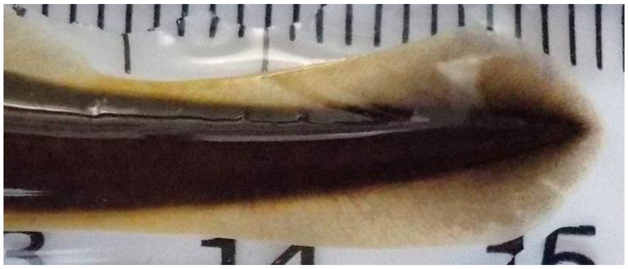
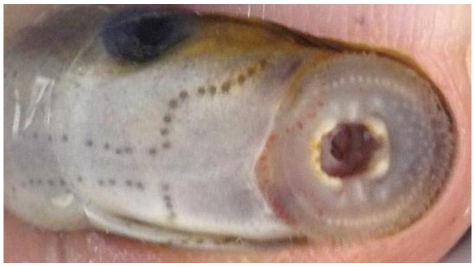

14. 154 mm, male adult, Class A tail, dark iris & body, dull teeth (ID=S15, 5/7/2019); Decision Tree Pathway: 1-YES-2A-NO-3-No-4B-No-5-NO

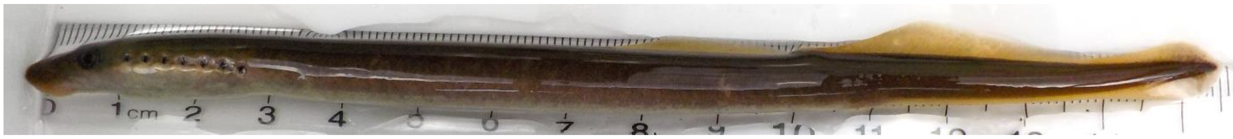

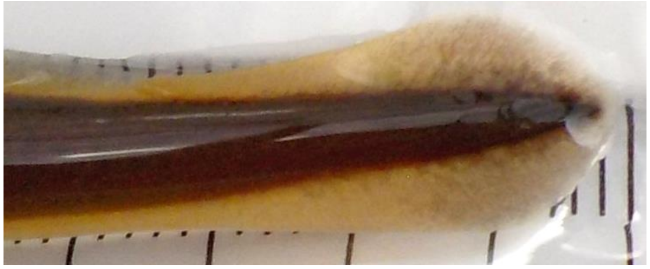
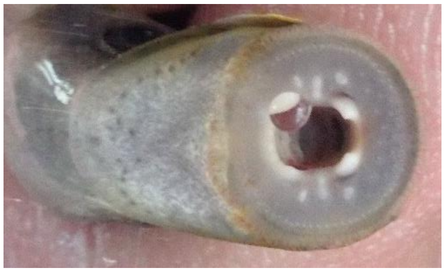

2. 152 mm, male adult, Class C tail, medium iris & dark body, slightly sharp teeth (ID=S18, 5/7/2019); Decision Tree Pathway: 1-YES-2A-NO-3-No-4B-No-5-NO

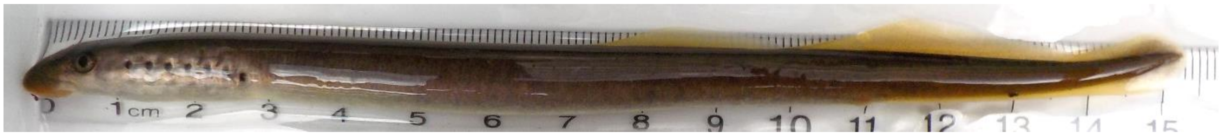

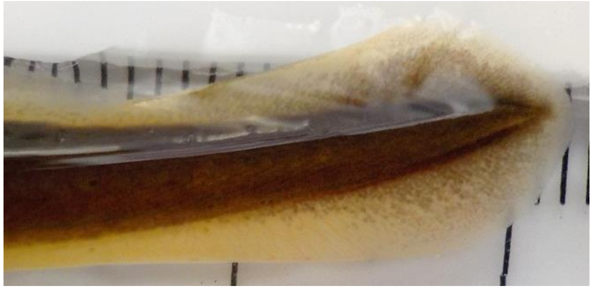
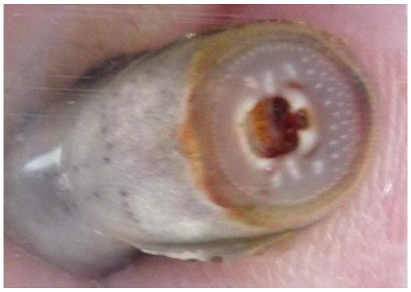

15. 138 mm, female adult, Class B2 tail, dark iris & body, dull teeth (ID=B1, 5/2/2019); Decision Tree Pathway: 1-YES-2A-NO-3-No-4B-No-5-NO

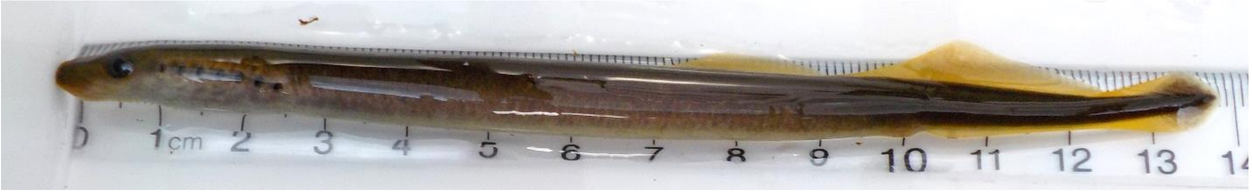

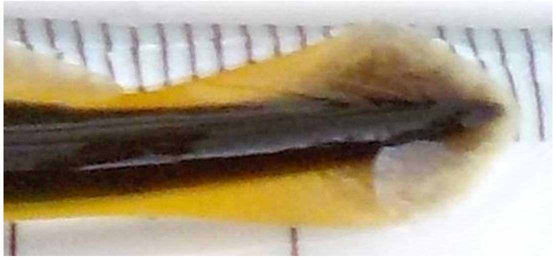 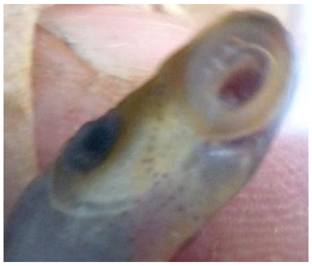

16. 184 mm, female adult, Class C tail, dark iris & lighter body, dull teeth (ID=B11, 5/6/2019); Decision Tree Pathway: 1-YES-2A-NO-3-No-4B-No-5-NO

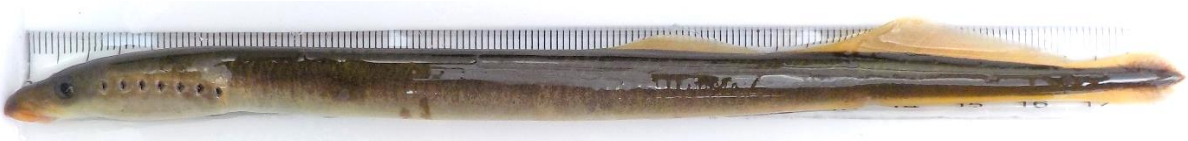

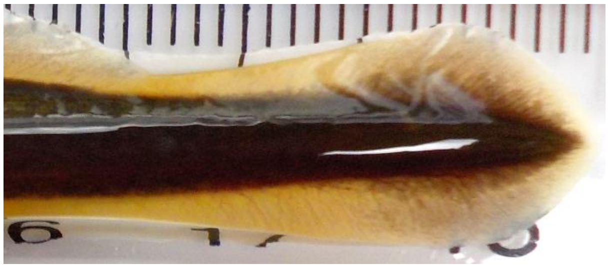 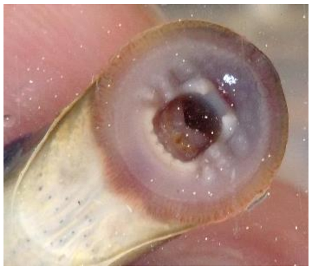

3. 149 mm, male adult, Class B2 tail, dark iris & body, slightly sharper teeth (ID=B29, 6/6/2019); Decision Tree Pathway: 1-YES-2A-NO-3-No-4B-No-5-NO

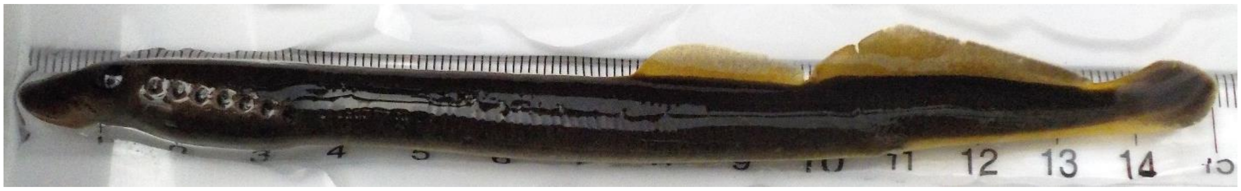

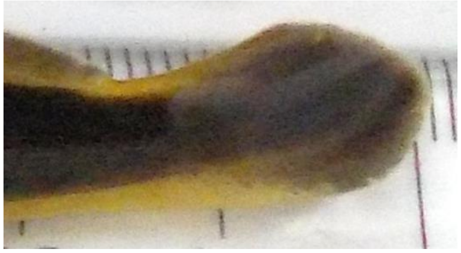 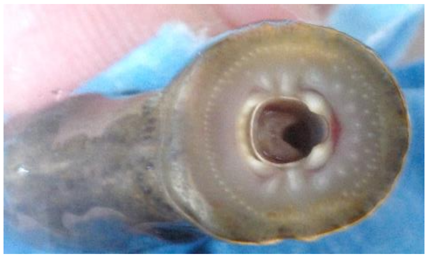

4. 150 mm, male adult, Class B2 tail, dark iris & body, slightly sharp teeth (ID=S17, 5/7/2019); Decision Tree Pathway: 1-YES-2A-NO-3-No-4B-No-5-NO

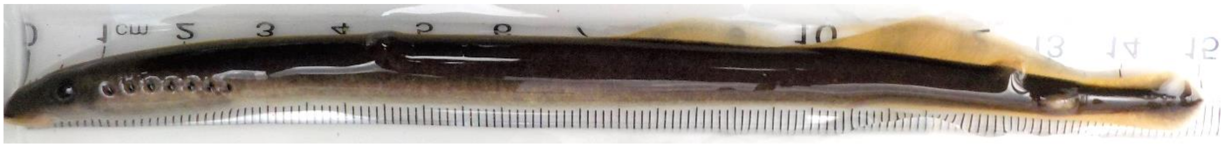

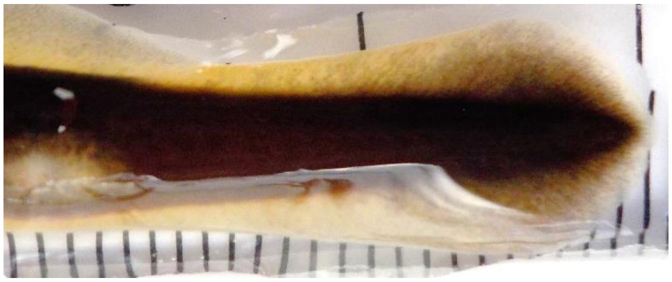 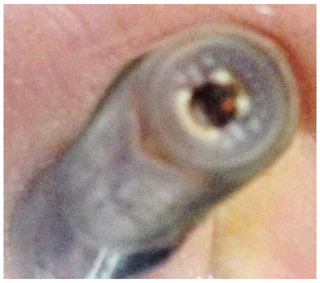

5. 151 mm, female adult, Class B2 tail, dark iris & body, dull teeth (ID=B14, 5/7/2019); Decision Tree Pathway: 1-YES-2A-NO-3-No-4B-No-5-NO

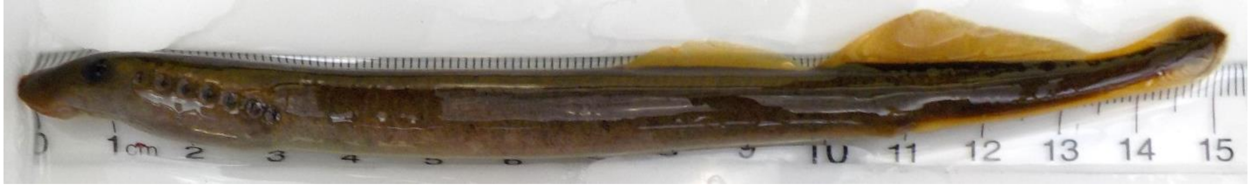

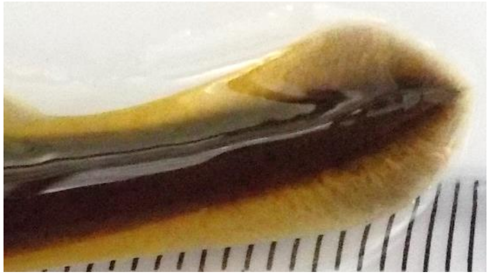 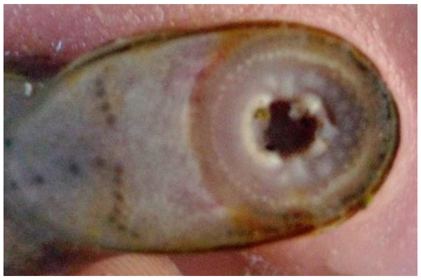

6. 152 mm, female adult, tail photo at angle, dark iris & body, dull teeth (ID=B17, 5/20/2019, NA); Decision Tree Pathway: 1-YES-2A-NO-3-No-4B-No-5-NO

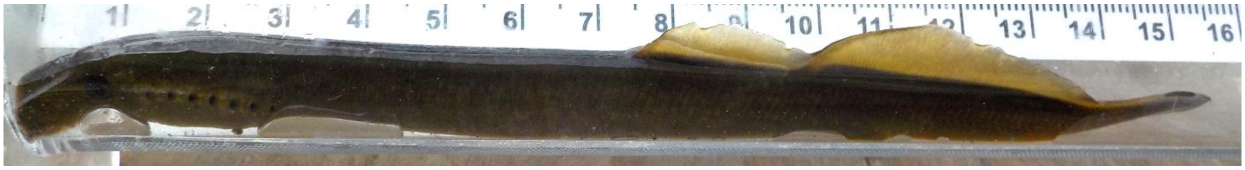

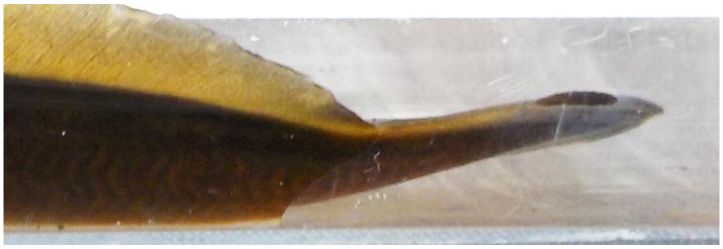

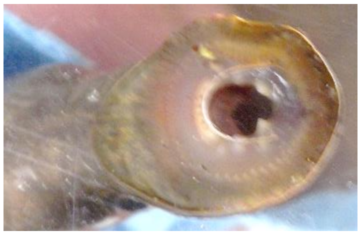

7. 160 mm, male adult, Class B2 tail, dark iris & body, dull teeth (ID=B4, 5/2/2019); Decision Tree Pathway: 1-YES-2A-NO-3-No-4B-No-5-NO

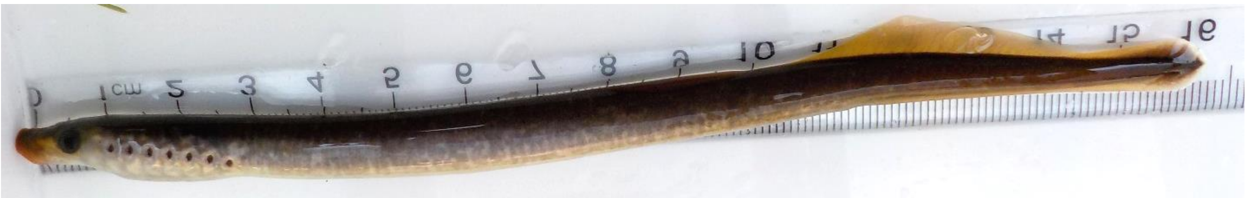

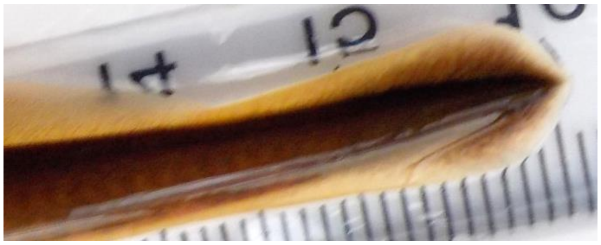

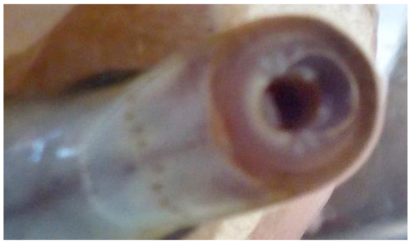

8. 155 mm, immature adult, Class A tail, dark iris & lighter body, slightly sharper teeth (ID=B7, 5/2/2019); Decision Tree Pathway: 1-YES-2A-NO-3-No-4B-No-5-NO

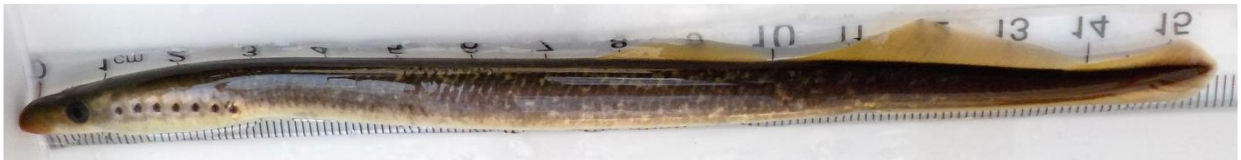

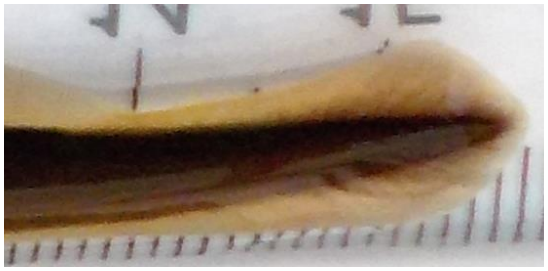

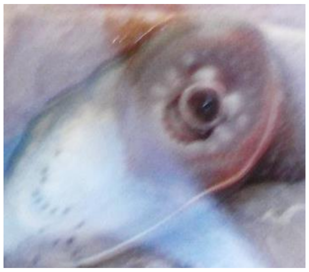

9. 157 mm, immature adult, Class B2 tail, dark iris & lighter body, slightly sharper teeth (ID=B8, 5/2/2019); Decision Tree Pathway: 1-YES-2A-NO-3-No-4B-No-5-NO

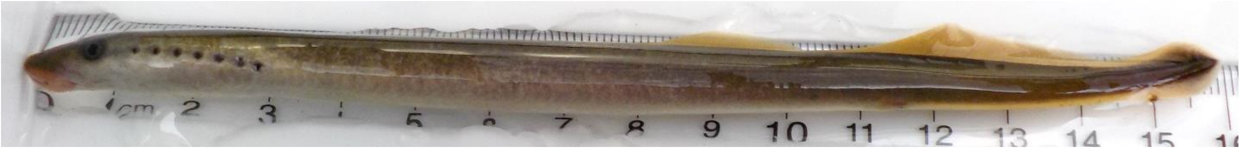

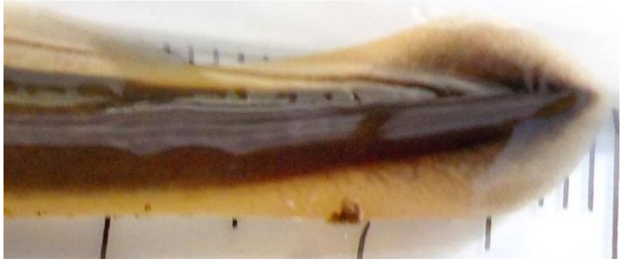 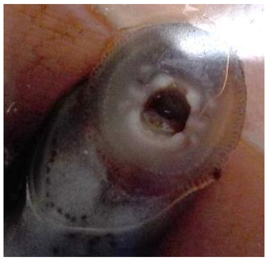

10. 155 mm, female adult, Class B2 tail, dark iris & body, dull teeth (ID=B9, 5/2/2019); Decision Tree Pathway: 1-YES-2A-NO-3-No-4B-No-5-NO

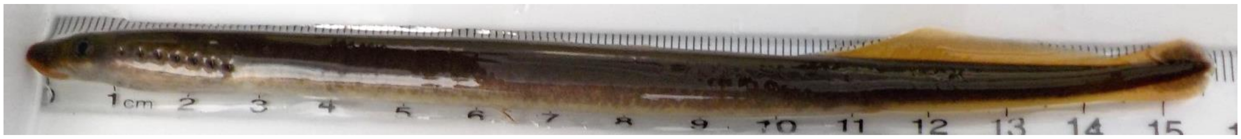

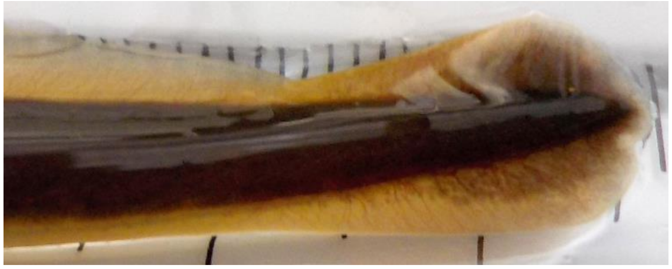 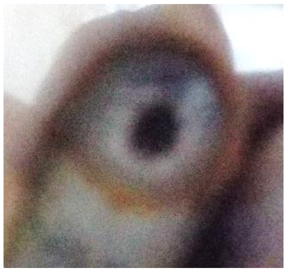

11. 174 mm, male adult, Class B2 tail, dark iris & body, dull teeth (ID=B13, 5/6/2019); Decision Tree Pathway: 1-YES-2A-NO-3-No-4B-No-5-NO

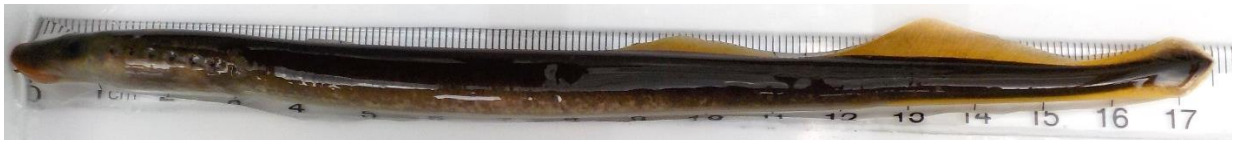

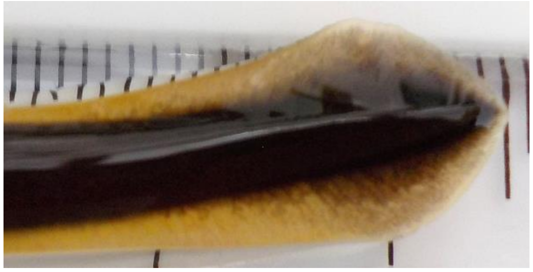 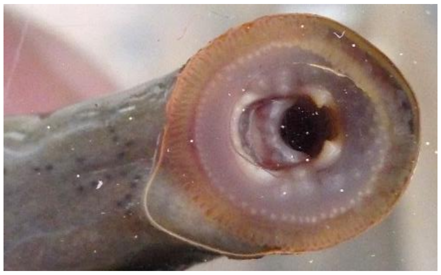

12. 143 mm, male adult, Class B1 tail, dark iris & body, dull teeth (ID=B15, 5/8/2019); Decision Tree Pathway: 1-YES-2A-NO-3-No-4B-No-5-NO

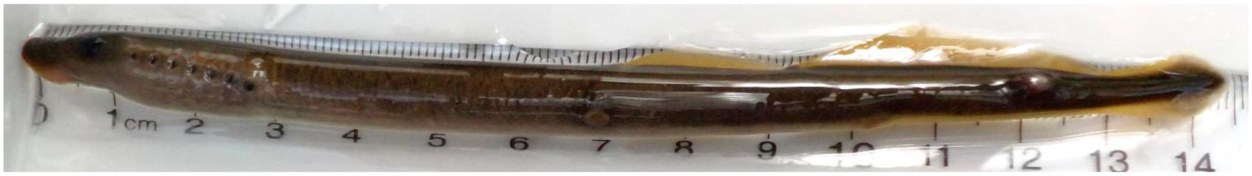

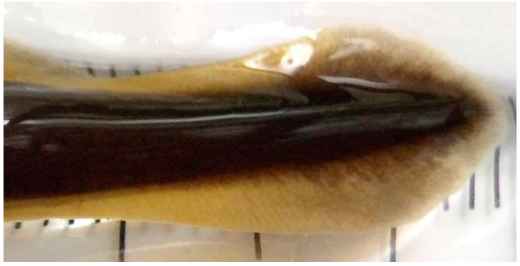 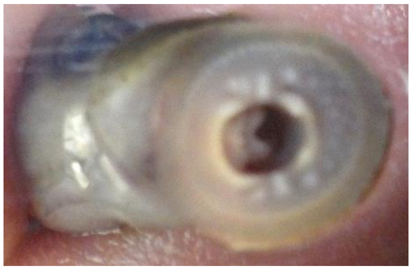

13. 130 mm, male adult, Class B1/2 (intermediate) tail, dark iris & body, dull teeth (ID=B19, 5/20/2019); Decision Tree Pathway: 1-YES-2A-NO-3-No-4B-No-5-NO

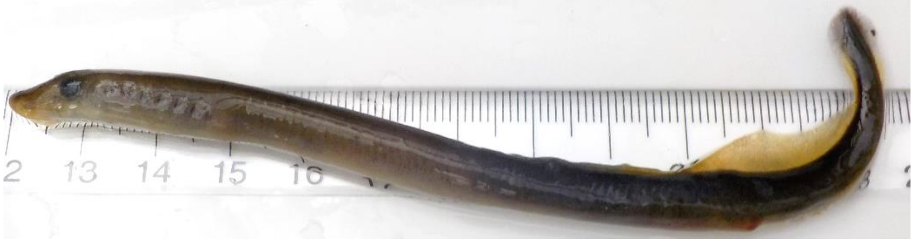

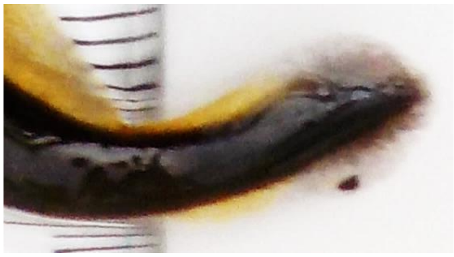 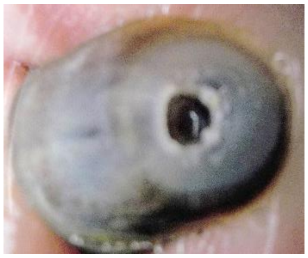

<u>Lampetra Species with Both Features (n=3) – Mix of Features</u>

1. 155 mm, immature adult, Class C tail, medium iris, silver body, slightly sharper teeth (ID=S10, 5/7/2019); Decision Tree Pathway: 1-YES-2A-NO-3-No-4B-No-5-?

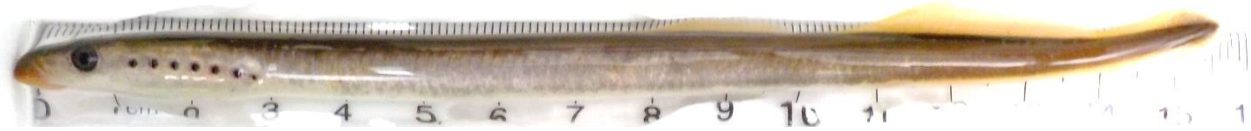

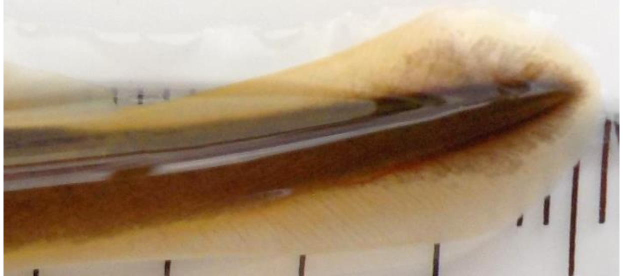
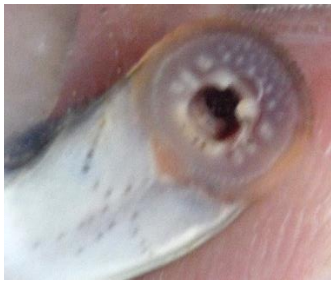

2. 160 mm, juvenile, Class B2 tail, medium iris, dark body, slightly sharper teeth (ID=S16, 5/7/2019); Decision Tree Pathway: 1-YES-2A-NO-3-No-4B-No-5-?

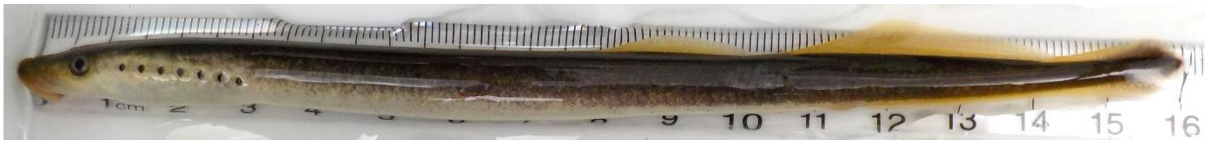

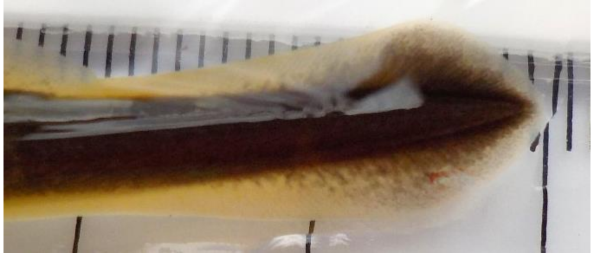
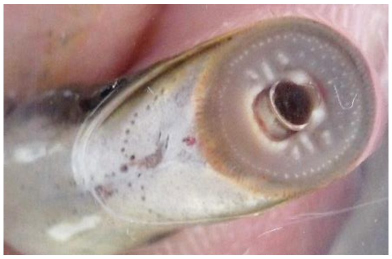

3. 150 mm, immature adult, Class C tail, dark iris, silver body, dull teeth (ID=S21, 5/18/2019); Decision Tree Pathway: 1-YES-2A-NO-3-No-4B-No-5-?

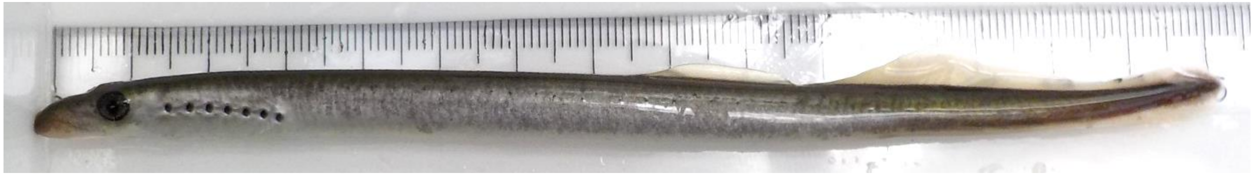

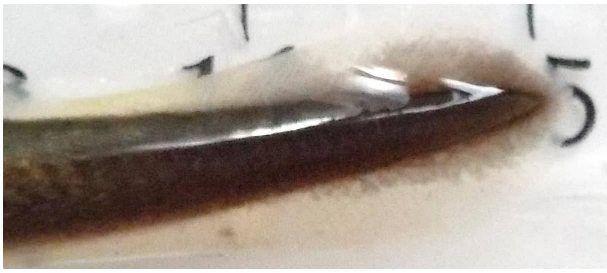
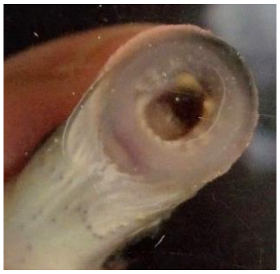

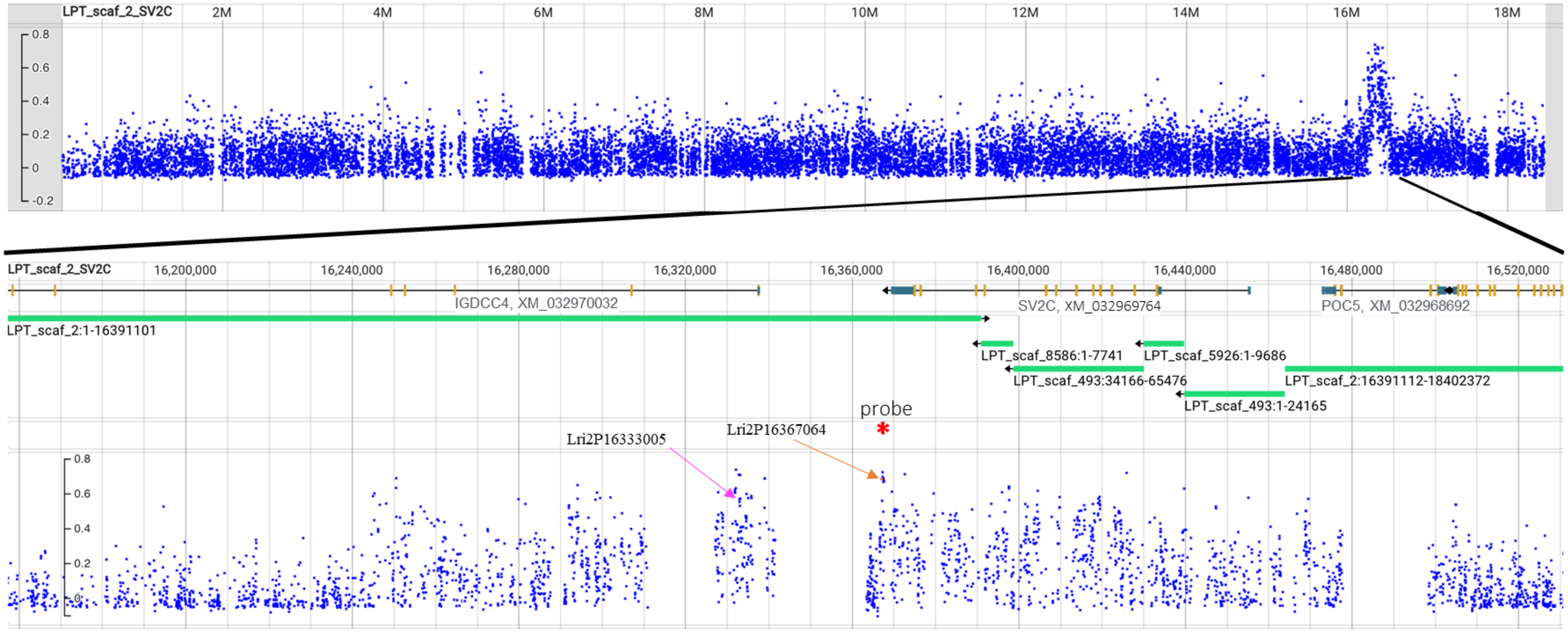

Figure S3. The Manhattan plot of F_{ST} values comparing WBL and WRL specimens from Zolzap Creek aligned to the reassembled *Lampetra richardsoni* scaffold 2 (top) and zoomed in to the region between bp positions 16,160,000 and 16,530,000 in which a peak in F_{ST} values and probes for two candidate SNPs (Lri2P16333005 and Lri2P16367064) are located. Annotation of the high F_{ST} region on *Lampetra richardsoni* scaffold 2 shows high F_{ST} centered on the SV2C lifted over from sea lamprey RefSeq annotation (XM_032969764).

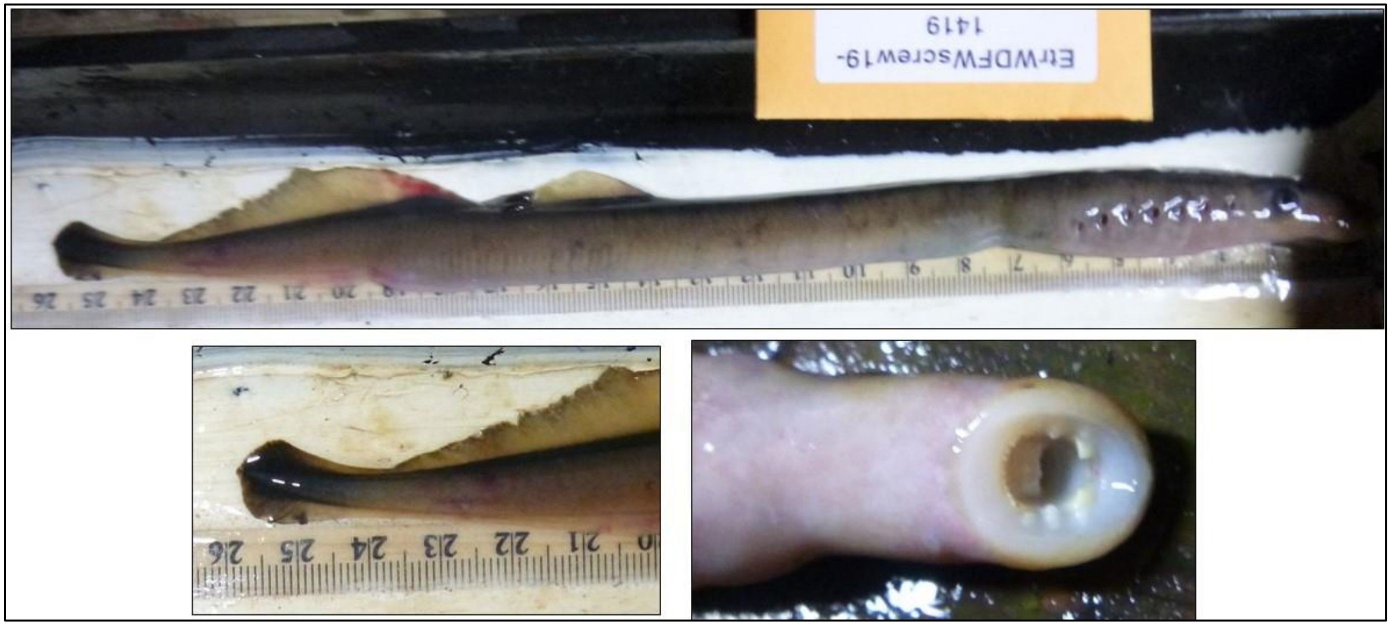

Figure S4. Adult WRL (Western North America genus *Lampetra*) collected at the Cowlitz River screw trap in 2019.

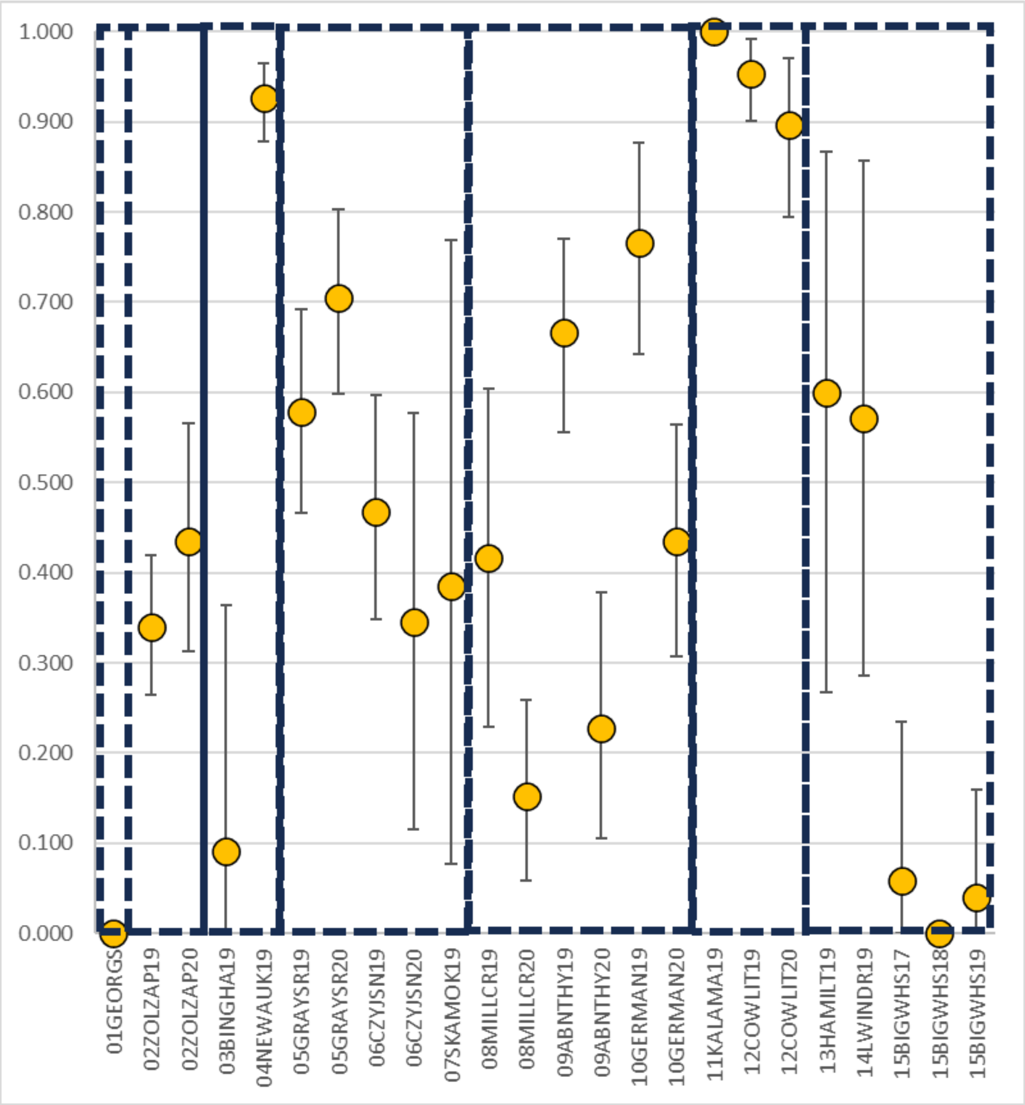

Figure S5. Proportions of *Entosphenus* relative to *Lampetra* across sites and regions (dashed boxes). The 99% confidence intervals are indicated based on 10,000 bootstraps.

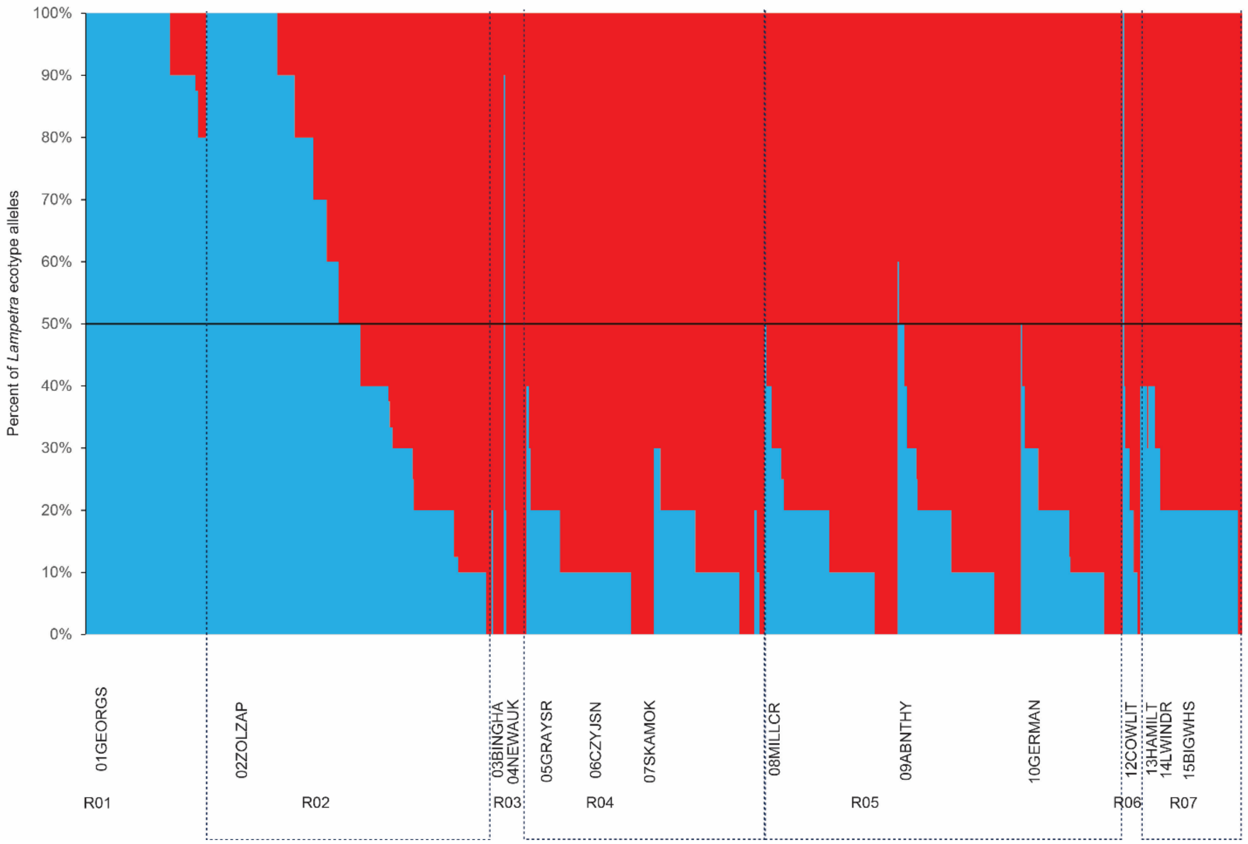

Figure S6. Individual-level composition of WRL alleles used to classify genetic ID of each fish into WRL and WBL ecotypes based on whether the individual had >50% and <50% (indicated by solid black horizontal line) of WRL alleles (blue), respectively, at the five candidate SNPs. Each vertical line in the plot represents an individual (genus *Lampetra*) from across fifteen sites and seven regions (separated by dashed boxes). Red bars indicate the proportion of WBL alleles. Individuals at each site within regions are sorted by greatest to least proportion of WRL alleles.

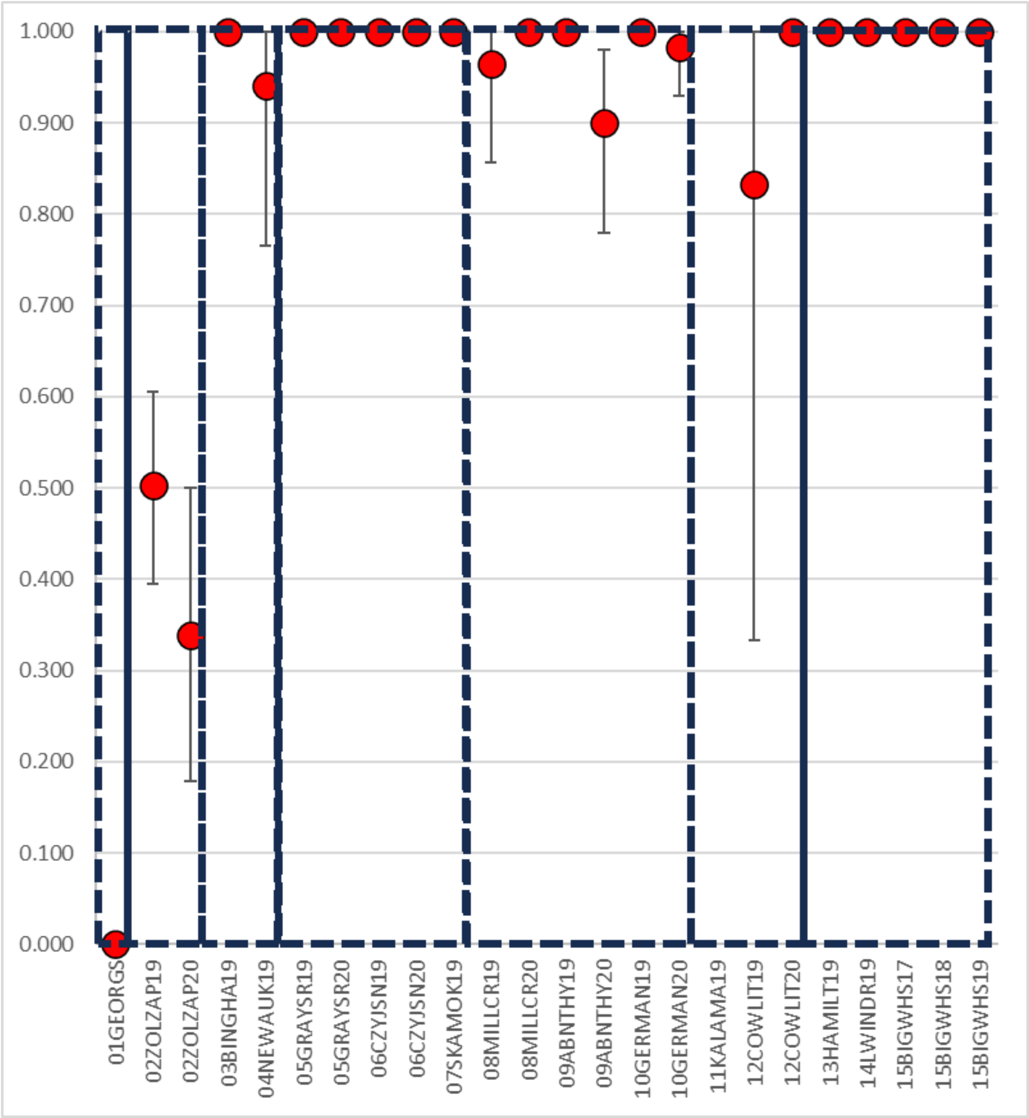

Figure S7. Proportions of WBL ecotypes based on genotypes across sites and regions (dashed boxes).

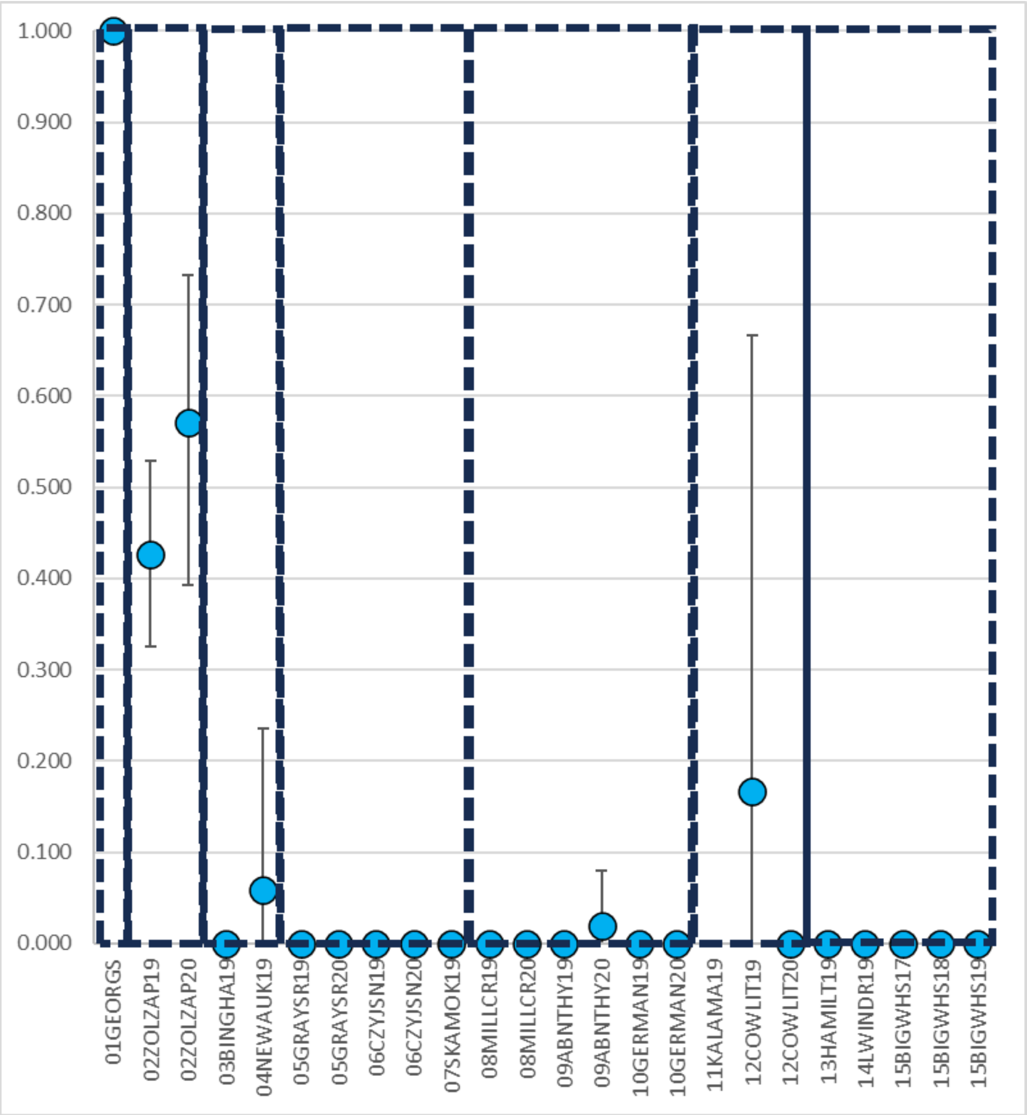

Figure S8. Proportions of the WRL ecotype based on genotypes across sites and regions (dashed boxes).

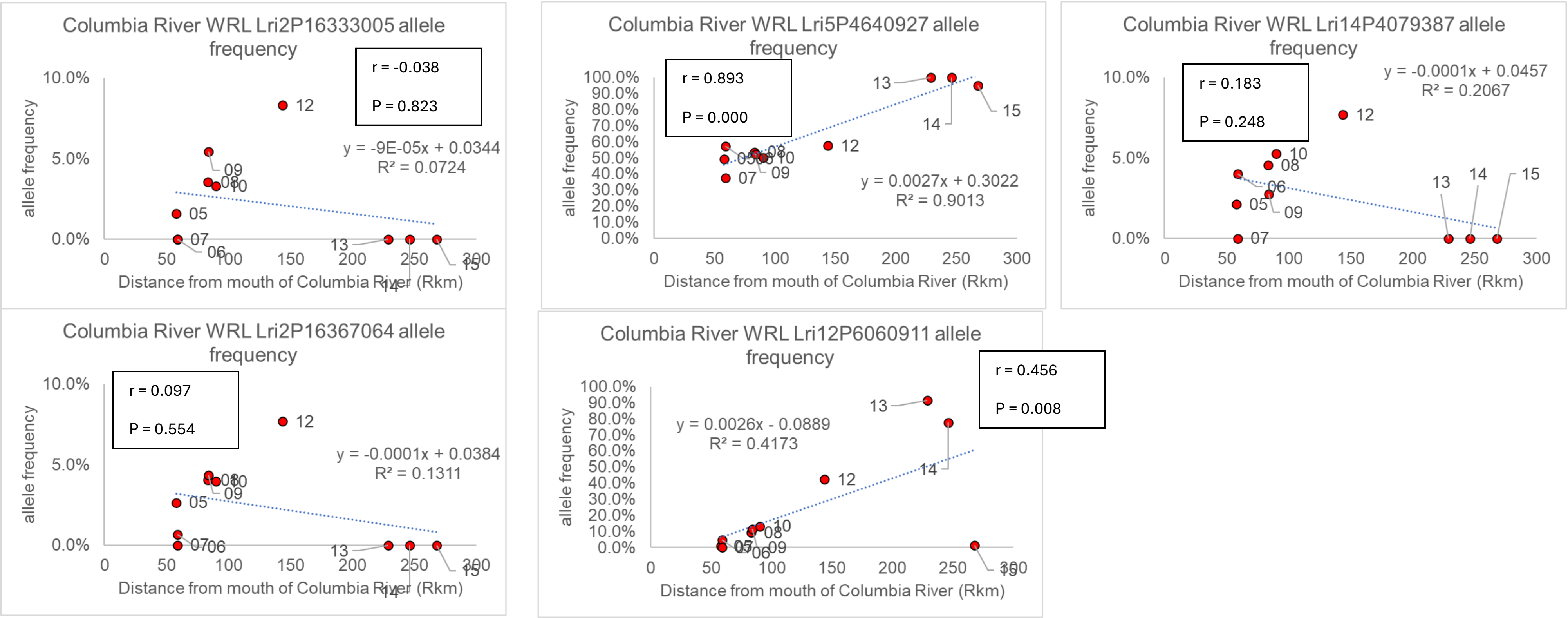

Figure S9. Correlation of *Lampetra* candidate WRL ecotypic allele frequencies with upstream distance (Rkm) in the Columbia River. The five candidate SNP assays that comprised the genetic ID are shown separately. Mantel test correlations "r" and P-values are labeled on each plot.

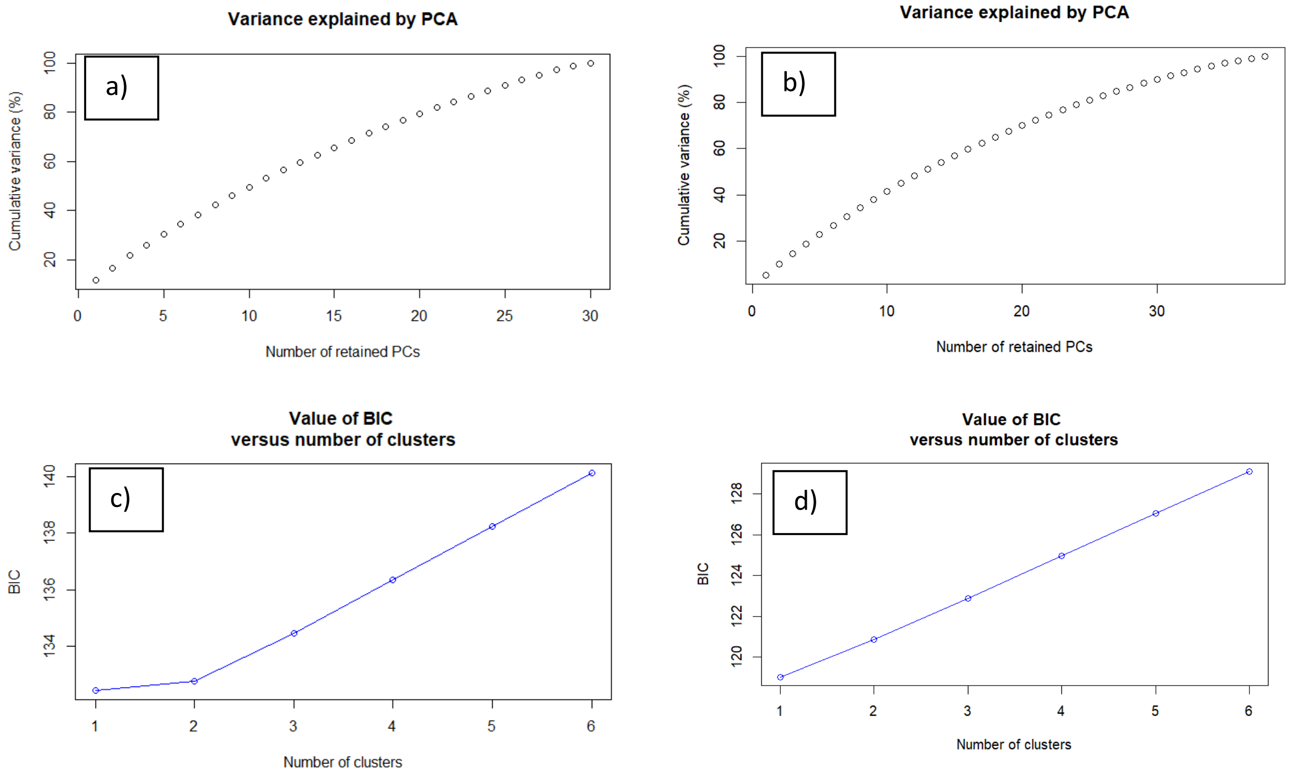

Figure S10. The variance explained by principal component analysis (PCA, top) and the Bayesian Information Criterion (BIC) to compare clustering solutions with increasing number of clusters (bottom) using a dataset of 882 individual SNP genotypes (a, c) and a trimmed subset of 303 SNP genotypes (b, d) with the Ksi Ts'oohl Ts'ap *Lampetra* voucher specimen.

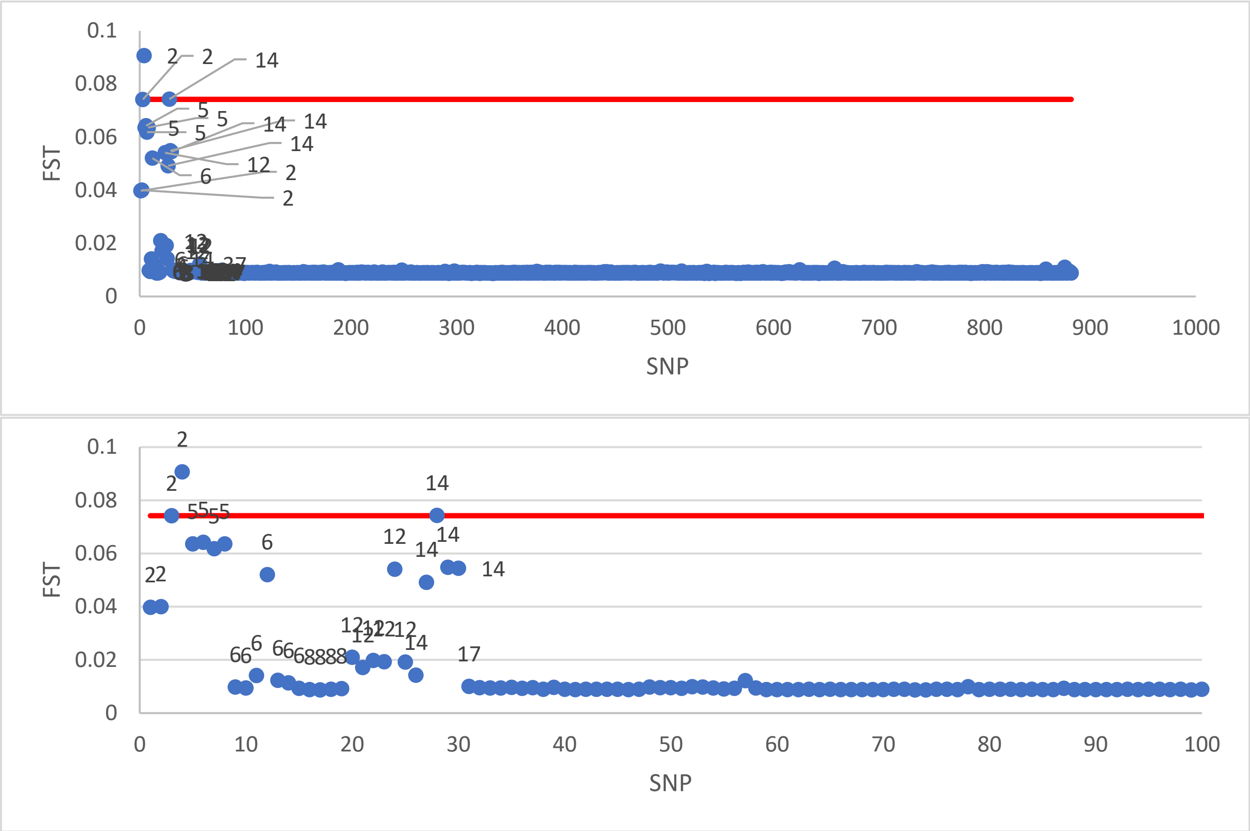

Figure S11. The F_{ST} outlier analysis of individual genotypes of the Ksi Ts'oohl Ts'ap voucher specimen for all 882 SNPs (top) and the first 100 SNPs (bottom) ordered by chromosome position. Labels indicate the chromosome number and the red line indicates the F_{ST} value approximating outliers above a probability of 90%.

Table S1. Distribution of F_{ST} values across 100 scaffolds from Genome Wide Association Study in *Lampetra* ecotypes WBL vs WRL.

		GWAS Markers				N markers with high F_{ST} value			% of chromosome			% across 100 chromosomes			
Scaffold	total_count	count_without_na	min_ F_{ST}	max_ F_{ST}	avg_ F_{ST}	>0.7	>0.6	>0.5	>0.7	>0.6	>0.5	>0.7	>0.6	>0.5	N Assays
1	100655	100655	-0.113	0.769	0.009	1	16	71	0.00%	0.02%	0.07%	0.13%	0.52%	0.75%	
2	92503	92503	-0.112	0.895	0.013	37	83	216	0.04%	0.09%	0.23%	4.91%	2.68%	2.27%	4
3	87003	87003	-0.116	0.786	0.010	4	23	81	0.00%	0.03%	0.09%	0.53%	0.74%	0.85%	
4	78316	78316	-0.125	0.836	0.008	2	11	65	0.00%	0.01%	0.08%	0.27%	0.35%	0.68%	
5	88950	88950	-0.122	0.882	0.037	52	326	1124	0.06%	0.37%	1.26%	6.91%	10.51%	11.80%	4
6	76026	76026	-0.110	0.875	0.029	23	114	361	0.03%	0.15%	0.47%	3.05%	3.68%	3.79%	7
7	70010	70010	-0.112	0.692	0.008	0	5	41	0.00%	0.01%	0.06%	0.00%	0.16%	0.43%	
8	76161	76161	-0.115	0.732	-0.002	1	5	24	0.00%	0.01%	0.03%	0.13%	0.16%	0.25%	2
9	68322	68322	-0.124	0.737	0.006	2	9	39	0.00%	0.01%	0.06%	0.27%	0.29%	0.41%	
10	68788	68788	-0.117	0.748	0.005	1	5	41	0.00%	0.01%	0.06%	0.13%	0.16%	0.43%	
11	55195	55195	-0.104	0.727	0.009	2	11	50	0.00%	0.02%	0.09%	0.27%	0.35%	0.52%	
12	70586	70586	-0.112	1.000	0.112	471	1713	4273	0.67%	2.43%	6.05%	62.55%	55.22%	44.84%	6
13	60251	60251	-0.121	0.667	0.006	0	1	30	0.00%	0.00%	0.05%	0.00%	0.03%	0.31%	
14	47939	47939	-0.125	0.852	0.016	17	81	201	0.04%	0.17%	0.42%	2.26%	2.61%	2.11%	5
15	58414	58414	-0.101	0.667	0.015	0	8	73	0.00%	0.01%	0.12%	0.00%	0.26%	0.77%	
16	54374	54374	-0.111	0.714	0.009	3	4	31	0.01%	0.01%	0.06%	0.40%	0.13%	0.33%	
17	43556	43556	-0.110	0.933	0.104	94	390	1264	0.22%	0.90%	2.90%	12.48%	12.57%	13.26%	7
18	57528	57528	-0.103	0.733	-0.001	2	3	18	0.00%	0.01%	0.03%	0.27%	0.10%	0.19%	2
19	46806	46806	-0.105	0.630	0.010	0	4	32	0.00%	0.01%	0.07%	0.00%	0.13%	0.34%	
20	62803	62803	-0.111	0.706	0.002	2	3	19	0.00%	0.00%	0.03%	0.27%	0.10%	0.20%	
21	62616	62616	-0.111	0.769	0.009	2	5	30	0.00%	0.01%	0.05%	0.27%	0.16%	0.31%	
22	47126	47126	-0.112	0.714	0.008	1	8	28	0.00%	0.02%	0.06%	0.13%	0.26%	0.29%	
23	52509	52509	-0.106	0.727	0.011	3	10	49	0.01%	0.02%	0.09%	0.40%	0.32%	0.51%	
24	58910	58910	-0.111	0.784	0.042	6	53	233	0.01%	0.09%	0.40%	0.80%	1.71%	2.45%	6
25	51562	51562	-0.115	0.652	0.005	0	5	34	0.00%	0.01%	0.07%	0.00%	0.16%	0.36%	
26	35147	35147	-0.102	0.620	0.004	0	2	20	0.00%	0.01%	0.06%	0.00%	0.06%	0.21%	

27	40313	40313	-0.132	0.631	0.011	0	3	27	0.00%	0.01%	0.07%	0.00%	0.10%	0.28%	
28	37114	37114	-0.117	0.687	0.011	0	11	34	0.00%	0.03%	0.09%	0.00%	0.35%	0.36%	
29	27231	27231	-0.127	0.752	0.010	2	9	27	0.01%	0.03%	0.10%	0.27%	0.29%	0.28%	
30	28842	28842	-0.101	0.636	0.004	0	1	14	0.00%	0.00%	0.05%	0.00%	0.03%	0.15%	
31	27769	27769	-0.103	0.661	0.036	0	8	79	0.00%	0.03%	0.28%	0.00%	0.26%	0.83%	0
32	43128	43128	-0.105	0.756	0.006	1	6	20	0.00%	0.01%	0.05%	0.13%	0.19%	0.21%	
33	23172	23172	-0.112	0.740	0.012	1	2	24	0.00%	0.01%	0.10%	0.13%	0.06%	0.25%	
34	25166	25166	-0.110	0.651	0.010	0	3	29	0.00%	0.01%	0.12%	0.00%	0.10%	0.30%	
35	31031	31031	-0.111	0.727	0.012	2	11	26	0.01%	0.04%	0.08%	0.27%	0.35%	0.27%	
36	23436	23436	-0.097	0.667	0.010	0	2	19	0.00%	0.01%	0.08%	0.00%	0.06%	0.20%	
37	39030	39030	-0.109	0.733	0.020	1	17	89	0.00%	0.04%	0.23%	0.13%	0.55%	0.93%	4
38	16254	16254	-0.103	0.580	0.009	0	0	11	0.00%	0.00%	0.07%	0.00%	0.00%	0.12%	
39	20908	20908	-0.101	0.690	0.013	0	7	36	0.00%	0.03%	0.17%	0.00%	0.23%	0.38%	
40	25033	25033	-0.121	0.694	0.010	0	2	22	0.00%	0.01%	0.09%	0.00%	0.06%	0.23%	
41	14481	14481	-0.105	0.692	0.012	0	3	17	0.00%	0.02%	0.12%	0.00%	0.10%	0.18%	
42	20495	20495	-0.104	0.786	0.006	3	5	17	0.01%	0.02%	0.08%	0.40%	0.16%	0.18%	
43	31136	31136	-0.100	0.674	0.005	0	2	12	0.00%	0.01%	0.04%	0.00%	0.06%	0.13%	
44	17603	17603	-0.102	0.636	0.010	0	1	6	0.00%	0.01%	0.03%	0.00%	0.03%	0.06%	
45	26334	26334	-0.133	0.740	0.007	2	4	19	0.01%	0.02%	0.07%	0.27%	0.13%	0.20%	
46	26664	26664	-0.101	0.643	0.005	0	2	18	0.00%	0.01%	0.07%	0.00%	0.06%	0.19%	
47	25090	25090	-0.111	0.655	0.010	0	5	21	0.00%	0.02%	0.08%	0.00%	0.16%	0.22%	
48	20631	20631	-0.111	0.767	0.008	1	1	16	0.00%	0.00%	0.08%	0.13%	0.03%	0.17%	
49	32583	32583	-0.101	0.675	0.004	0	2	25	0.00%	0.01%	0.08%	0.00%	0.06%	0.26%	
50	14749	14749	-0.100	0.646	0.011	0	4	22	0.00%	0.03%	0.15%	0.00%	0.13%	0.23%	
51	15132	15132	-0.100	0.818	0.009	2	2	17	0.01%	0.01%	0.11%	0.27%	0.06%	0.18%	
52	22995	22995	-0.105	0.667	0.014	0	7	24	0.00%	0.03%	0.10%	0.00%	0.23%	0.25%	
53	23232	23232	-0.117	0.647	0.012	0	5	21	0.00%	0.02%	0.09%	0.00%	0.16%	0.22%	
54	13734	13734	-0.108	0.688	0.020	0	2	9	0.00%	0.01%	0.07%	0.00%	0.06%	0.09%	Soussi
55	12773	12773	-0.110	0.729	0.011	1	2	8	0.01%	0.02%	0.06%	0.13%	0.06%	0.08%	
56	23790	23790	-0.111	0.733	0.007	2	3	13	0.01%	0.01%	0.05%	0.27%	0.10%	0.14%	
57	15099	15099	-0.111	0.643	0.010	0	4	17	0.00%	0.03%	0.11%	0.00%	0.13%	0.18%	
58	18905	18905	-0.102	0.688	0.003	0	4	14	0.00%	0.02%	0.07%	0.00%	0.13%	0.15%	

59	26512	26512	-0.106	0.529	0.001	0	0	4	0.00%	0.00%	0.02%	0.00%	0.00%	0.04%
60	19564	19564	-0.110	0.613	0.009	0	3	19	0.00%	0.02%	0.10%	0.00%	0.10%	0.20%
61	19575	19575	-0.095	0.667	0.008	0	3	13	0.00%	0.02%	0.07%	0.00%	0.10%	0.14%
62	12520	12520	-0.108	0.610	0.011	0	1	9	0.00%	0.01%	0.07%	0.00%	0.03%	0.09%
63	19961	19961	-0.122	0.733	0.009	1	8	21	0.01%	0.04%	0.11%	0.13%	0.26%	0.22%
64	11647	11647	-0.101	0.647	0.012	0	1	8	0.00%	0.01%	0.07%	0.00%	0.03%	0.08%
65	18301	18301	-0.111	0.539	0.006	0	0	5	0.00%	0.00%	0.03%	0.00%	0.00%	0.05%
66	19782	19782	-0.100	0.652	0.012	0	1	17	0.00%	0.01%	0.09%	0.00%	0.03%	0.18%
67	10276	10276	-0.094	0.539	0.009	0	0	10	0.00%	0.00%	0.10%	0.00%	0.00%	0.10%
68	18276	18276	-0.100	0.545	0.007	0	0	6	0.00%	0.00%	0.03%	0.00%	0.00%	0.06%
69	25025	25025	-0.103	0.615	-0.002	0	1	5	0.00%	0.00%	0.02%	0.00%	0.03%	0.05%
70	12492	12492	-0.111	0.691	0.010	0	2	8	0.00%	0.02%	0.06%	0.00%	0.06%	0.08%
71	7919	7919	-0.100	0.571	0.006	0	0	4	0.00%	0.00%	0.05%	0.00%	0.00%	0.04%
72	12725	12725	-0.109	0.769	0.009	1	5	10	0.01%	0.04%	0.08%	0.13%	0.16%	0.10%
73	8795	8795	-0.095	0.641	0.011	0	1	8	0.00%	0.01%	0.09%	0.00%	0.03%	0.08%
74	15917	15917	-0.095	0.692	0.003	0	1	6	0.00%	0.01%	0.04%	0.00%	0.03%	0.06%
75	9605	9605	-0.100	0.611	0.007	0	1	3	0.00%	0.01%	0.03%	0.00%	0.03%	0.03%
76	18245	18245	-0.103	0.643	0.007	0	3	12	0.00%	0.02%	0.07%	0.00%	0.10%	0.13%
77	10121	10121	-0.111	0.565	0.009	0	0	5	0.00%	0.00%	0.05%	0.00%	0.00%	0.05%
78	12501	12501	-0.111	0.594	0.005	0	0	5	0.00%	0.00%	0.04%	0.00%	0.00%	0.05%
79	10958	10958	-0.100	0.545	0.036	0	0	4	0.00%	0.00%	0.04%	0.00%	0.00%	0.04%
80	16819	16819	-0.111	0.693	0.002	0	1	5	0.00%	0.01%	0.03%	0.00%	0.03%	0.05%
81	33182	33182	-0.107	0.630	0.001	0	2	6	0.00%	0.01%	0.02%	0.00%	0.06%	0.06%
82	53617	53617	-0.130	0.706	0.000	2	6	18	0.00%	0.01%	0.03%	0.27%	0.19%	0.19%
83	12501	12501	-0.099	0.625	0.011	0	1	14	0.00%	0.01%	0.11%	0.00%	0.03%	0.15%
84	8358	8358	-0.095	0.539	0.010	0	0	4	0.00%	0.00%	0.05%	0.00%	0.00%	0.04%
85	8625	8625	-0.100	0.647	0.012	0	3	6	0.00%	0.03%	0.07%	0.00%	0.10%	0.06%
86	9442	9442	-0.111	0.786	0.029	4	5	7	0.04%	0.05%	0.07%	0.53%	0.16%	0.07%
87	8342	8342	-0.100	0.593	0.001	0	0	6	0.00%	0.00%	0.07%	0.00%	0.00%	0.06%
88	26995	26995	-0.097	0.600	-0.004	0	1	1	0.00%	0.00%	0.00%	0.00%	0.03%	0.01%
89	11465	11465	-0.101	0.533	0.006	0	0	1	0.00%	0.00%	0.01%	0.00%	0.00%	0.01%
90	23202	23202	-0.103	0.545	-0.002	0	0	4	0.00%	0.00%	0.02%	0.00%	0.00%	0.04%

91	7170	7170	-0.095	0.623	0.012	0	1	4	0.00%	0.01%	0.06%	0.00%	0.03%	0.04%	
92	25882	25882	-0.101	0.695	-0.004	0	1	2	0.00%	0.00%	0.01%	0.00%	0.03%	0.02%	
93	10021	10021	-0.103	0.635	0.012	0	3	9	0.00%	0.03%	0.09%	0.00%	0.10%	0.09%	
94	24557	24557	-0.108	0.545	-0.006	0	0	4	0.00%	0.00%	0.02%	0.00%	0.00%	0.04%	
95	24480	24480	-0.113	0.648	0.011	0	6	29	0.00%	0.02%	0.12%	0.00%	0.19%	0.30%	
96	4661	4661	-0.092	0.521	0.005	0	0	1	0.00%	0.00%	0.02%	0.00%	0.00%	0.01%	
97	13673	13673	-0.125	0.603	0.010	0	1	11	0.00%	0.01%	0.08%	0.00%	0.03%	0.12%	
98	19624	19624	-0.107	0.769	0.008	1	2	8	0.01%	0.01%	0.04%	0.13%	0.06%	0.08%	
99	17294	17294	-0.111	0.462	-0.003	0	0	0	0.00%	0.00%	0.00%	0.00%	0.00%	0.00%	
100	15357	15357	-0.127	0.563	0.000	0	0	6	0.00%	0.00%	0.04%	0.00%	0.00%	0.06%	
Total	3151893	3151893				753	3102	9529							47

Table S2. The candidate genomic regions for distinguishing *Lampetra* ecotypes WBL and WRL from Zolzap Creek.

Interval	# SNPs	Avg FST	Min FST	Max FST	Peak	Scaffold	Beginning	End	Length (bp)
Peak Chr2 16245186…16395922	32	0.763	0.700	0.895	Peak01	2	16245186	16395922	150736
Peak Chr5 3628582…5206447	48	0.751	0.703	0.882	Peak01	5	3628582	5206447	1577865
Peak01 Chr6 5962341…6693459	20	0.681	0.611	0.813	Peak01	6	5962341	6693459	731118
Peak02 Chr6 7916583…8928386	13	0.684	0.603	0.875	Peak02	6	7916583	8928386	1011803
Peak03 Chr6 9708392…10599905	63	0.666	0.606	0.863	Peak03	6	9708392	10599905	891513
Peak Chr8 3316198…4295266	14	0.586	0.513	0.732	Peak01	8	3316198	4295266	979068
Peak Chr12 8539…7731245	449	0.761	0.701	1.000	Peak01	12	8539	7731245	7722706
Peak Chr14 3895562…4107888	75	0.663	0.601	0.852	Peak01	14	3895562	4107888	212326
Peak Chr17 4533…9760331	92	0.745	0.701	0.933	Peak01	17	4533	9760331	9755798
Peak Chr18 4745725…9162364	4	0.608	0.513	0.707	Peak01	18	4745725	9162364	4416639
Peak Chr24 19369…8845755	158	0.580	0.506	0.784	Peak01	24	19369	8845755	8826386
Peak1 Chr37 1037618…2398163	25	0.543	0.500	0.629	Peak01	37	1037618	2398163	1360545
Peak2 Chr37 3156929…4293120	60	0.545	0.500	0.733	Peak02	37	3156929	4293120	1136191

Table S3. Basic genotypic results from the morphological ID specimens of WBL and WRL that were used to identify candidate SNPs for species identification.

		Genotype		WBL				WRL				Abs Diff		*F*ST	
**Locus #**	**Locus Name**	**Hom01**	**Hom02**	**Hom01**	**Het**	**Hom02**	**fail**	**Hom01**	**Het**	**Hom02**	**fail**	**Hom01**	**Hom02**	**Pool-seq**	**GT-seq**
LOC01	Lri2P16247548	AA	TT	4.8%	28.6%	66.7%	12.5%	54.5%	36.4%	9.1%	26.7%	49.8%	57.6%	0.784	0.439
LOC02	Lri2P16250399	CC	TT	85.7%	14.3%	0.0%	12.5%	27.3%	36.4%	36.4%	26.7%	58.4%	36.4%	0.724	0.444
LOC03	Lri2P16333005	AA	TT	13.3%	13.3%	73.3%	37.5%	87.5%	0.0%	12.5%	46.7%	74.2%	60.8%	0.870	0.583
LOC04	Lri2P16367064	AA	GG	76.2%	14.3%	9.5%	12.5%	9.1%	18.2%	72.7%	26.7%	67.1%	63.2%	0.877	0.578
LOC05	Lri5P3706885	AA	GG	28.6%	57.1%	14.3%	12.5%	0.0%	9.1%	90.9%	26.7%	28.6%	76.6%	0.750	0.424
LOC06	Lri5P4269100	AA	GG	14.3%	57.1%	28.6%	12.5%	90.9%	9.1%	0.0%	26.7%	76.6%	28.6%	0.706	0.424
LOC07	Lri5P4640927	AA	TT	11.1%	55.6%	33.3%	25.0%	90.0%	10.0%	0.0%	33.3%	78.9%	33.3%	0.765	0.464
LOC08	Lri5P5071040	AA	TT	28.6%	57.1%	14.3%	12.5%	0.0%	9.1%	90.9%	26.7%	28.6%	76.6%	0.721	0.424
LOC09	Lri6P10286942	AA	TT	19.0%	61.9%	19.0%	12.5%	0.0%	18.2%	81.8%	26.7%	19.0%	62.8%	0.684	0.283
LOC10	Lri6P10328882	AA	TT	28.6%	52.4%	19.0%	12.5%	0.0%	9.1%	90.9%	26.7%	28.6%	71.9%	0.677	0.396
LOC11	Lri6P10383294	CC	GG	19.0%	47.6%	33.3%	12.5%	72.7%	27.3%	0.0%	26.7%	53.7%	33.3%	0.606	0.296
LOC12	Lri6P10474030	CC	TT	20.0%	45.0%	35.0%	16.7%	63.6%	36.4%	0.0%	26.7%	43.6%	35.0%	0.641	0.242
LOC13	Lri6P10581411	CC	TT	14.3%	66.7%	19.0%	12.5%	9.1%	27.3%	63.6%	26.7%	5.2%	44.6%	0.778	0.095
LOC14	Lri6P6080845	AA	GG	38.1%	52.4%	9.5%	12.5%	18.2%	27.3%	54.5%	26.7%	19.9%	45.0%	0.784	0.159
LOC15	Lri6P8338530	CC	TT	47.6%	42.9%	9.5%	12.5%	36.4%	18.2%	45.5%	26.7%	11.3%	35.9%	0.603	0.069
LOC16	Lri8P4138772	AA	TT	23.8%	42.9%	33.3%	12.5%	9.1%	36.4%	54.5%	26.7%	14.7%	21.2%	0.526	0.027
LOC17	Lri8P4295266	GG	TT	42.9%	57.1%	0.0%	12.5%	18.2%	54.5%	27.3%	26.7%	24.7%	27.3%	0.732	0.111
LOC18	Lri12P182981	AA	CC	4.8%	38.1%	57.1%	12.5%	60.0%	30.0%	10.0%	33.3%	55.2%	47.1%	0.729	0.394
LOC19	Lri12P183087	AA	GG	5.3%	36.8%	57.9%	20.8%	54.5%	36.4%	9.1%	26.7%	49.3%	48.8%	0.709	0.368
LOC20	Lri12P279478	AA	GG	57.1%	38.1%	4.8%	12.5%	9.1%	36.4%	54.5%	26.7%	48.1%	49.8%	0.889	0.368
LOC21	Lri12P5470970	AA	GG	4.8%	38.1%	57.1%	12.5%	54.5%	36.4%	9.1%	26.7%	49.8%	48.1%	0.750	0.368
LOC22	Lri12P6060911	CC	TT	66.7%	33.3%	0.0%	12.5%	9.1%	36.4%	54.5%	26.7%	57.6%	54.5%	0.765	0.480
LOC23	Lri12P7199624	AA	GG	57.1%	38.1%	4.8%	12.5%	9.1%	36.4%	54.5%	26.7%	48.1%	49.8%	0.784	0.368
LOC24	Lri14P3895562	GG	TT	57.1%	14.3%	28.6%	12.5%	9.1%	27.3%	63.6%	26.7%	48.1%	35.1%	0.615	0.251
LOC25	Lri14P3943878	GG	TT	14.3%	23.8%	61.9%	12.5%	63.6%	27.3%	9.1%	26.7%	49.4%	52.8%	0.607	0.383
LOC26	Lri14P4079387	AA	CC	9.5%	23.8%	66.7%	12.5%	63.6%	27.3%	9.1%	26.7%	54.1%	57.6%	0.616	0.454

LOC27	Lri14P4102727	AA	GG	9.5%	23.8%	66.7%	12.5%	63.6%	18.2%	18.2%	26.7%	54.1%	48.5%	0.772	0.396
LOC28	Lri14P4106054	AA	GG	9.5%	23.8%	66.7%	12.5%	63.6%	18.2%	18.2%	26.7%	54.1%	48.5%	0.603	0.396
LOC29	Lri17P2957024	AA	GG	33.3%	38.1%	28.6%	12.5%	9.1%	18.2%	72.7%	26.7%	24.2%	44.2%	0.722	0.178
LOC30	Lri17P5047059	GG	TT	28.6%	28.6%	42.9%	12.5%	54.5%	36.4%	9.1%	26.7%	26.0%	33.8%	0.729	0.123
LOC31	Lri17P6620172	CC	TT	19.0%	42.9%	38.1%	12.5%	63.6%	18.2%	18.2%	26.7%	44.6%	19.9%	0.933	0.150
LOC32	Lri17P7594234TD43	AA	CC	33.3%	33.3%	33.3%	12.5%	9.1%	27.3%	63.6%	26.7%	24.2%	30.3%	0.769	0.101
LOC33	Lri17P7943131	AA	CC	33.3%	33.3%	33.3%	12.5%	9.1%	27.3%	63.6%	26.7%	24.2%	30.3%	0.787	0.101
LOC34	Lri17P8999134	GG	TT	33.3%	33.3%	33.3%	12.5%	9.1%	27.3%	63.6%	26.7%	24.2%	30.3%	0.722	0.101
LOC35	Lri17P9471416	AA	GG	35.0%	30.0%	35.0%	16.7%	9.1%	27.3%	63.6%	26.7%	25.9%	28.6%	0.714	0.099
LOC36	Lri18P4745725	AA	GG	23.8%	57.1%	19.0%	12.5%	45.5%	45.5%	9.1%	26.7%	21.6%	10.0%	0.619	0.019
LOC37	Lri18P6463787	AA	CC	9.5%	23.8%	66.7%	12.5%	18.2%	36.4%	45.5%	26.7%	8.7%	21.2%	0.707	0.012
LOC38	Lri24P3183281	AA	CC	19.0%	42.9%	38.1%	12.5%	0.0%	27.3%	72.7%	26.7%	19.0%	34.6%	0.583	0.121
LOC39	Lri24P3549440	AA	CC	19.0%	52.4%	28.6%	12.5%	0.0%	36.4%	63.6%	26.7%	19.0%	35.1%	0.671	0.118
LOC40	Lri24P4354883	AA	TT	38.1%	47.6%	14.3%	12.5%	72.7%	27.3%	0.0%	26.7%	34.6%	14.3%	0.571	0.102
LOC41	Lri24P6808546	AA	TT	38.1%	47.6%	14.3%	12.5%	72.7%	27.3%	0.0%	26.7%	34.6%	14.3%	0.583	0.102
LOC42	Lri24P7481704	AA	GG	14.3%	47.6%	38.1%	12.5%	0.0%	20.0%	80.0%	33.3%	14.3%	41.9%	0.600	0.142
LOC43	Lri24P7765916	GG	TT	33.3%	52.4%	14.3%	12.5%	72.7%	27.3%	0.0%	26.7%	39.4%	14.3%	0.542	0.126
LOC44	Lri37P1167580	GG	TT	14.3%	52.4%	33.3%	12.5%	0.0%	30.0%	70.0%	33.3%	14.3%	36.7%	0.629	0.107
LOC45	Lri37P1334668	AA	TT	19.0%	9.5%	71.4%	12.5%	0.0%	18.2%	81.8%	26.7%	19.0%	10.4%	0.513	0.014
LOC46	Lri37P1937714	CC	TT	26.3%	21.1%	52.6%	20.8%	0.0%	22.2%	77.8%	40.0%	26.3%	25.1%	0.506	0.095
LOC47	Lri37P4230445	CC	TT	33.3%	42.9%	23.8%	12.5%	90.9%	0.0%	9.1%	26.7%	57.6%	14.7%	0.529	0.221

Note: The top candidate SNPs that provided highest F_{ST} after genotyping with the GT-seq assay are highlighted in gray and were used to genotype and screen all individuals included in the study. F_{ST} values were calculated using either “Pool-seq” or “GT-seq” data for the 39 specimens that represented WBL and WRL from Zolzap Creek, B.C. based on morphological ID. Genotypes were labeled as homozygote “Hom01” or “Hom02” for alternate homozygous alleles, and “Het” for heterzogous state. Allele frequencies for the 24 WBL and 15 WRL specimens are indicated as well as the number from each ecotype category that failed to genotype (“fail”). The absolute difference “Abs Diff” in genotypic frequencies between ecotypes is shown.

Table S4. Genome alignments of islands of divergence in *Lampetra* ecotypes and sea lamprey reference genome.

										Lampetra richardsoni			sea lamprey			
Scaffold	Peak	Beginning	End	Length (bp)	# SNPs	Avg FST	Min FST	Max FST	Final	Scaffold	hit start	hit end	chromosome	hit start	hit end	genes
2	Peak 01	16245186	16395922	150736	32	0.763	0.700	0.895	4	LPT_scaf_2	16245607	16399816	chr1	16638734	16415030	SV2C; chr1:16522168..16523742 (+ strand); chr1:16464930..16479212 (+ strand)
5	Peak 01	3628582	5206447	1577865	48	0.751	0.703	0.882	4	LPT_scaf_5	3629622	5214219	chr6	14060239	12447306	NIN; chr6:13023472..13054298 (+ strand)
6	Peak 01	5962341	6693459	731118	20	0.681	0.611	0.813	1	LPT_scaf_6	5960968	6715510	chr10	6779016	7593635	
6	Peak 02	7916583	8928386	1011803	13	0.684	0.603	0.875	1	LPT_scaf_6	7910923	8930175	chr10	8727950	9845373	
6	Peak 03	9708392	10599905	891513	63	0.666	0.606	0.863	5	LPT_scaf_6	9702082	10634377	chr10	10640023	11640929	
8	Peak 01	3316198	4295266	979068	14	0.586	0.513	0.732	2	LPT_scaf_8	3307507	4296747	chr2	4991845	6021236	
12	Peak 01	8539	7731245	7722706	449	0.761	0.701	1.000	6	LPT_scaf_12	7021	7776247	chr34	12479906	4159269	ACVR2B; chr34:6001249..6015147 (-strand)
14	Peak 01	3895562	4107888	212326	75	0.663	0.601	0.852	5	LPT_scaf_14	3885488	4118977	chr31	4241500	4475575	ESR1; chr31:4401050..4460418 (+ strand)
17	Peak 01	4533	9760331	9755798	92	0.745	0.701	0.933	7	LPT_scaf_17	0	9164314	chr25	13528699	3580105	
18	Peak 01	4745725	9162364	4416639	4	0.608	0.513	0.707	2	LPT_scaf_18	4742176	9141728	chr2	7716644	12085544	
24	Peak 01	19369	8845755	8826386	158	0.580	0.506	0.784	6	LPT_scaf_24	21683	8666905	chr8	57519	9198203	
31	Peak 01	5324511	6768092	1443581	66	0.542	0.500	0.661	0	LPT_scaf_31	5323150	6779633	chr8	11013101	9447916	
37	Peak 01	1037618	2398163	1360545	25	0.543	0.500	0.629	3	LPT_scaf_37	1028574	2402626	chr15	9982132	8546489	
37	Peak 02	3156929	4293120	1136191	60	0.545	0.500	0.733	1	LPT_scaf_37	3150398	4295857	chr15	7787476	6536918	

Genes indicates the annotated regions in sea lamprey that were closest to the candidate SNPs identified on multiple chromosomes in the *Lampetra richardsoni* genome and developed into a 5-SNP candidate assay for *Lampetra* ecotype ID.

Table S5. Genome alignments of candidate loci from Soussi et al. (2022) for European *Lampetra* ecotypes and sea lamprey reference genome

Table S2: Summary of the genomic location of the 5 selected SNP markers in the *L. planeri* and *P. marinus* genomes.

				Minimap results				sea lamprey			Minimap results				Lampetra richardsoni	
SNP	Fst	Location in *L. planeri* genome	Location in *P. marinus* genome	old_sea_lamprey	hit start	hit end	chromosome	hit start	hit end	genes	old_sea_lamprey	hit start	hit end	LPT_scaf_54	hit start	hit end
diagLpf	1	Ctg.1179017	GL476391 :496268-496341	GL476391.1	473440	506621	chr64	3377412	3410955		GL476391.1	495584	498897	LPT_scaf_54	3373668	3377352
SNP 09	1	Ctg.2478209	GL488821 : 41-6585	GL488821.1	0	6637	chr64	3990725	3997418		GL488821.1	5	6533	LPT_scaf_54	2864454	2870967
SNP 22	0.9936	Ctg.3770922	GL479158 :18018 - 18635	GL479158.1	174222	182176	chr64	4625502	4633049		GL479158.1	2221	25952	LPT_scaf_54	2187106	2211453
SNP 83	0.9936	Ctg.4537583	GL482155 :2780 - 4033	GL482155.1	34	13500	chr64	5249247	5262594		GL482155.1	1961	4033	LPT_scaf_54	1831615	1833852
SNP 04	0.9936	Ctg.4559404	GL476391 :366204-240647	GL476391.1	260344	326471	chr64	3171553	3238163		GL476391.1	257754	259768	LPT_scaf_54	3575168	3577307
				GL476391.1	327473	328801	chr64	3239361	3240689			260351	266636	LPT_scaf_54	3568820	3574563
				GL476391.1	353954	358443	chr64	3256584	3261165			266827	287938	LPT_scaf_54	3546339	3568456
				GL476391.1	359447	429232	chr64	3262437	3333588			290311	303968	LPT_scaf_54	3532494	3545550
												304629	313973	LPT_scaf_54	3520705	3529230
												315230	326347	LPT_scaf_54	3511361	3520377
												327473	328789	LPT_scaf_54	3509194	3510401
												355289	358443	LPT_scaf_54	3491442	3494544
												364100	367977	LPT_scaf_54	3482884	3486225

Table S6. Ecotype ID concordance based on morphology and 5 candidate SNPs.

		5-SNP Assay		Morph. vs Gen. ID		Morph. vs Gen. ID		Morph. vs Gen. ID		Morph. vs Gen. ID		Morph. vs Gen. ID		Morph. vs Gen. ID
ID	MorphID	%WRL	Ecotype	Concordance	Lri2P16333005	Concordance	Lri2P16367064	Concordance	Lri5P4640927	Concordance	Lri12P6060911	Concordance	Lri14P4079387	Concordance
LayZolzap19Adult-S0001	WBL	25.00%	WBL	Match	-	-	WBL	Match	WBL	Match	WBL	Match	WRL	FALSE
LayZolzap19Adult-S0010	WBL	20.00%	WBL	Match	WBL	Match	WBL	Match	Intermediate	0	Intermediate	0	Intermediate	0
LayZolzap19Adult-S0011	WBL	10.00%	WBL	Match	WBL	Match	WBL	Match	Intermediate	0	Intermediate	0	WBL	Match
LayZolzap19Adult-S0012	WBL	33.30%	WBL	Match	-	-	WBL	Match	-	-	-	-	Intermediate	0
LayZolzap19Adult-S0014	WBL	12.50%	WBL	Match	WBL	Match	WBL	Match	-	-	-	-	WBL	Match
LayZolzap19Adult-S0015	WBL	12.50%	WBL	Match	-	-	WBL	Match	Intermediate	0	Intermediate	0	WBL	Match
LayZolzap19Adult-S0016	WBL	50.00%	Intermediate	0	WRL	FALSE	WRL	FALSE	WBL	Match	WBL	Match	Intermediate	0
LayZolzap19Adult-S0017	WBL	30.00%	WBL	Match	Intermediate	0	Intermediate	0	WBL	Match	WBL	Match	WBL	Match
LayZolzap19Adult-S0018	WBL	30.00%	WBL	Match	WBL	Match	WBL	Match	Intermediate	0	Intermediate	0	Intermediate	0
LayZolzap19Adult-S0021	WBL	70.00%	WRL	FALSE	WRL	FALSE	WRL	FALSE	Intermediate	0	Intermediate	0	Intermediate	0
LriZolzap19Adult-B0001	WBL	20.00%	WBL	Match	Intermediate	0	Intermediate	0	WBL	Match	WBL	Match	WBL	Match
LriZolzap19Adult-B0002	WBL	12.50%	WBL	Match	-	-	WBL	Match	Intermediate	0	Intermediate	0	WBL	Match
LriZolzap19Adult-B0003	WBL	10.00%	WBL	Match	WBL	Match	WBL	Match	Intermediate	0	Intermediate	0	WBL	Match
LriZolzap19Adult-B0005	WBL	20.00%	WBL	Match	WBL	Match	WBL	Match	Intermediate	0	Intermediate	0	WBL	Match
LriZolzap19Adult-B0006	WBL	0.00%	WBL	Match	WBL	Match	WBL	Match	WBL	Match	WBL	Match	WBL	Match
LriZolzap19Adult-B0010	WBL	20.00%	WBL	Match	WBL	Match	WBL	Match	WRL	FALSE	WRL	FALSE	WBL	Match
LriZolzap19Adult-B0011	WBL	-	-	-	-	-	-	-	-	-	-	-	-	-
LriZolzap19Adult-B0014	WBL	-	-	-	-	-	-	-	-	-	-	-	-	-
LriZolzap19Adult-B0016	WBL	20.00%	WBL	Match	WBL	Match	WBL	Match	WRL	FALSE	WRL	FALSE	WBL	Match
LriZolzap19Adult-B0017	WBL	-	-	-	-	-	-	-	-	-	-	-	-	-
LriZolzap19Adult-B0020	WBL	33.30%	WBL	Match	-	-	WBL	Match	-	-	-	-	WRL	FALSE
LriZolzap19Adult-B0024	WBL	10.00%	WBL	Match	WBL	Match	WBL	Match	Intermediate	0	Intermediate	0	WBL	Match
LriZolzap19Adult-B0027	WBL	0.00%	WBL	Match	WBL	Match	WBL	Match	WBL	Match	WBL	Match	WBL	Match
LriZolzap19Adult-B0029	WBL	37.50%	WBL	Match	-	-	Intermediate	0	Intermediate	0	Intermediate	0	WBL	Match
subtotal WBL Match	24			19		11		16		6		6		14
subtotal WBL Mismatch	-			1		2		2		2		2		2
subtotal WBL Intermediate				1		2		3		10		10		5

subtotal WBL Unknown	-			4		11		6		16		16		8
WBL ID Concordance				90.5%		73.3%		76.2%		33.3%		33.3%		66.7%
LayZolzap19Adult-S0002	WRL	100.00 %	WRL	Match	-	-	WRL	Match	WRL	Match	WRL	Match	WRL	Match
LayZolzap19Adult-S0003	WRL	80.00%	WRL	Match	WRL	Match	WRL	Match	Intermediate	0	Intermediate	0	WRL	Match
LayZolzap19Adult-S0004	WRL	100.00 %	WRL	Match	WRL	Match	WRL	Match	WRL	Match	WRL	Match	WRL	Match
LayZolzap19Adult-S0005	WRL	50.00%	Intermediate	0	-	-	Intermediate	0	-	-	-	-	Intermediate	0
LayZolzap19Adult-S0006	WRL	100.00 %	WRL	Match	WRL	Match	WRL	Match	WRL	Match	WRL	Match	WRL	Match
LayZolzap19Adult-S0009	WRL	80.00%	WRL	Match	WRL	Match	WRL	Match	WRL	Match	WRL	Match	Intermediate	0
LayZolzap19Adult-S0013	WRL	100.00 %	WRL	Match	WRL	Match	WRL	Match	WRL	Match	WRL	Match	WRL	Match
LayZolzap19Adult-S0019	WRL	30.00%	WBL	FALSE	WBL	FALSE	WBL	FALSE	WRL	Match	WRL	Match	WBL	FALSE
LayZolzap19Adult-S0020	WRL	-	-	-	-	-	-	-	-	-	-	-	-	-
LriZolzap19Adult-B0012	WRL	50.00%	Intermediate	0	-	-	Intermediate	0	WRL	Match	WRL	Match	Intermediate	0
LriZolzap19Adult-B0018	WRL	-	-	-	-	-	-	-	-	-	-	-	-	-
LriZolzap19Adult-B0021	WRL	-	-	-	-	-	-	-	-	-	-	-	-	-
LriZolzap19Adult-B0022	WRL	-	-	-	-	-	-	-	-	-	-	-	-	-
LriZolzap19Adult-B0025	WRL	100.00 %	WRL	Match	WRL	Match	WRL	Match	WRL	Match	WRL	Match	WRL	Match
LriZolzap19Adult-B0028	WRL	100.00 %	WRL	Match	WRL	Match	WRL	Match	WRL	Match	WRL	Match	WRL	Match
subtotal WRL Match	15			8		7		8		9		9		7
subtotal WRL Mismatch	-			1		1		1		0		0		1
subtotal WRL Intermediate				2		0		2		1		1		3
subtotal WRL Unknown	-			6		7		6		6		6		7
WRL ID Concordance				72.7%		87.5%		72.7%		90.0%		90.0%		63.6%
Total Ecotype ID Concordance				84.4%		78.3%		75.0%		53.6%		53.6%		65.6%

397
